# Oxidative benzylic C(sp^3^)–H functionalisation of electron-poor substrates with photoexcited DDQ

**DOI:** 10.1039/d5sc05561j

**Published:** 2025-09-19

**Authors:** Alexander P. Atkins, Charlotte A. Smith, Deborin Ghosh, Hallam J. M. Greene, Ria G. Binyahan, Ciaran J. Greene, Joseph A. Tate, Andrew J. Orr-Ewing, Alastair J. J. Lennox

**Affiliations:** a School of Chemistry, University of Bristol Cantock's Close Bristol BS81TS UK a.lennox@bristol.ac.uk; b Jealott's Hill International Research Centre Bracknell RG426EY UK

## Abstract

2,3-Dichloro-5,6-dicyano-1,4-benzoquinone (DDQ) is a common oxidant used for myriad oxidation reactions, including the functionalisation of electron-rich benzylic C(sp^3^)–H bonds. Under photochemical excitation, DDQ becomes a far more powerful oxidant, though it has scarcely been utilised for C(sp^3^)–H functionalisation nor is the mechanism by which it operates well understood. Here, we report that photochemical excitation of DDQ enables oxidative functionalisation of previously inaccessible electron-poor benzylic C(sp^3^)–H bonds to form benzylic quinol ethers. The methodology is amenable to both batch and flow setups and is demonstrated across a series of primary and secondary benzylic substrates. Transient absorption spectroscopy revealed mechanistic insight into the electron transfer processes involved, indicating a single electron transfer pathway operates under these conditions, contrasting a hydride mechanism previously reported to occur when employing ground-state DDQ.

## Abstract 2

Photochemical excitation of DDQ enables oxidative functionalisation of otherwise inaccessible electron-poor benzylic C(sp^3^)–H bonds, affording quinol ethers *via* a single electron transfer pathway.

## Graphical abstract

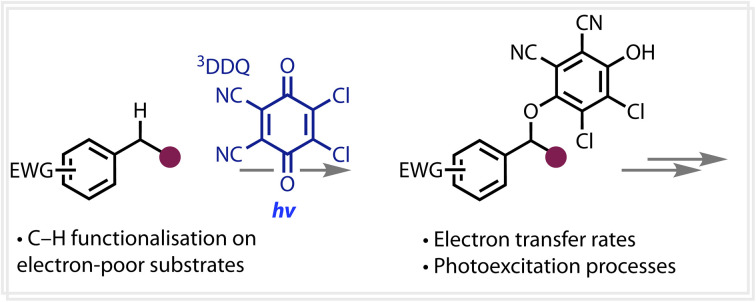

## Introduction

2,3-Dichloro-5,6-dicyano-1,4-benzoquinone (DDQ) is a powerful two-electron oxidant widely used in organic synthesis.^1,2^ The readily available and inexpensive electron deficient quinone has three redox states, quinone, semiquinone and hydroquinone, and is reported to react *via* single-electron transfer (SET), proton coupled electron transfer (PCET) or hydride abstraction mechanisms.^3–5^ It has been used stoichiometrically or as a catalyst with a sacrificial oxidant. This versatility has enabled its application in diverse contexts, from the synthesis of complex natural products to pharmaceutical intermediates,^6,7^ and in reactions, such as dehydrogenation,^8–14^ alcohol oxidation,^15–22^ cyclisation,^23–26^ cross coupling,^27,28^ and benzylic functionalisation.^29–42^

**Fig. 1:**
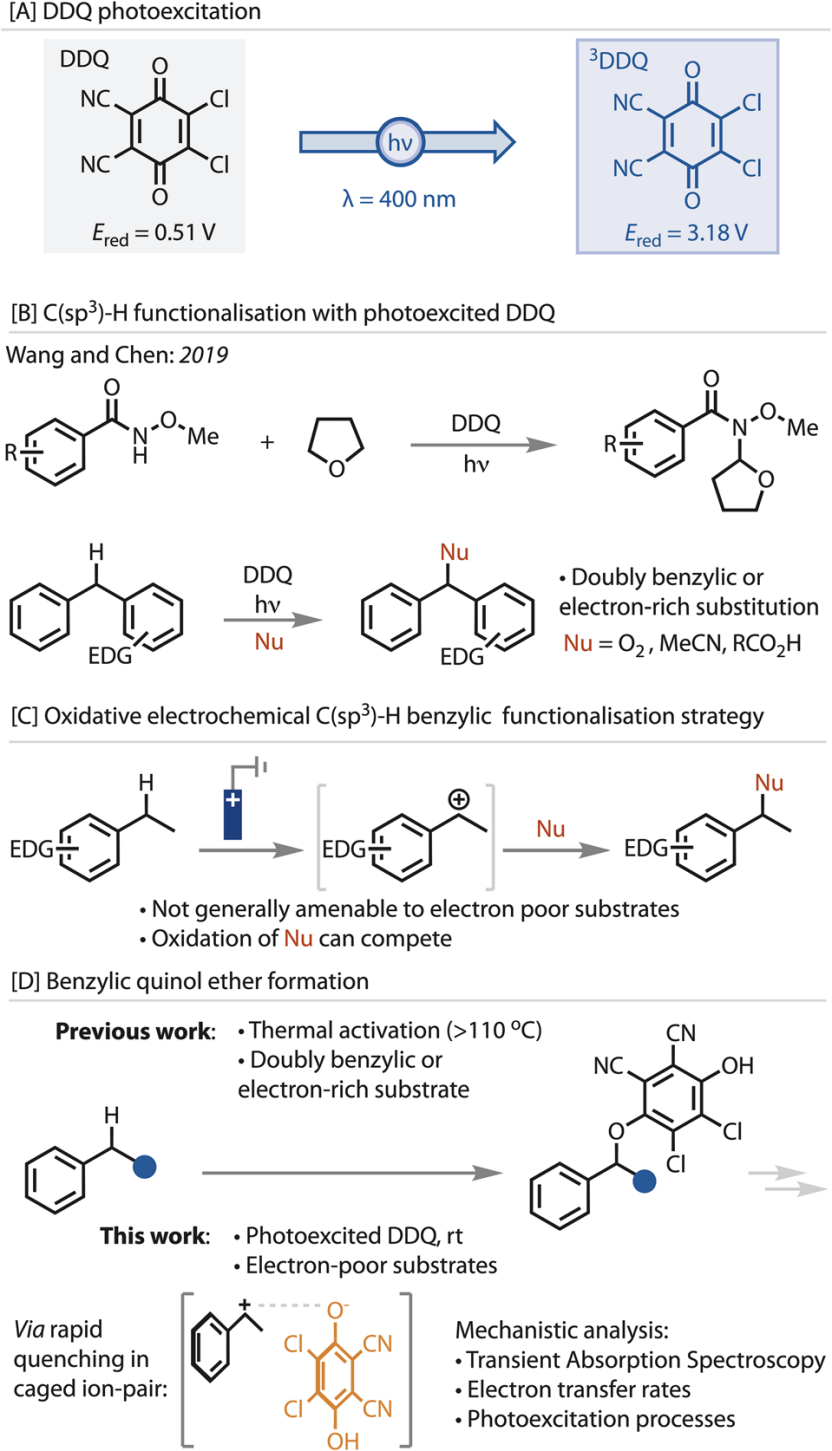
Photoexcited DDQ and its use in benzylic (Csp^3^)–H functionalisation. [A] Properties of DDQ before and after photoexcitation. Potentials quoted *vs.* SCE. [B] Previous examples of benzylic C(sp^3^)–H functionalisation with photoexcited DDQ. [C] Overview of oxidative electrochemical benzylic C(sp^3^)–H functionalisation strategy. [D] Quinol ether formation using thermal conditions or, in this work, using photochemical conditions.

In recent years, photoexcitation of DDQ has emerged as a powerful strategy for increasing its oxidation potential. Upon light absorption, a relatively long-lived triplet state (^3^DDQ*) is reported to form, increasing the oxidation potential from 0.51 V to an estimated 3.18 V *vs.* SCE,^43,44^Fig. 1A. This significant increase in oxidative power has expanded its use as a reagent enabling more difficult synthetic transformations,^2^ including C–H functionalisation. With the ever increasing need to develop more direct, greener synthesis routes, the functionalisation of C–H bonds remains an important yet formidable challenge.

Functionalisation of unsaturated C(sp^2^)–H bonds has been targeted most frequently with photoexcited DDQ, *via* the single electron oxidation of π-systems. The reactive radical cation intermediates rapidly react with nucleophiles to give functionalised products. Fukuzumi reported this strategy with the hydroxylation and then the chlorination of arenes.^45,46^ Since this pioneering work, several more examples of arene and alkene C–H functionalisation have been developed.^46–55^

The functionalisation of saturated C(sp^3^)–H bonds with photoexcited DDQ has received far less attention, Fig. 1B.^2^ Wang and Chen reported the α-functionalisation of cyclic ethers, such as tetrahydrofuran and tetrahydropyrans, in a C–H/N–H cross-dehydrogenative coupling reaction with amides.^55^ Benzylic C(sp^3^)–H bond functionalisation in this paradigm has been limited to oxygenation or amidation reactions of substrates that are either doubly benzylic or electron-rich.^56–60^

We have a long-standing interest in the functionalisation of benzylic C(sp^3^)–H bonds,^61–64^ due to its importance for pharmaceutical and agrochemical development given the high propensity of this site to undergo enzymatic oxidation.^65–67^ The strategy that we, and others, have adopted is an oxidative approach using electrochemical oxidation to generate benzylic cations.^68–80^ The advantage of this strategy is that simple and readily accessible nucleophiles, including carboxylic acids, amides, amines, alcohols and isothiocyanates, among others, can be used to quench the cation, Fig. 1C. However, a remaining challenge using this oxidative strategy is the functionalisation of electron-poor substrates, *i.e*., substrates containing electron withdrawing groups on the arene. Not only is the oxidation potential increased, which competes with oxidation of the nucleophile, but the reduced stability of the intermediate cation promotes side reactions and decomposition. These limitations have prevented their functionalisation *via* this strategy. Hence, we were interested to explore whether the increased oxidation potential of photoexcited DDQ could address this limitation.

While photoexcited DDQ is a potent oxidant able to oxidise electron-poor benzylic substrates, we proposed that, without solvent separation, the reduced hydroquinone could rapidly quench the reactive benzylic cationic intermediate to yield a benzylic quinol ether DDQ adduct, Fig. 1D. Quinol ether adducts of this kind, either from DDQ or other quinones, have been formed under thermal conditions (>110 °C) for electron-rich and -neutral toluene derivatives,^81–83^ or proposed as intermediates or isolated from reactions that always require high temperatures. These ethers are amenable to derivatisation, for example to phosphorylation, amination and oxygenation reactions.^81–88^ However, their synthesis is limited to electron-rich substrates and very high temperatures. Herein, we describe conditions suitable for a broader range of electronic properties and in-depth transient absorption spectroscopy to elucidate the electron transfer processes.

**Fig. 2:**
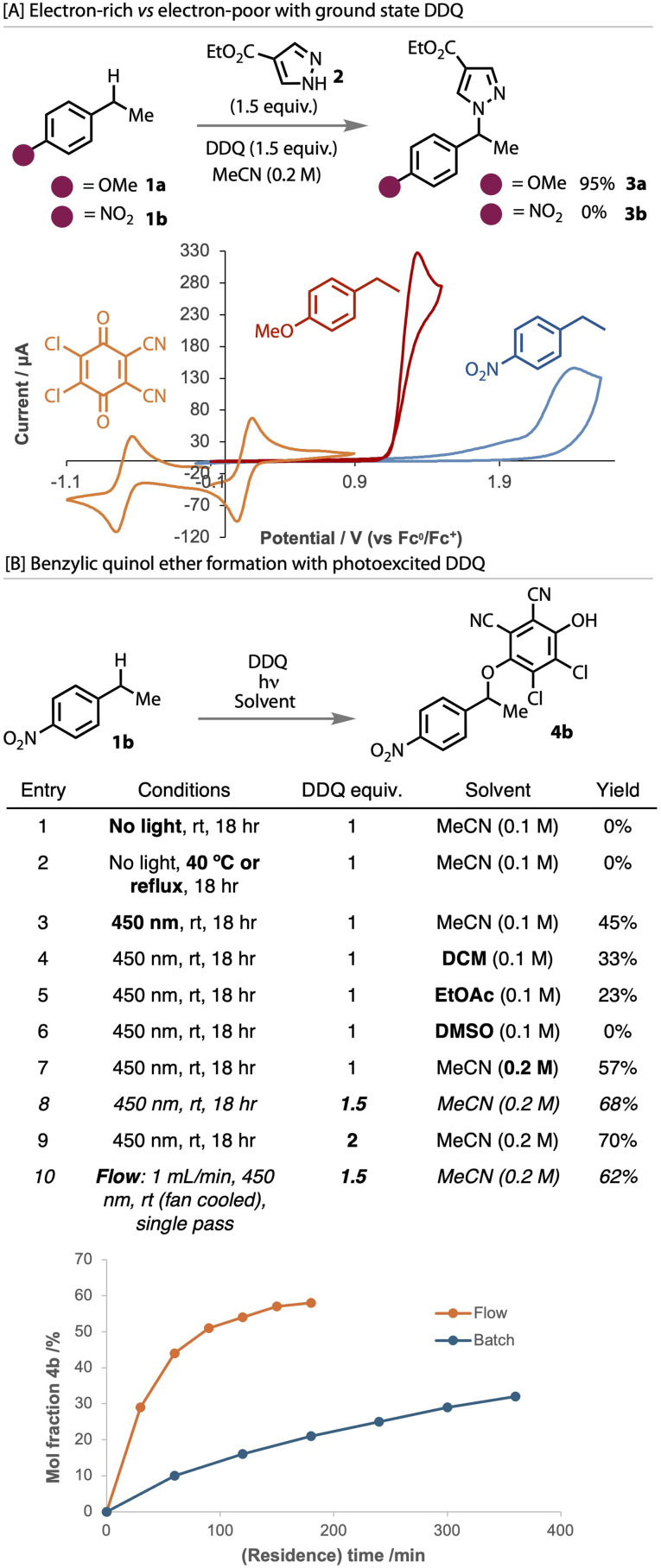
[A] Reactivity of electron-rich and electron-poor substrate with ground state DDQ. Cyclic voltammetry conditions: glassy carbon working electrode, platinum wire counter electrode, Ag/AgNO_3_ reference electrode, 0.1 M TBAPF_6_ supporting electrolyte in MeCN, 0.1 V s^−1^ scan rate. Potentials reported *vs.* Fc/Fc^+^ couple. [B] Reaction optimisation of quinol ether with photoexcited DDQ. Yields reported as ^1^H NMR yields.

To demonstrate the difference in reactivity between electron-poor and electron-rich benzylic substrates, known functionalisation conditions were applied to model substrates in the dark with ground state DDQ,^73,89^Fig. 2A. While electron-rich 4-ethylanisole 1a reacted readily in the presence of a prototypical nucleophile, pyrazole 2, to give 3a in high yield, 4-nitro(ethyl)benzene 1b, did not react and, as such, produced no product 3b. Cyclic voltammetry confirmed a 1.1 V difference in oxidation potential between 1a and 1b, thereby rationalising this reactivity. The reduction potential of DDQ is 1 V lower than that of 1a, which supports the formation of a charge transfer complex to render this a feasible process.^2,3,60,81,90^

To focus on ether (4) formation with electron-poor substrate 1b, the nucleophile was removed, Fig. 2B see SI for full optimisation table. Without light, no reaction occurred (entry 1), even when heated to reflux (entry 2). With more electron-rich substrates under these thermal conditions, the corresponding ketone was formed with no evidence of ether formation (see SI), which is consistent with that previous reported.^91^ However, upon irradiation of the reaction solution with blue light (entries 3), the desired benzylic quinol ether was indeed formed, thereby demonstrating the ability of photoexcited DDQ to oxidatively activate electron-poor 1b. Varying the solvent revealed that acetonitrile was the best solvent (entries 3–6) with a higher concentration (entry 7). The loading of DDQ was found to influence the reaction, with a large improvement with 1.5 equivalents, but only a minor further improvement with 2 equivalents (entries 8 and 9). Investigations with the use of other additives, including Lewis acids, were not found to improve the yield any further (see SI for details). The quinol ether was purified by trituration and a base wash, and isolated following drying and solvent evaporation.

We adopted a commercially available 2 mL photo-flow cell, in which the flow rate and the number of passes were assessed for optimal yield and productivity (see SI for details). It was found that a slower flow rate, increased residence time and multiple passes were beneficial. The formation of product was monitored with time, which demonstrated shorter reaction times could be applied compared to batch, due to the enhanced light penetration into the flow tubing. Hence, we constructed a single pass flow apparatus by wrapping FEP tubing around a light, and found the yield could be maintained with only a single pass.

**Fig. 3:**
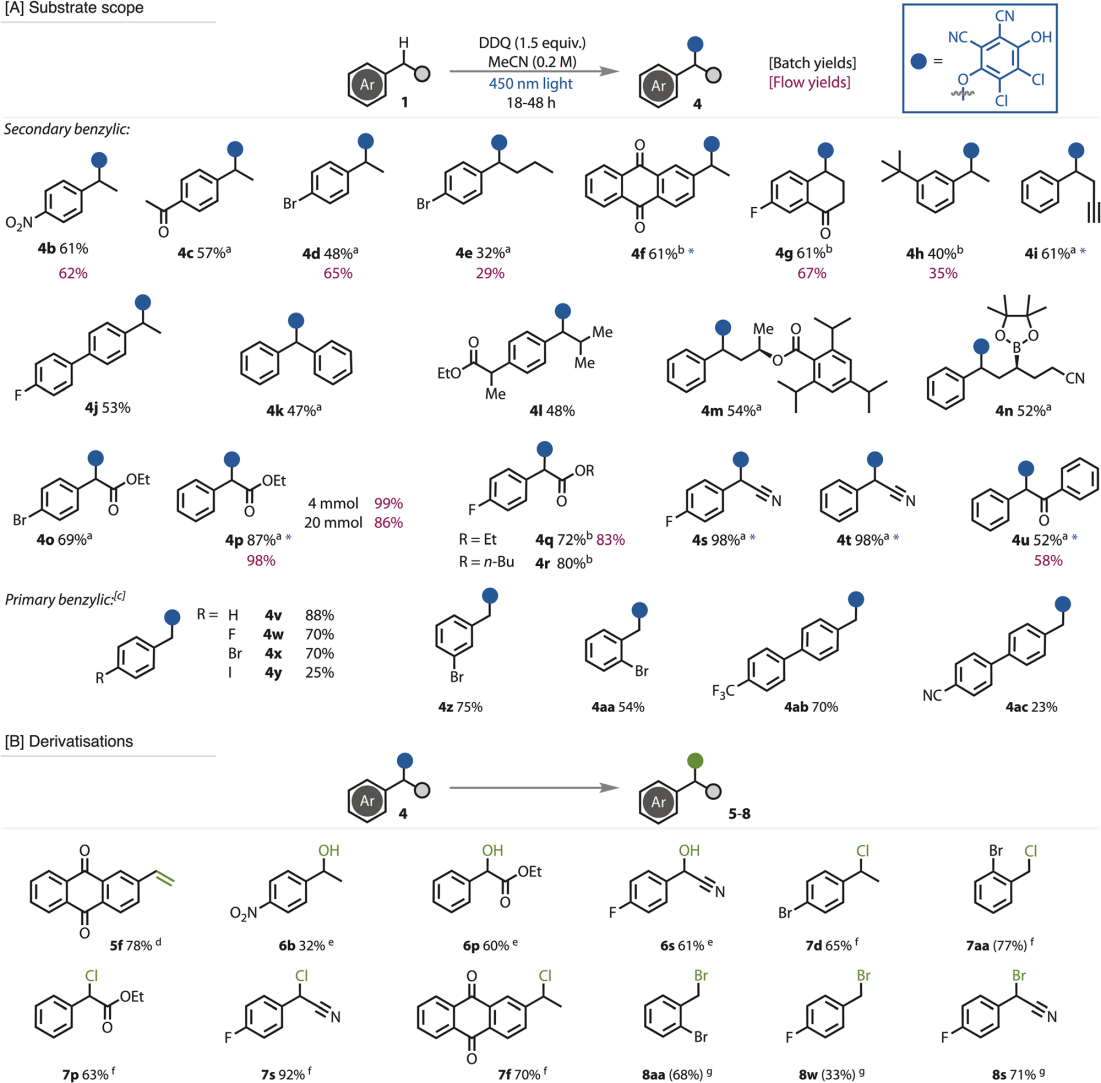
[A] Scope of benzylic functionalisation with DDQ under photochemical conditions and [B] further functionalisation of the benzylic quinol ethers. Yields reported are isolated yields, with those in parentheses calibrated GC yields due to product volatility. 0.4 mmol scale unless otherwise stated. Derivatisations were conducted at 0.1 mmol scale. Yields shown in maroon indicate when the reaction was conducted in flow. * Purified by trituration. ^*a*^Reaction run at 0.1 M concentration; ^*b*^reaction run at 0.06 M concentration with 1.0 equiv. DDQ; ^*c*^Primary benzylic conditions: DDQ (1 equiv.), substrate (3 equiv.) in DCM (0.1 M); ^*d*^DMSO, 90 °C, 18 h; ^*e*^DMSO, 90 °C, 3 h; ^*f*^MeCN, DCDMH (3 equiv.), rt, 18 h; ^*g*^MeCN, NBS (3 equiv.), 60 °C, 18 h.

We then assessed the generality of the reaction conditions by applying them to a variety of substrates, Fig. 3A. Secondary benzylic substrates with electron-withdrawing and neutral substitution were well tolerated in the reaction. Very electron-rich substrates were not successfully transformed (see SI), presumably due to reaction with ground state DDQ, which does not convert to the quinol ether product. Functional groups in peripheral positions, such as esters and a boronic ester were tolerated (4n). Substrates with electron-withdrawing groups next to the benzylic position, such as ester (4o–4r), nitriles (4s–4t) and ketone (4u), led to the desired products. Several substrates were tested in our flow set up, which was found to give comparable yields to those performed in batch. The ability to scale this chemistry up to 20 mmol (3.5 g starting material) was demonstrated on substrate 4p, which showed very little loss in overall yield.

Primary benzylic substrates are generally more difficult to functionalise due to the higher instability of the oxidised reactive intermediates, and hence are frequently used in a large excess (solvent level) when functionalised.^75,92–94^ Nevertheless, when switching the solvent from MeCN to DCM, excellent yields of the desired ether were produced with the use of only 3 equivalents of the primary benzylic substrate (see SI for full details). This strategy tolerated a variety of electron deficient substrates, including substitution with halogens (4v–4ac), trifluoromethyl and nitrile. The use of tertiary benzylic substrates did not convert to the desired ether, presumably due to enhanced steric demands (see SI).

The utility of the benzylic quinol ethers as building blocks for further functionalisation beyond those reported in the literature,^81–88^ was demonstrated in several new transformations, Fig. 3B. Extensive heating of ether 4f eliminated DDQ-H_2_ to give the alkene product 5f, while heating for a shorter period of time in DMSO led to hydroxylated products 6b, 6p, 6s. Halogenation reactions were readily initiated upon exposure to the corresponding electrophilic reagent. Hence, treatment of the quinol ethers with dichlorodimethylhydantoin (DCDMH) gave chlorination products 7d, 7f, 7p, 7s, 7aa, while *N*-bromosuccinimide (NBS) led to the corresponding bromination products 8s, 8w, 8aa each in good to excellent yields.

**Fig. 4:**
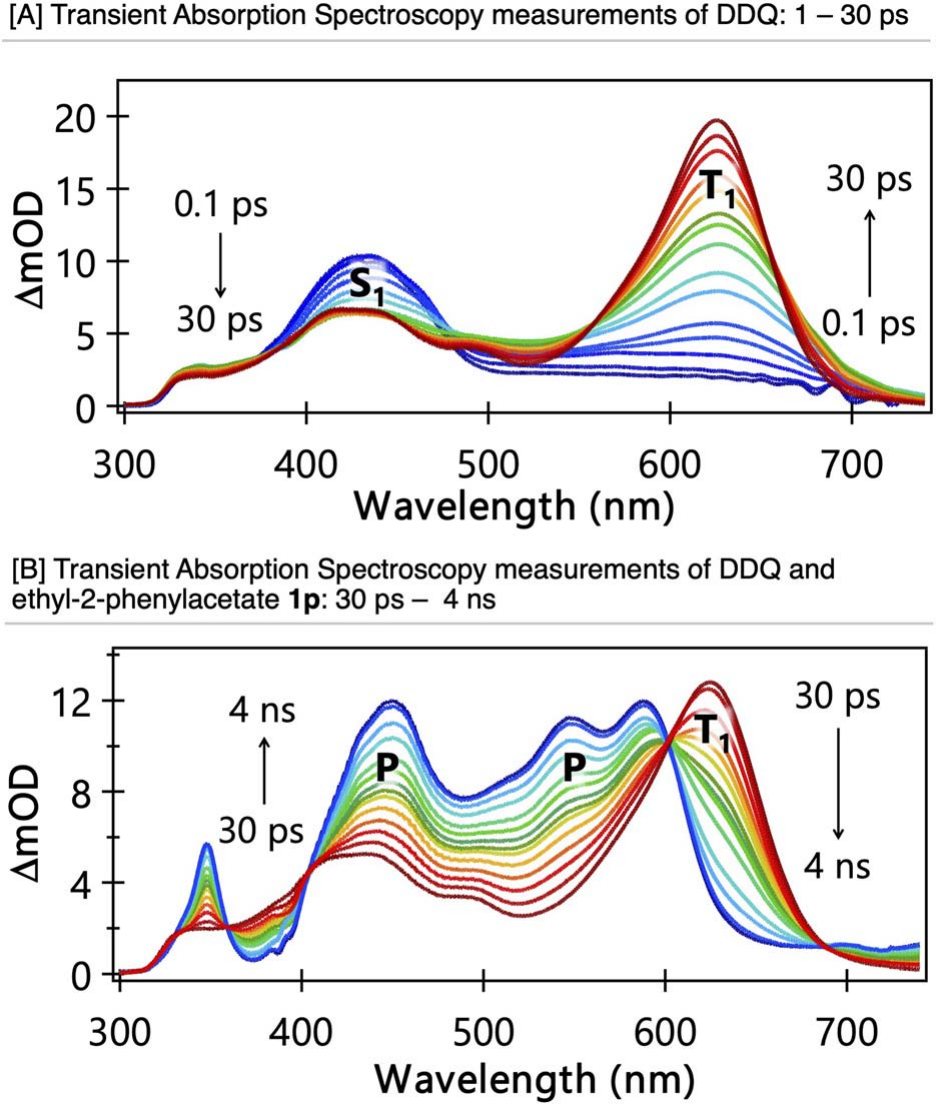
[A] TA spectra of a DDQ solution in MeCN at time delays from 100 fs to 30 ps. [B] TA spectra of DDQ solutions in MeCN at time delays from 30 ps to 4 ns in the presence of ethyl-2-phenylacetate 1p. In panels (A and B), the coloured lines represent TA spectra obtained at different time delays between sample photoexcitation at 395 nm and the white-light-continuum probe pulses. The progression of time delays is indicated by arrows in the figures—ranging from blue to red in panel (A), and from red to blue in panel (B). The band centred near 433 nm is attributed to S_1_ excited-state absorption, while the band around 625 nm is assigned to T_1_ excited-state absorption. The decay of T_1_ excited-state absorption, as shown in panel (B), is matched by growth of a broad, multi-peaked band corresponding to products (P) of the first reaction step.

To understand the mechanistic details and gain transferable insights into photoexcited DDQ, we conducted several transient absorption (TA) spectroscopy measurements.^95–98^ The TA measurements typically used DDQ concentrations of 30 mM in dry MeCN and a 100 mm sample pathlength. Care was taken to prevent any contact between the samples and metal surfaces, which otherwise contaminated the solutions with reduced forms of DDQ over the timescale of our TA measurements. Using an excitation (pump) wavelength of 395 nm, and a white-light continuum probe spanning 325–740 nm, we recorded TA spectra over pump-probe time delays from 100 fs–4 ns, with an instrument response of 120 fs. The excitation wavelength was chosen to lie near the maximum of the first electronic absorption band of DDQ (an example of which is shown in Fig. S3) and close to wavelengths accessible with the LEDs used for synthetic photochemistry studies. At time delays up to about 30 ps, rapid evolution of the TA spectrum within our probe wavelength window revealed loss of intensity in bands assigned to excited state absorption (ESA) from the S_1_ state, and growth of ESA bands attributed to population of the T_1_ state. The electronically nonadiabatic dynamics of this fast intersystem crossing are discussed in more depth in a separate report. Example TA spectra are shown in Fig. 4A; the S_1_ ESA is evident as a single band centred near 433 nm, while two distinct bands centred at 425 nm and 625 nm have matching kinetics and are assigned to T_1_ ESA. At time delays beyond 30 ps, solutions of DDQ in MeCN showed no significant further changes to the T_1_ ESA band over the 4 ns range accessible in our experiment.

**Fig. 5:**
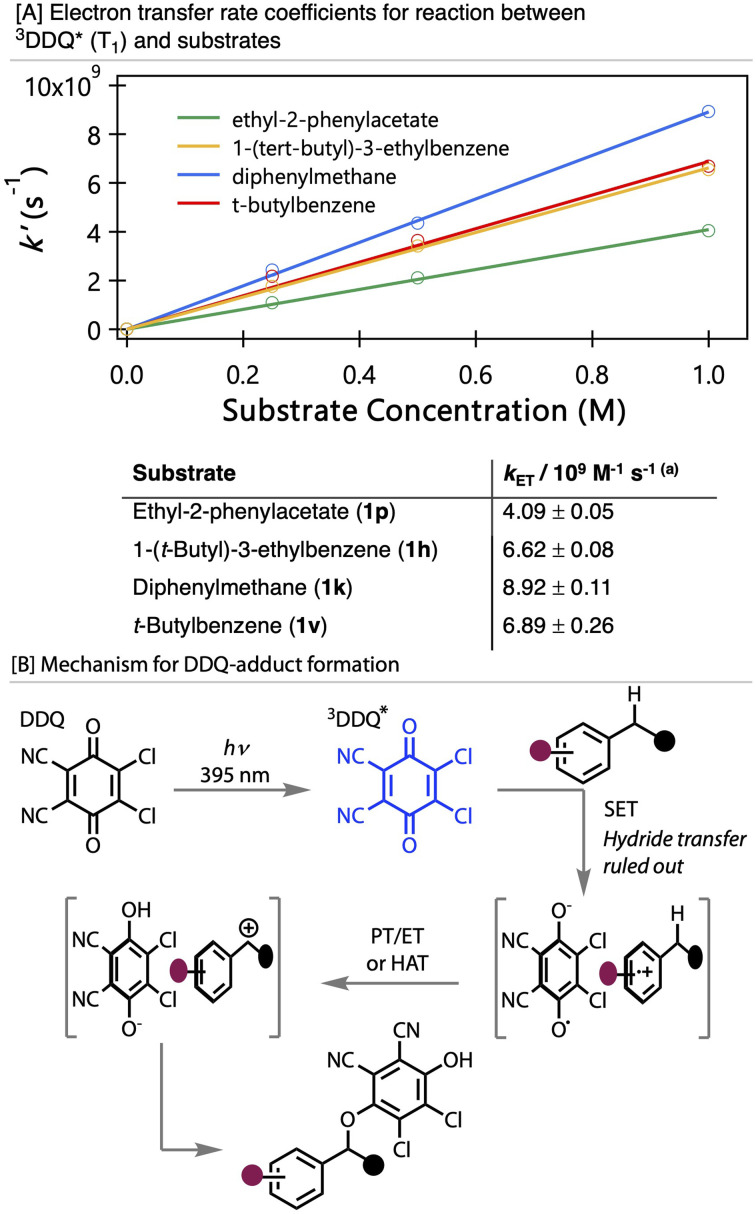
[A] Plots of the concentration dependence of pseudo-first-order rate constants (*k¢*) for reactions between ^3^DDQ* and four different substrates, with linear fits to obtain bimolecular rate coefficients. Measurements recorded in acetonitrile at 20 °C. The uncertainties specified in *k*_ET_ values are only those from linear fits to concentration-dependent pseudo-first-order rate coefficients and do not include possible systematic errors, for example in the substrate concentrations in the studied samples. [B] proposed mechanism based on TAS data.

Reaction of photoexcited DDQ with various substrates was then explored using ethyl-2-phenylacetate (1p, Fig. 3), 1-(*tert*-butyl)-3-ethylbenzene (1h), diphenylmethane (1k), and control experiments with *t*-butyl benzene (containing no benzylic C–H) and 4-cyanophenylacetontrile (1v), which was an unsuccessful substrate (see SI). With the exception of 4-cyanophenylacetonitrile 1v, addition of a primary benzylic substrate to the solution resulted in decay of the T_1_ ESA bands and growth of a new, broad absorption feature spanning 325–740 nm (Fig. 4B and S4(b) to (d)). For higher substrate concentrations, this conversion is complete on the 4 ns timescale of our TA spectroscopy measurements, which allows the time-dependent contributions of these two distinct spectral features to be extracted by fitting to early and late delay-time spectral basis functions representing the T_1_ ESA and product absorption, respectively. This spectral decomposition was carried out using our KOALA software.^99^ The time-dependencies of the integrated band intensities for T_1_ ESA decay and product absorption growth can then be fitted with matching exponential time constants (*t*), the reciprocals of which (*k*′ = 1/*t*) are first-order rate coefficients that scale linearly with the concentration of the substrate (Fig. 5A). This concentration-dependent behaviour is indicative of pseudo-first-order reaction kinetics, with the substrate concentration greatly in excess of the concentration of ^3^DDQ*. Reaction can be attributed to the T_1_ state of photoexcited DDQ*, not the S_1_ state, because the timescale for bimolecular reaction is much longer than that for population of ^3^DDQ* by ISC.

The product absorption feature that develops over the course of the studied reactions is the same for the different substrates used, and the locations and relative intensities of the partially overlapping bands (348, 432, 458, 508, 547 and 589 nm) are an excellent match to the absorption spectrum for the DDQ˙^−^ radical anion reported by Miller *et al.*^100^ We, therefore, assign the products of the first reaction step between ^3^DDQ* and our chosen benzylic substrates to be this DDQ˙^−^ radical anion and, by inference, a radical cation of the substrate. This assignment is supported by observation of the same product bands when *t*-butylbenzene was used as the substrate, which rules out hydride ion or hydrogen atom transfer from a benzylic C–H as the operative mechanism. Hence, electron transfer from the substrate to ^3^DDQ* is identified as the first step in the reaction leading to benzylic C–H activation. This ET mechanism is inhibited when 4-cyanophenylacetontrile 1v is used as a substrate, as it is too electron-poor to be oxidised even by photoexcited DDQ.

Measurements of pseudo-first-order rate coefficients (*k*′ = *k*_ET_[S]) at different concentrations [S] of the benzylic or *t*-butylbenzene substrate, and linear fits to plots of *k*′ against [S] gave values for the bimolecular rate coefficients *k*_ET_ for the electron transfer reactions of ^3^DDQ* in MeCN solution. Example kinetic plots are shown in Fig. 5A, which summarizes the electron transfer rate coefficients obtained from this analysis. The measured electron transfer rate coefficients are consistent with diffusion limited reactions in acetonitrile, Fig. 5A, and they are in good agreement with a rate coefficient value of *k* = (5.3 ± 0.3) × 10^9^ M^−1^ s^−1^ reported by Ohkubo *et al.* for reaction of photoexcited DDQ with benzene.^45^

These studies confirm C(sp^3^)—H functionalisation with photoexcited DDQ occurs *via* a single electron oxidation pathway, Fig. 5B. This directly contrasts the hydride transfer mechanism that has been previously proposed for ground state C(sp^3^)–H functionalisation by DDQ and other benzoquinones.^3,81,101–105^ Confirmation of this mechanistic deviation under photochemical conditions is important is for further developing these kinds of transformations and widening the classes of molecules that can be functionalised by photoexcited DDQ.

## Conclusions

We employed photoexcited DDQ as a powerful single electron oxidant to functionalise benzylic C(sp^3^)–H bonds of electron-poor substrates. Access to a variety of primary and secondary benzylic hydroquinone ethers is demonstrated in batch and flow on different reaction scales. Transient absorption spectroscopy revealed mechanistic insights, showing the reaction proceeds *via* single electron oxidation by triplet state DDQ. The TA spectroscopy results indicate a different mechanism is in operation under photochemical conditions, compared to the known hydride transfer mechanism for ground-state DDQ. These insights are important for further developing methodologies with photoexcited DDQ and will assist in continuing to expand the types of substrates that can be functionalised with this approach.

## Author contributions

A. P. A., C. A. S., D. G., H. J. M. G., R. G. B., and C. J. G. performed the experimental work. A. P. A., J. A. T., and A. J. J. L. conceived the project. A. J. J. L. and A. J. O. directed the work. A. P. A., A. J. O. and A. J. J. L. wrote the manuscript.

## Conflicts of interest

There are no conflicts to declare.

## Supplementary Material

SC-016-D5SC05561J-s001

## Data Availability

The data supporting this article have been included as part of the SI. Supplementary information contains reaction procedures, characterisation data for isolated compounds, NMR and mechanistic data. See DOI: https://doi.org/10.1039/d5sc05561j.
