# Supplementary material for: Oxidative benzylic C(sp3)–H functionalisation of electron-poor substrates with photoexcited DDQ
Source: Chem Sci. 2025 Sep 19;16(41):19280–7. doi: 10.1039/d5sc05561j (PMC12447515; doi:10.1039/d5sc05561j)
Supplement: SC-016-D5SC05561J-s001 [file SC-016-D5SC05561J-s001.pdf]

Supplementary Information (SI) for Chemical Science.
This journal is © The Royal Society of Chemistry 2025

Oxidative Benzylic C(sp^3)–H Functionalisation of Electron-poor Substrates with Photoexcited DDQ

Alexander P. Atkins,[a] Charlotte A. Smith,[a] Deborin Ghosh,[a] Hallam J. M. Greene,[a] Ria G. Binyahan,[a] Ciaran J. Greene,[a] Joseph A. Tate,[b] Andrew J. Orr-Ewing,[a] Alastair J. J. Lennox [a]*

[a] School of Chemistry, University of Bristol, Cantock's Close, Bristol, BS81TS, United Kingdom

[b] Syngenta, Jealott's Hill International Research Centre, Bracknell, RG426EY, United Kingdom

Corresponding Author Email: a.lennox@bristol.ac.uk

Contents

General Experimental Details 3

Ultrafast Laser Spectroscopy 7

Steady-State UV-Vis absorption of DDQ in MeCN 7

TA Spectra of DDQ solutions in MeCN 8

Synthesis of Ethyl 1-(1-(4-methoxyphenyl)ethyl)-1*H*-pyrazole-4-carboxylate (3a) 9

Optimisation Studies 10

Secondary Benzylic Substrates 10

Flow Reactions 18

Primary Benzylic Substrates 20

Further Functionalisation Screening 22

Nucleophilic Displacement Screening 22

Electrochemical Displacement Screening 23

Halogenation Screening Reactions 24

General Reaction Procedures 25

General Procedure 1: Batch Conditions for Secondary Benzylic Substrates 25

General Procedure 2: Batch Conditions for Primary Benzylic Substrates 26

General Procedure 3: Single Pass Flow 27

General Procedure 4: Hydroxylation of DDQ-Adducts 28

General Procedure 5: Halogenation of DDQ-Adducts 29

Unsuccessful Substrates 30

Characterisation Data 31

Secondary Benzylic Substrates 31

Primary Benzylic Substrates 51

Further Functionalisation Characterisation Data 59

NMR Spectra of Novel Compounds 71

References 110

General Experimental Details

Techniques

Additions of <200 µL were made with Gilson Pipetteman pipettes. Solvents were removed from samples using a rotary evaporator which was powered by a diaphragm pump working under a pressure of 10 mmHg alongside a water bath set at 45 °C. Samples that required further drying were dried under reduced pressure using a Schlenk line.

Reagents

All syntheses were conducted open to air and in reagent grade solvents. Unless otherwise stated, reagents were purchased from commercial suppliers and used without further purification. Syntheses of substrates **1j** and **1**l is described in a previous publication.[1]

Chromatography

Thin layer chromatography was performed on SiO_2 coated aluminium plates and visualised by ultraviolet (UV) fluorescence. The compounds of interest were sufficiently visible under UV light and did not require further staining. Technical grade solvents and silica gel (230-400 mesh, 60 Å pore size) were used for column chromatography or via a Biotage Selekt instrument with Sfär Silica D Duo capsules.

Analysis

NMR samples were submitted in MeCN-d_3, DMSO-d_6 or Acetone-d_6 purchased from commercial suppliers. Spectra were recorded on Brucker Nano 400, Jeol ECS 300, Jeol ECS 400 and Jeol ECZ 400, Bruker Advance III HD 500 cryo spectrometers. Chemical shifts are reported in parts per million (ppm). Coupling constants (*J*) are quoted in Hz. Multiplicities are reported as s (singlet), d (doublet), t (triplet), q (quartet), hept (heptet), m (multiplet) or combinations thereof.

IR analysis was performed on a PerkinElmer Spectrum 100 FTIR with an ATR accessory and frequencies reported in wavenumbers (cm^{-1}).

High Resolution Mass Spectrometry (HRMS) was recorded on QExactive (GC-Orbitrap), Orbitrap Elite (LC-Orbitrap) or Synapt G2S (IMS-Q-TOF) instruments at the university's mass spectrometry facility.

Liquid chromatography-mass spectrometry (LCMS) analysis was performed using a Agilent Infinity 1260 series instrument with a single quadrupole mass spectrometer (ESI ion source). Mass spectra were recorded in both positive and negative ion mode. A Xterra MS C18 (3.5 µM, 3.0 x 20 mm IS) column was used as the stationary phase. MeCN, or H2O, with 0.1% (v/v) formic acid, and combinations thereof were used as the mobile phase.

Electrochemical Considerations

Cyclic voltammetry (CV) analysis was performed using a PalmSens MultiSens4 potentiostat with a glassy carbon disk working electrode, platinum wire counter electrode and 0.1 M Ag/$AgNO_3$ reference electrodes. All electrodes were polished before use and the reaction mixtures stirred and degassed by a stream of N_2 gas for approximately 2 min before each experiment. Each CV was then referenced to the Fc/Fc^+ redox couple by adding ferrocene (a spatula tip's amount) to the reaction mixture and rerunning the CV.

Photochemical Reaction Considerations

Photochemical reactions were conducted using a Kessil A160WE Tuna Blue lamp or a Penn PhD Photoreactor M2 with 450 nm, 420nm or 365 nm light sources. All photochemical reactions were subjected to cooling from a desk fan or internal cooling (Penn PhD Photoreactor M2). Photochemistry in flow was conducted using a commercially available flow cell[2] or with a custom-made flow setup (see below) with 6 mL path length and was powered by blue LEDs purchased from Amazon.

Descriptions of Reaction Setups

Batch Reactions

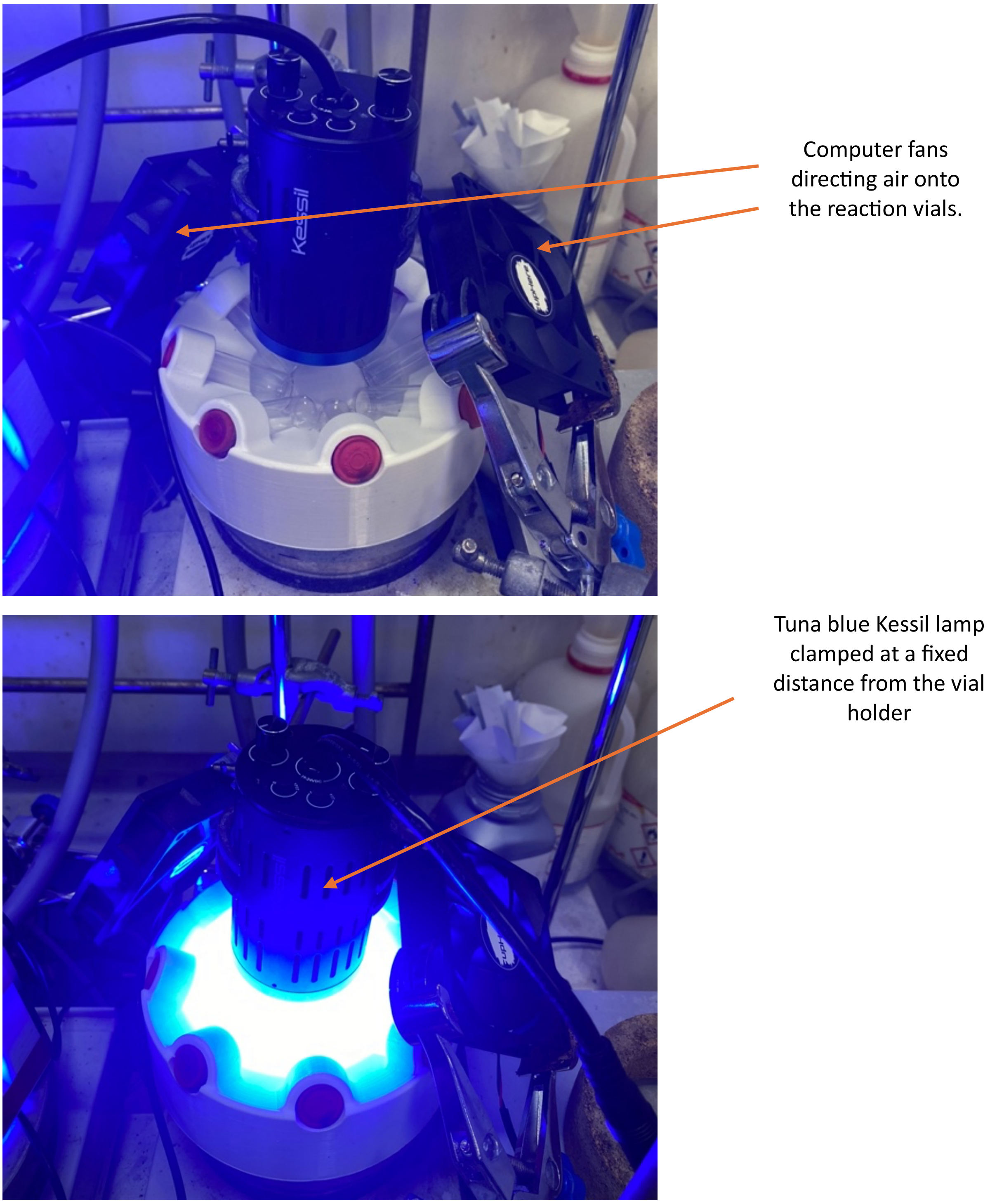

Figure S1 Batch Reaction Setup.

Flow Reactions

Flow reactions were conducted using either a commercial flow cell sold by IKA (CDR.PP narrow), previously reported by Hilton,[2] or in single pass flow using the setup illustrated and described below.

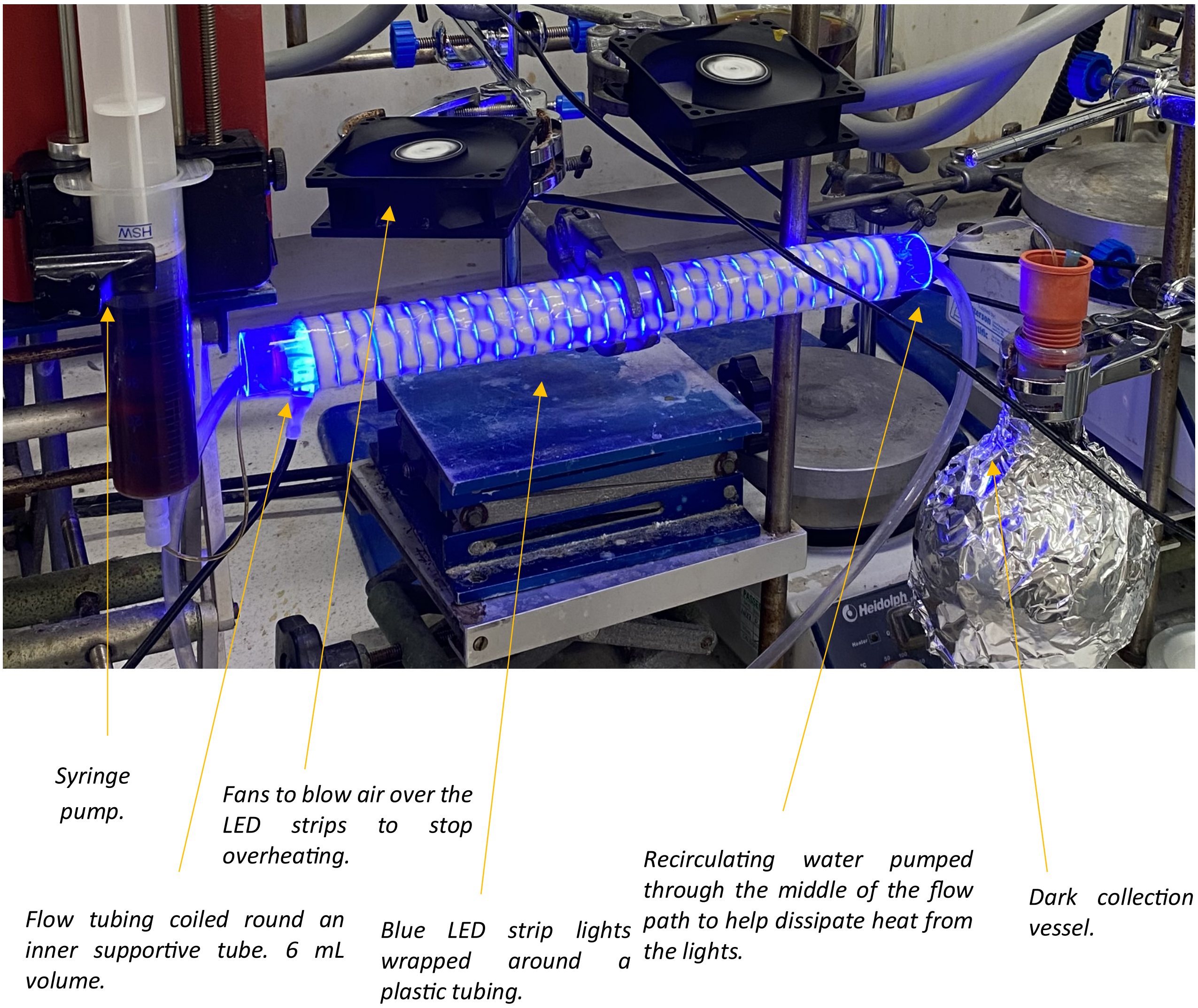

Figure S2. Single Pass Flow Reactor Setup

Ultrafast Laser Spectroscopy

The ultrafast transient absorption spectroscopy experiments used a custom-built instrument located at the University of Bristol. An amplified Ti:sapphire laser system (Coherent Astrella, 7W, 1 kHz) produced 35-fs duration, 800-nm wavelength laser pulses which were split into three separate beam paths, two of which were used in the current study. Approximately 3W of laser power pumped an optical parametric amplifier (OPA, Coherent OPerA Solo) configured to generate 395-nm pulses for sample excitation. The repetition rate of these pulses was reduced to 500 Hz using a rotating optical chopper for accumulation of pump-on and pump-off absorption spectra. A second 800-nm wavelength beamline was used to generate white-light continuum (WLC) probe pulses at a repetition rate of 1 kHz by gentle focusing into a flat CaF_2 optic. The WLC spanned wavelengths from 325 – 740 nm. The UV pump and WLC probe pulses were spatially and temporally overlapped at the sample where the pump pulses were gently focused to a spot size of ~250 μm, while the probe pulses were focused by an off-axis parabolic mirror (OAPM) to half this area. UV pump pulse energies were approximately 200 nJ at the sample. The beam path for the pump pulses included a motorized optical delay stage to introduce time delays of up to 3.75 ns before the arrival of the WLC probe pulses. The polarization angle between the linearly polarized pump and probe pulses was set to the magic angle (54.7°) to eliminate contributions from rotational diffusion. After passing through the sample, the probe pulses were recollimated by a second OAPM and then dispersed onto a 1024-element photodiode array (Entwicklungsbüro Stresing) using an Andor Shamrock 163 spectrometer. Data collection from this array detector and subsequent processing, including averaging and chirp correction, used custom-written LabVIEW software.

Steady-State UV-Vis absorption of DDQ in MeCN

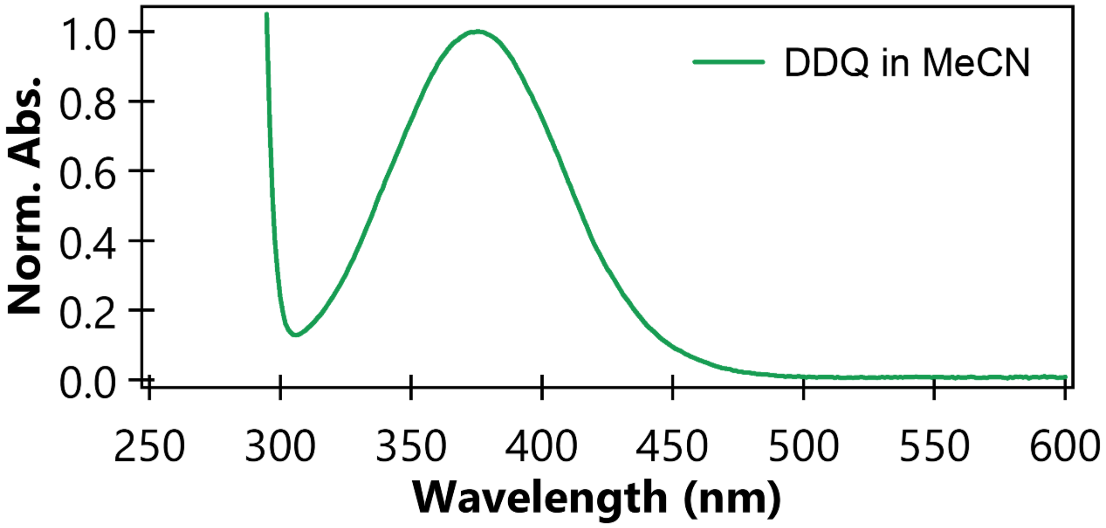

Figure S3. Steady-state UV-vis absorption spectrum of a 30 mM DDQ solution in MeCN, normalized to 1.0 at 375 nm. When reduced forms of DDQ are also present in the sample, further absorption is observed to longer visible wavelengths. These reduced forms can also be photoexcited by blue LED lamps commonly used in synthetic photochemistry procedures.

TA Spectra of DDQ solutions in MeCN

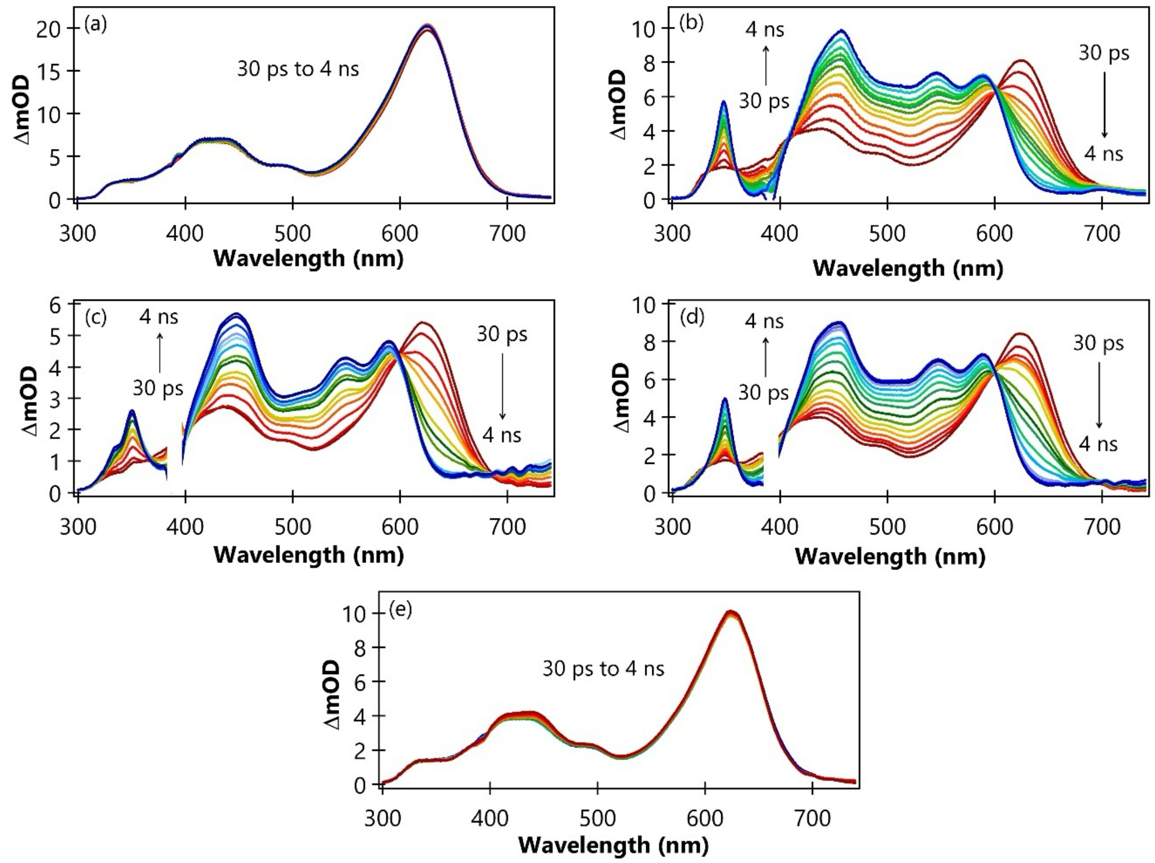

Figure S4. TA spectra of DDQ solutions in MeCN at time delays from 30 ps to 4 ns in (a) the absence of any substrate; and in the presence of (b) 1-(tert-butyl)-3-ethylbenzene, (c) diphenylmethane, (d) t-butylbenzene, and (e) 4-cyanophenylacetonitrile. The coloured lines represent TA spectra obtained at different time delays between sample photoexcitation at 395 nm and the white-light-continuum probe pulses, with increasing time delay from red through to blue colours. The band centred at 625 nm is assigned to ^{3}DDQ* (T_1) excited-state absorption, and its decay is matched by growth of a broad, multi-peaked band corresponding to products of the first reaction step. In panels (c) and (d), the spectra are masked in a narrow range of wavelengths around 395 nm where pump laser scatter contributes.

Synthesis of Ethyl 1-(1-(4-methoxyphenyl)ethyl)-1*H*-pyrazole-4-carboxylate (3a)

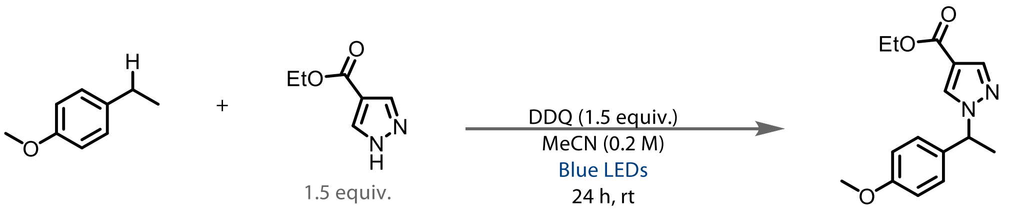

To a 10 mL Schlenk tube equipped with a magnetic stirrer bar was added 4-ethylanisole (54.5 mg, 1.00 equiv., 0.400 mmol), DDQ (136 mg, 1.50 equiv., 0.600 mmol) and ethyl 1*H*-pyrazole-4-carboxylate (84.1 mg, 1.50 equiv., 0.600 mmol). To this was added MeCN (2 mL) and the reaction mixture was stirred until all reagents were dissolved. The reaction mixture was then degassed by sparging with N_2 gas for 10 minutes. The reaction mixture was then subjected to blue light irradiation for 24 hours After this time, the solvent was removed under reduced pressure and the crude reaction mixture was purified by column chromatography (0-40% EtOAc in pentane) to afford a brown oil (96 mg, 87%). An NMR yield of 95% was observed before purification.

^{1}H NMR (400 MHz, $CDCl_3$) δ 7.91 (s, 1H), 7.83 (s, 1H), 7.21 – 7.14 (m, 2H), 6.90 – 6.83 (m, 2H), 5.45 (q, *J* = 7.0 Hz, 1H), 4.24 (q, *J* = 7.1 Hz, 2H), 3.77 (s, 3H), 1.86 (d, *J* = 7.1 Hz, 3H), 1.30 (t, *J* = 7.1 Hz, 3H).

^{13}C NMR (101 MHz, $CDCl_3$) δ 163.2, 159.5, 140.9, 132.6, 131.1,127.9, 114.9, 114.3, 61.2, 60.1, 55.3, 21.3, 14.4.

HRMS (ESI+) calc: $[M+H]^+$ ($C_{15}H_{19}N_2O_3$) 275.1390; measured: 275.1387 = 1.1 ppm error.

IR (neat) ν_{max}/cm^{-1}: 3127, 2981, 2936, 2837, 1712, 1552, 1514, 1408, 1245, 1197, 1028, 834, 769.

Optimisation Studies

Secondary Benzylic Substrates

Effect of Heating

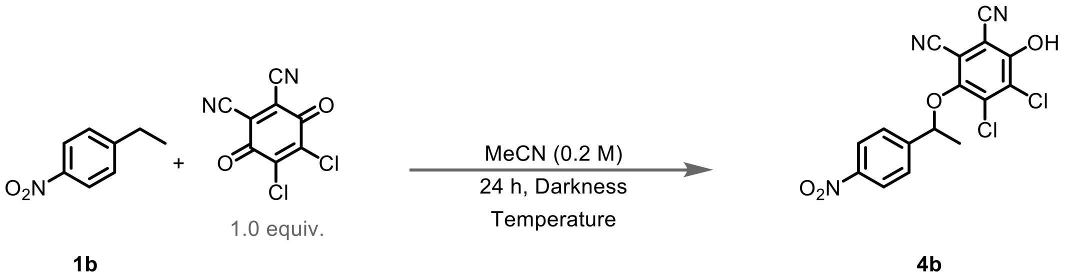

Entry	Temperature / °C	Yield 4b / %
1[a]	rt	0
2	40	0
3	85 (reflux)	0

0.4 mmol scale. Yields determined by ^{1}H NMR using mesitylene as a standard. [a] substrate at 0.1 M concentration and 18-hour reaction time.

Table S1. Attempts to prepare **4b** thermally

Solvent Screen

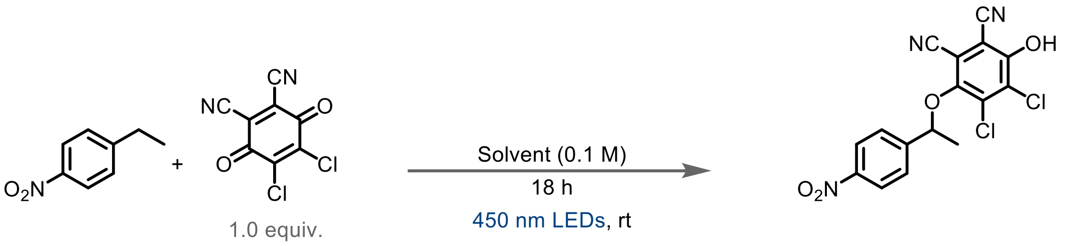

1b **4b**

Entry	Solvent	Yield 4b / %
1	MeCN	45
2	DCM	33
3	EtOAc	23
4	DME	3
5	DMSO	0
6	DCE	29
7	Toluene	0

0.4 mmol scale. Conducted on a Penn PhD Photoreactor M2 with fan cooling set to 100%. Yields determined by 1H NMR using mesitylene as a standard.

Table S2. Effect of solvent on reaction yield

Reaction Time Screen

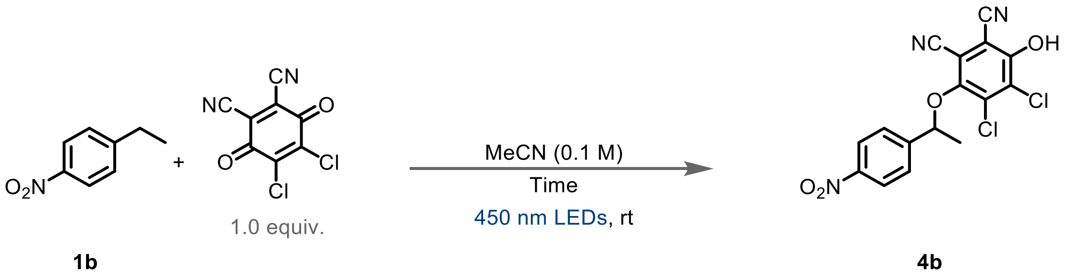

Entry	Time	Yield 4b / %	Remaining 1b / %
1	18 h	45	53
2	24 h	53	40
3	48 h	55	34

0.4 mmol scale. Conducted on a Penn PhD Photoreactor M2 with fan cooling set to 100%. Yields determined by ^{1}H NMR using mesitylene as a standard.

Table S3. Effect of time on reaction outcome

Concentration Screen

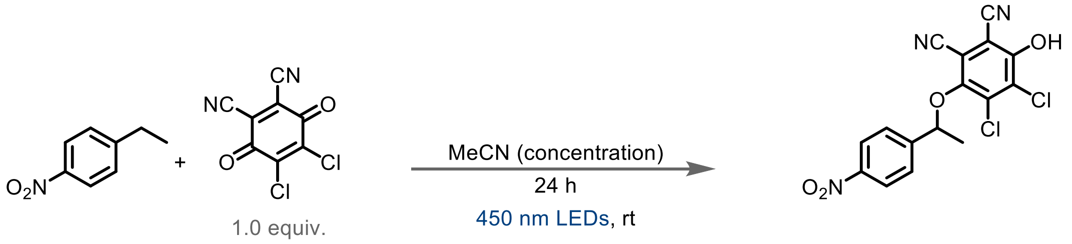

1b **4b**

Entry	[1b] / M	Yield 4b / %	Remaining 1b / %
1	0.1	53	40
2	0.2	57	40
3	0.5	50	43

0.4 mmol scale. Conducted on a Penn PhD Photoreactor M2 with fan cooling set to 100%. Yields determined by ^{1}H NMR using mesitylene as a standard.

Table S4. Effect of **1b** concentration on reaction outcome

DDQ Loading Screen

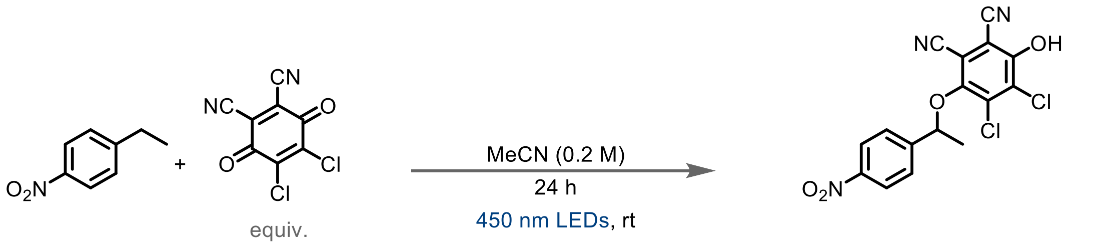

1b **4b**

Entry	DDQ Equivalents	Yield 4b / %	Remaining 1b / %
1	2.0	70	37
2	1.5	68	41
3	1.0	57	40
4[a]	1.2	57	40

0.4 mmol scale. Conducted on a Penn PhD Photoreactor M2 with fan cooling set to 100%. Yields determined by ^{1}H NMR using mesitylene as a standard. [a] with 0.2 equiv. *tert*-butyl nitrite under air.

Table S5. Effect of DDQ loading on reaction outcome

Lewis Acid Screening

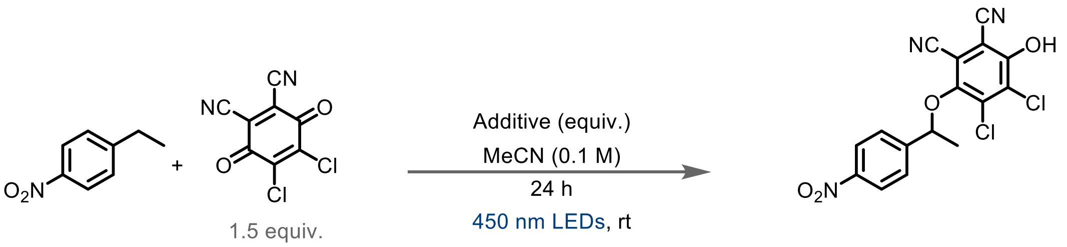

1b **4b**

Entry	Additive	Additive equiv.	Yield 4b / %
1	$AlCl_3$	0.2	38
	$AlCl_3$	0.6	40
	$AlCl_3$	1.0	37
2	$ZnCl_2$	0.2	27
3	TMSOTf	0.2	31
4	$FeCl_3$	0.2	41

0.4 mmol scale. Yields determined by ^{1}H NMR using mesitylene as a standard.

Table S6. Lewis acid screening and effect on reaction outcome

Concentration Screening

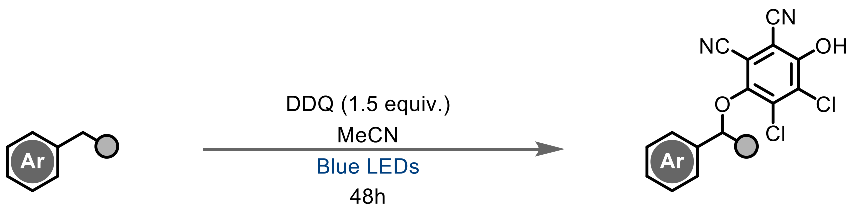

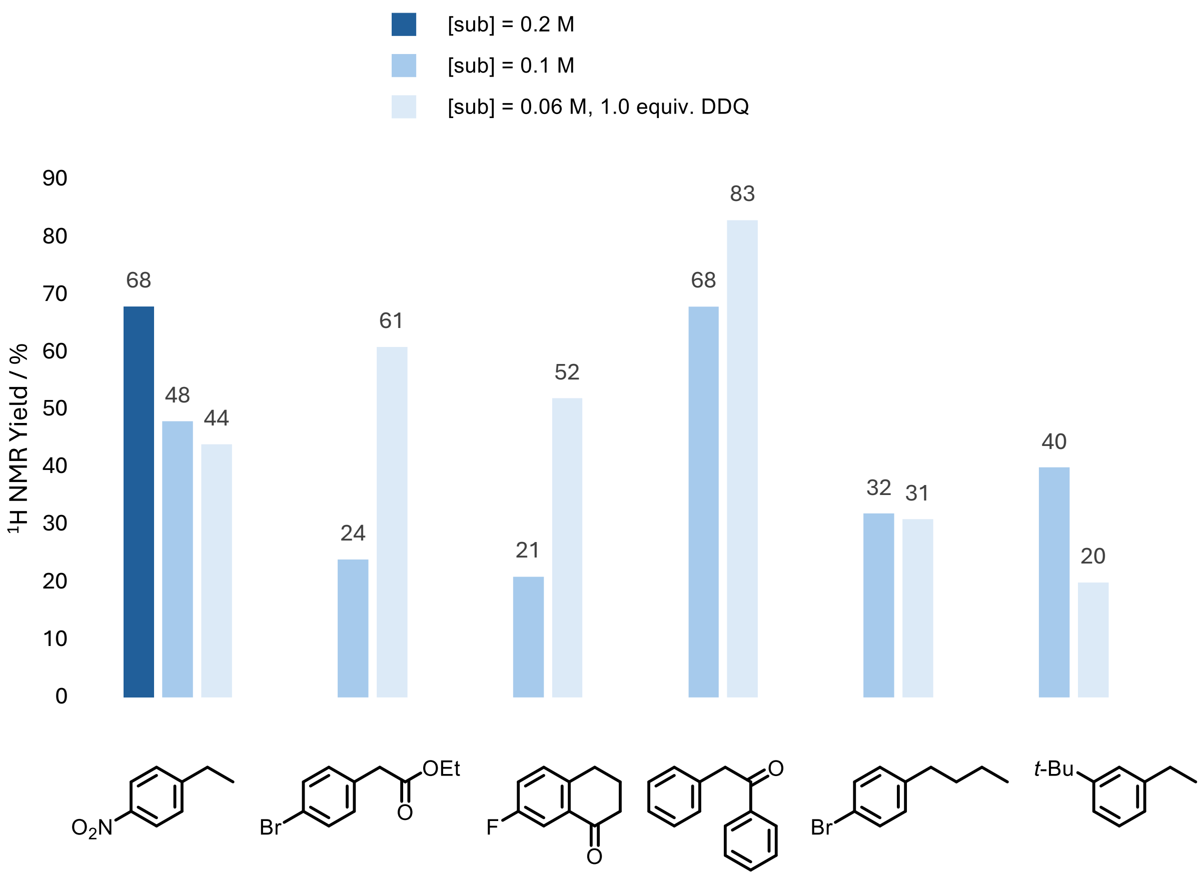

Figure S5. Comparison of different reaction conditions across different substrates. Some substrates performed better at lower concentrations, hence, were run at these concentrations.

Reaction with Electron-Rich Substrate

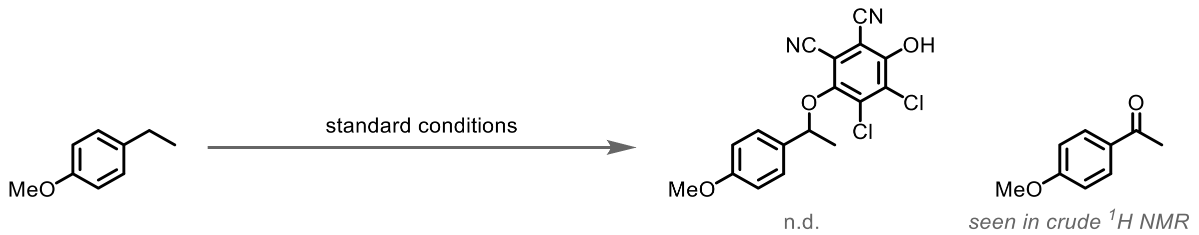

The desired DDQ-adduct was not detected by LCMS or ^{1}H NMR analysis for 4-methoxyethyl benzene substrate. Evidence of ketone formation was observed:

[crude] **^{1}H NMR** (400 MHz, Acetonitrile) δ = 7.92 (d, *J*=8.9, 2H), 2.50 (s, 3H).

Ketone peaks were assigned in the crude NMR based on previous literature data.[3]

Flow Reactions

Effect of Flow Rate

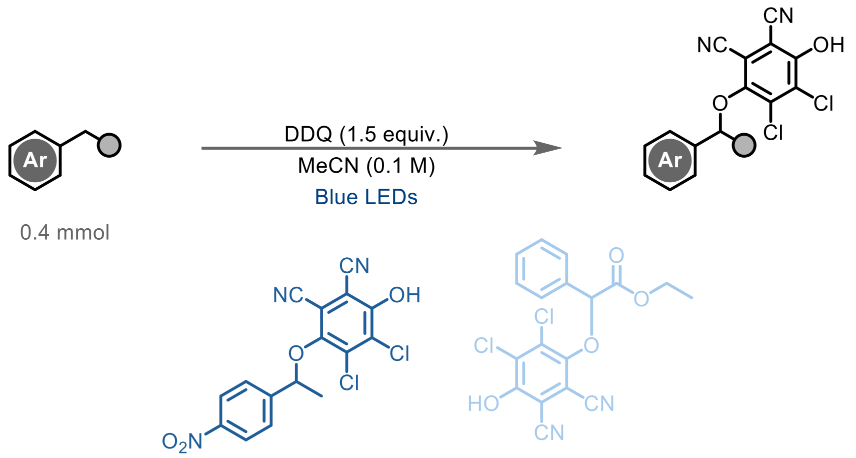

4b **4p**

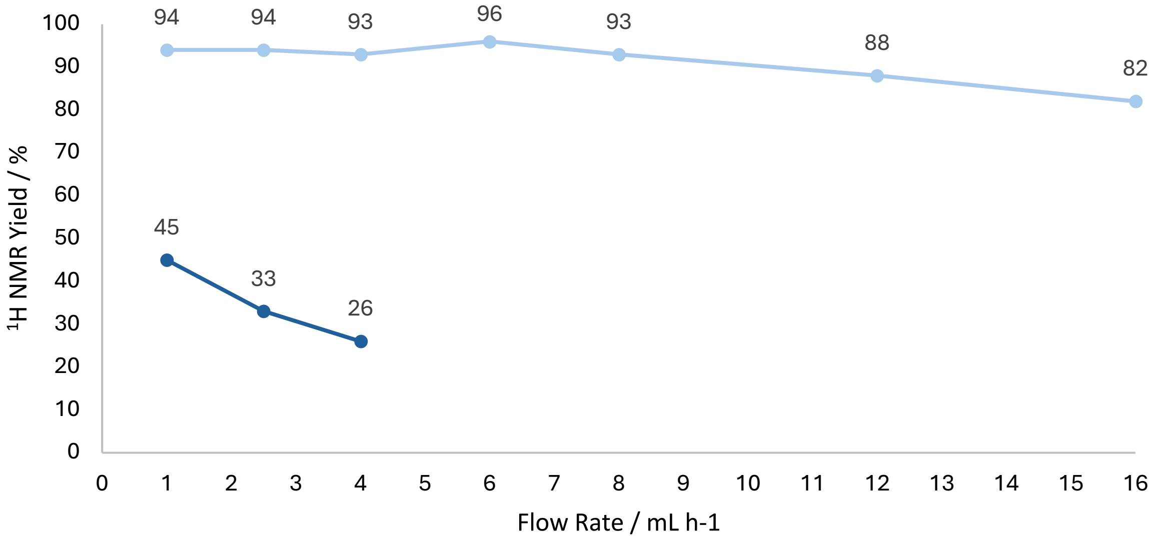

Figure S6. Effect of flow rate on reaction outcome for two substrates. Reactions were performed with a 6

Two reactions were selected to perform flow rate optimisation studies on as they achieved moderate and high yields under the batch conditions. It was observed that the formation of **4b** was influenced by flow rate with slower rates proving beneficial to reaction outcome. The higher yielding reaction was largely unaffected by flow rate, even at high flow rates (up to 16 mL h^{-1}). With these results in mind, a flow rate of 1.0 mL h^{-1} was selected as the optimal rate. Decreasing the flow rate further raised concerns about blockages when using poorly soluble substrates.

Effect of Residence Time

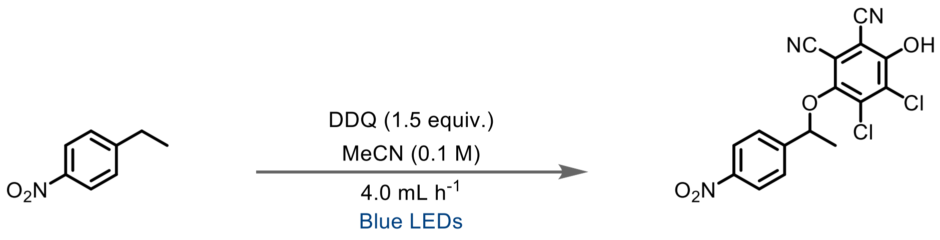

1b **4b**

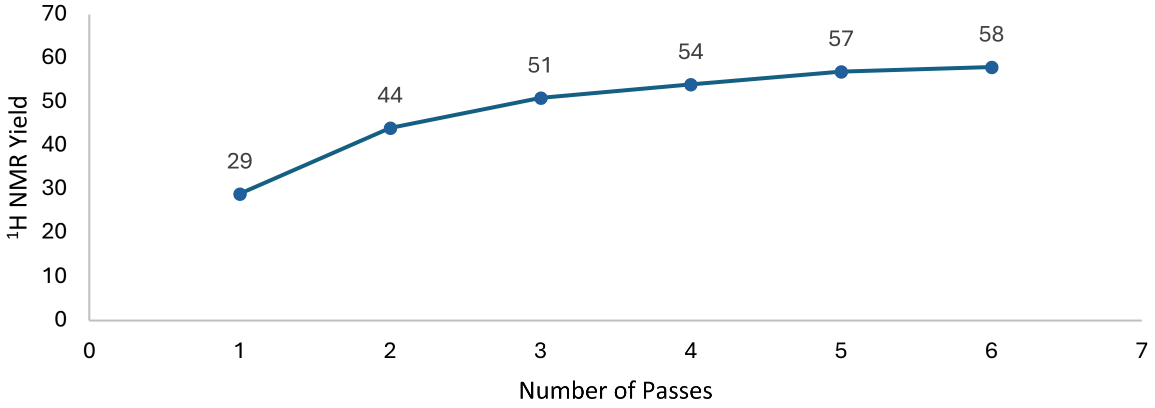

Figure S7. Effect of residence time on reaction outcome for substrate **1b**. Reaction conducted using commercially available reactor with a 2 mL flow path.[2] A higher flow rate of 4.0 mL h^{-1} was used to accelerate data collection.

Resubjecting the reaction mixture to multiple passes proved beneficial over the reduced (2 mL) flow path. Increasing the number of passes beyond three only had a minor positive impact on reaction outcome. Therefore, a flow path of 6 mL was selected for single-pass flow reactions.

Primary Benzylic Substrates

Substrate Equivalent Screening

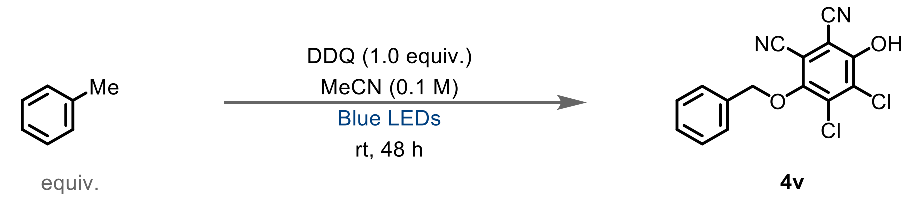

Entry	Toluene Equivalents	Yield 4v / %
1	1	24
2	3	63
3	5	68
4	7	59
5	15	53

0.4 mmol scale with respect to DDQ. Yields determined by ^{1}H NMR using mesitylene as a standard

Table S7. Effect of primary substrate loading on reaction outcome

Comparison of MeCN and DCM as Reaction Solvent

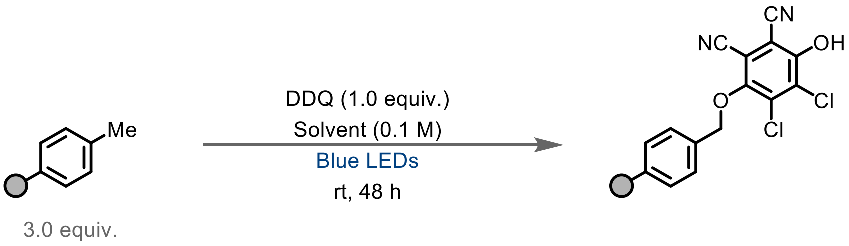

Entry		Product Yield in MeCN/ %	Product Yield in DCM/ %
1	H	68	88
2	Br	32	70
3	I	4	25
4	(4-CN)Ph	9	24
5	(4-CF_3)Ph	14	70

0.4 mmol scale with respect to DDQ. Yields determined by ^{1}H NMR using mesitylene as a standard

Table S8. Comparison between MeCN and DCM as reaction solvent for primary substrates

Further Functionalisation Screening

Nucleophilic Displacement Screening

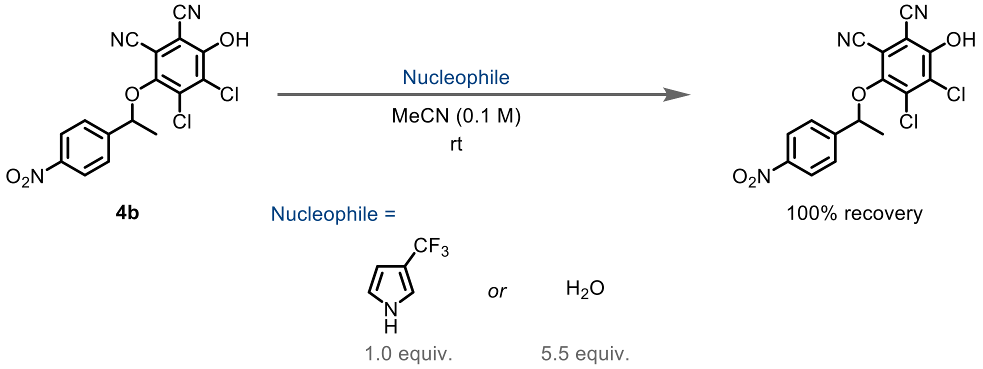

The above nucleophiles were probed with **4b** to see if they could achieve nucleophilic displacement of the DDQ-adduct. In all cases, 100% of **4b** was observed by ^{1}H NMR after the reaction.

Electrochemical Displacement Screening

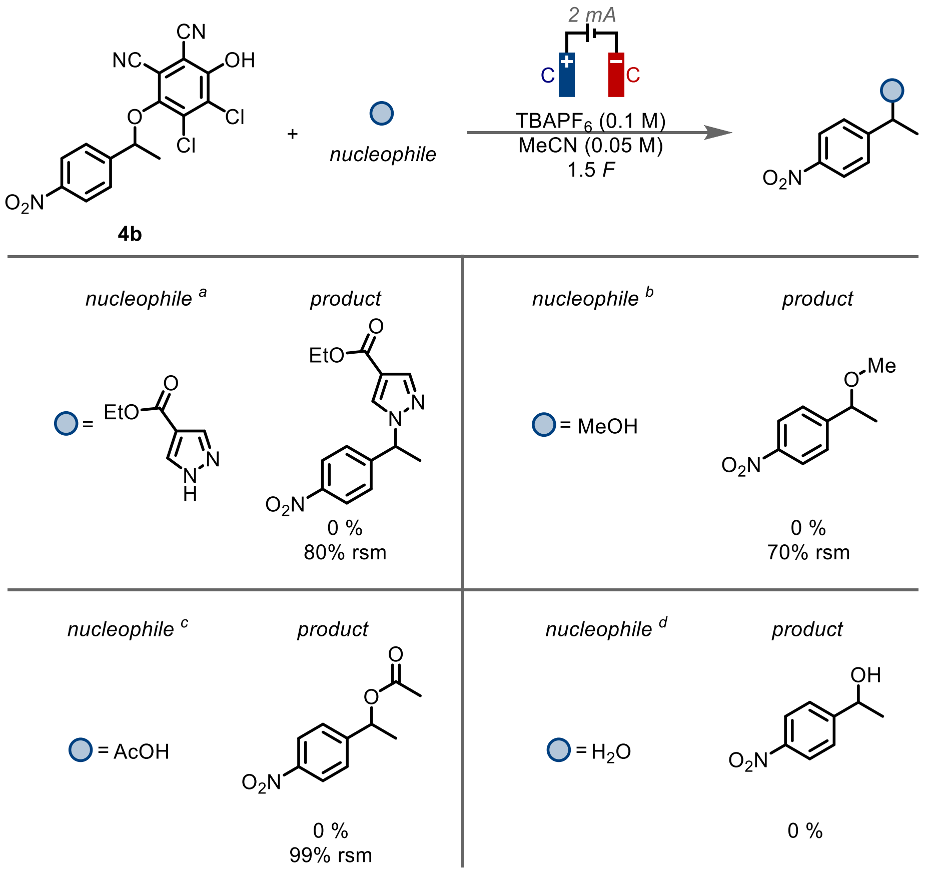

[a] 1.0 equivalents of ethyl 1 *H*-pyrazole-4-carboxylate was used. $TBABF_4$ was used as the supporting electrolyte. [b] Reaction solvent mixture was a 9:1 mixture of MeCN:MeOH. [c] A 7:1 mixture of MeCN:AcOH was used as the solvent. 10 mA current was applied. [d] Solvent used was a 9:1 mixture of MeCN:H_2O. 2 *F* of charge passed. Remaining starting material not quantified.

Figure S8. Attempts at electrochemical functionalisation of **4b**

Halogenation Screening Reactions

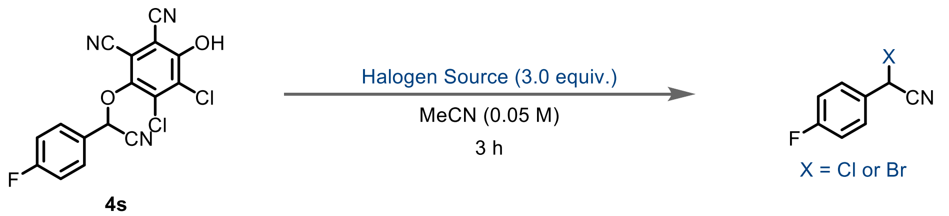

Entry	Cl Source	Br Souce	Temp / °C	Product / %
1	NCS		0	10
2	TCCA		0	52
3	DCDMH		0	92
4		NBS	60	71
5		DBDMH	60	0
6		TBCA	60	0

NCS = N-Chlorosuccinimide, TCCA = Trichloroisocyanuric acid, DCDMH = 1,3-Dichloro-5,5-dimethylhydantoin. NBS = N-bromosuccinimide, TBCA = Tribromoisocyanuric acid, DBDMH = 1,3-Dibromo-5,5-dimethylhydantoin.

Table S9 Halogen Source Screening Results

General Reaction Procedures

General Procedure 1: Batch Conditions for Secondary Benzylic Substrates

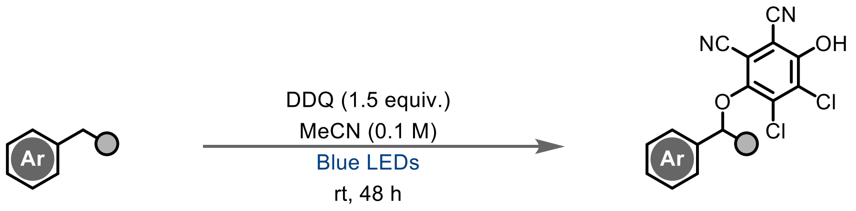

To a 7 mL microwave vial equipped with a stirrer bar was added 2,3-Dichloro-5,6-dicyano-1,4-benzoquinone (DDQ) (136 mg, 0.600 mmol, 1.50 equiv.), benzylic substrate (0.400 mmol, 1.00 equiv.) and MeCN (4.00 mL). The reaction mixture was stirred and irradiated for 48 hours with fans directed at the reaction vessels to maintain a constant temperature. The reaction mixture was then dried under reduced pressure. Mesitylene (55.6 µL, 0.400 mmol, 1.00 equiv.) and acetone-d_6 were added and the reaction mixture was analysed by NMR. The products were then purified by trituration with DCM/pentane. If further purification was required, this was done by column chromatography (solvent ramp 10-70% Et_2O in pentane Figure S9). The product containing fractions were combined and dried under reduced pressure. The product was the redissolved in EtOAc and washed with sat. aq. Na_2CO_3, and then aq. HCl (1 M). The organic layer was dried over $MgSO_4$, filtered, and dried under reduced pressure to afford purified product.

NB Variations in DDQ loading and solvent volumes are denoted in **Figure 3 of** the manuscript.

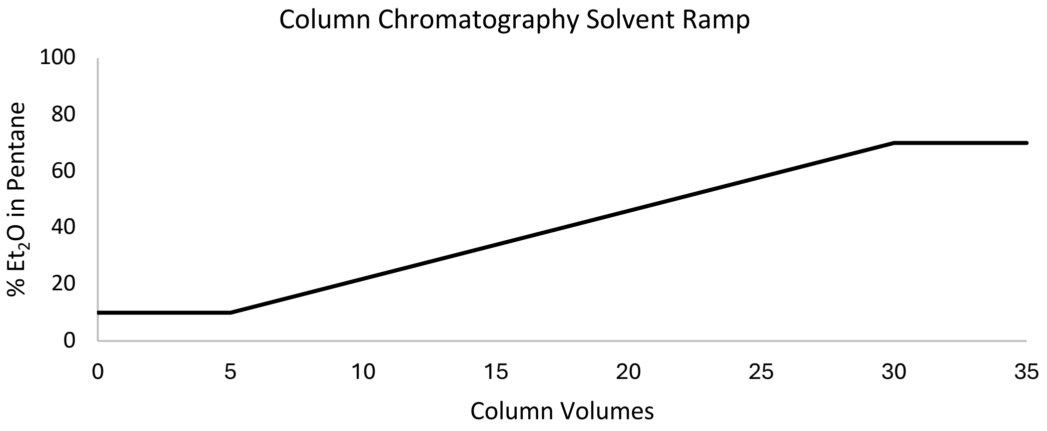

Figure S9. Column Chromatography Conditions

General Procedure 2: Batch Conditions for Primary Benzylic Substrates

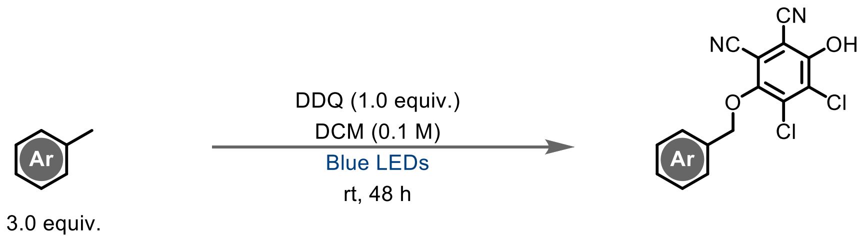

To a 7 mL microwave vial equipped with a stirrer bar was added DDQ (90.8 mg, 0.400 mmol, 1.00 equiv.), benzylic substrate (1.20 mmol, 3.00 equiv.) and DCM (4.00 mL). The reaction mixture was stirred and irradiated for 48 hours with fans directed at the reaction vessels to maintain a constant temperature. The reaction mixture was then dried under reduced pressure. Mesitylene (55.6 µL, 0.400 mmol, 1.00 equiv.) and acetone-d_6 were added and the reaction mixture was analysed by NMR. The products were then purified by trituration with DCM/pentane. If further purification was required, this was done by column chromatography (solvent ramp 10-70% Et_2O in pentane Figure S9). The product containing fractions were combined and dried under reduced pressure. The product was the redissolved in EtOAc and washed with sat. aq. Na_2CO_3, and then aq. HCl (1 M). The organic layer was dried over $MgSO_4$, filtered, and dried under reduced pressure to afford purified product.

General Procedure 3: Single Pass Flow

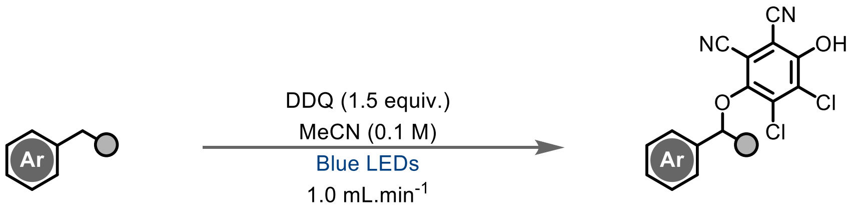

To a 50 mL round bottom flask was added DDQ (136 mg, 0.600 mmol, 1.50 equiv.) benzylic substrate (0.400 mmol, 1.00 equiv.) and MeCN (4.00 mL). The reaction mixture was taken up in a 24 mL syringe and placed on a syringe pump programmed to deliver 20 mL at a rate of 1 mL h^{-1}, allowing air to pass through the reactor after the reaction mixture. The reaction mixture was collected in a round bottom flask. Following completion of the reaction, 10 mL of MeCN was passed through the reactor. The crude mixture was dried under reduced pressure. The products were then purified by trituration with DCM/pentane. If further purification was required, this was done by column chromatography (solvent ramp 10-70% Et_2O in pentane Figure S9). The product containing fractions were combined and dried under reduced pressure. The product was the redissolved in EtOAc and washed with sat. aq. Na_2CO_3, and then aq. HCl (1 M). The organic layer was dried over $MgSO_4$, filtered, and dried under reduced pressure to afford purified product.

NB Variations in DDQ loading and solvent volumes are denoted in **Figure 3** of the manuscript.

General Procedure 4: Hydroxylation of DDQ-Adducts

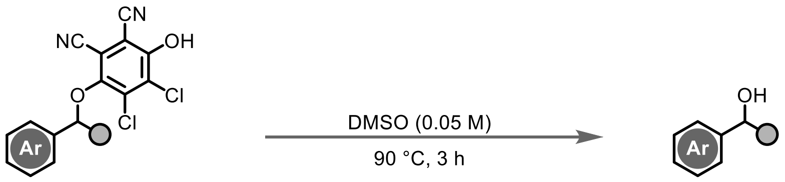

To a flame-dried 7 mL microwave vial containing a stirrer bar was added DDQ adduct (0.100 mmol, 1.00 equiv) and dry DMSO (2.00 mL). The vial was then sealed to air and stirred at 90 °C for 3 hours (unless otherwise stated). Then the solvent was removed under reduced pressure and the product isolated via column chromatography.

General Procedure 5: Halogenation of DDQ-Adducts

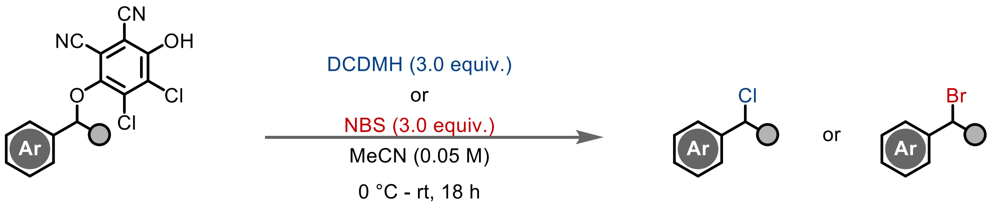

To a flame-dried 7 mL microwave vial containing a stirrer bar was added DDQ adduct (0.100 mmol, 1.00 equiv) and dry MeCN (1.00 mL). In a separate vial, 1,3-Dichloro-5,5-dimethylhydantoin (DCDMH) or *N*-bromosuccinimide (NBS) (0.300 mmol, 3.00 equiv.) was dissolved in dry MeCN (1.00 mL). Both vials were cooled to 0 °C in an ice-bath before the DCDMH/NBS solution was added dropwise to the solution containing DDQ-adduct. The vial was sealed from air and stirred for 18 hours, allowing the bath to warm to room temperature during this time. When NBS was used, the vial was heated at 60 °C. Upon completion, the solvent was removed under reduced pressure and the products isolated via column chromatography (typically 0-10% diethyl ether in pentane).

Unsuccessful Substrates

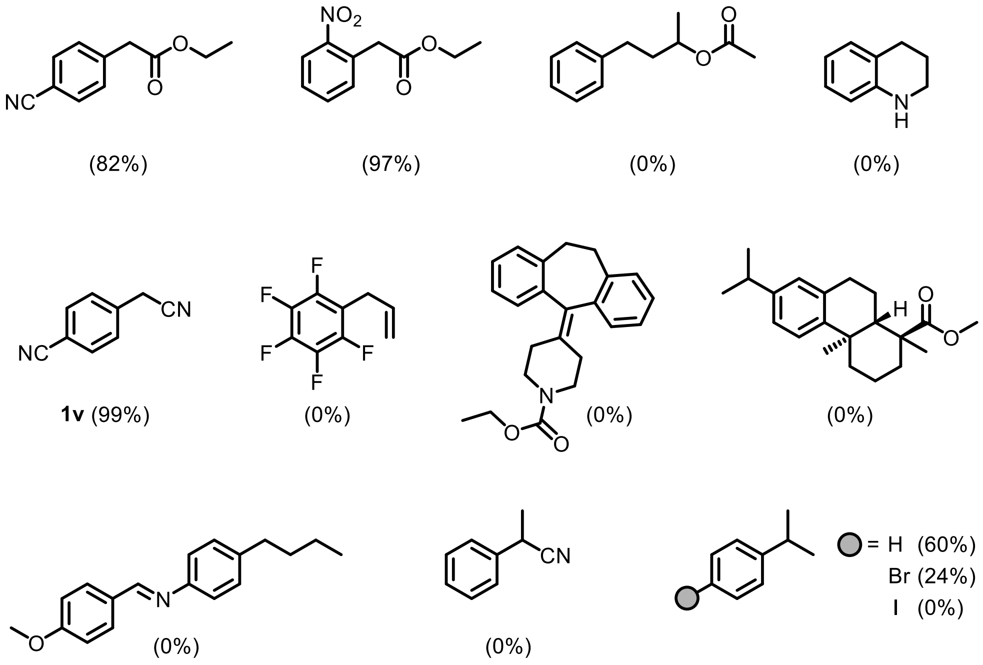

Figure S10. Unsuccessful substrates where no desired product is detected. Number in parentheses refers to remaining starting material.

Characterisation Data

Secondary Benzylic Substrates

*4,5-Dichloro-3-hydroxy-6-(1-(4-nitrophenyl)ethoxy)phthalonitrile (**4b**)*

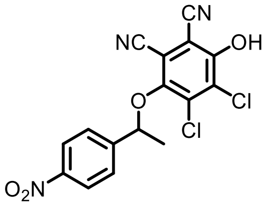

4,5-Dichloro-3-hydroxy-6-(1-(4-nitrophenyl)ethoxy)phthalonitrile was prepared from 4-nitroethylbenzene via general procedure 1 (on 0.800 mmol scale) and was purified by column chromatography to afford a light brown solid (184 mg, 61%). Isolated with trace amounts of 4,5-dichloro-3,6- dihydroxyphthalonitrile hydroquinone.

Under flow conditions (general procedure 3) this substrate achieved a 62% yield.

^{1}H NMR (400 MHz, MeCN-d_3) δ 8.23 (d, J = 8.9 Hz, 2H), 7.82 – 7.66 (m, 2H), 5.61 (q, J = 6.5 Hz, 1H), 1.70 (d, J = 6.5 Hz, 3H).

^{13}C NMR (151 MHz, MeCN-d_3) δ 154.3, 151.3, 149.1, 148.3, 129.5, 129.0, 129.0, 124.7, 114.0, 113.5, 103.0, 102.5, 84.1, 22.1.

HRMS (Nanospray-) calc: [M–H]$^-$ ($C_{16}H_8N_3O_4Cl_2$) 375.9892; measured 375.9879 = 3.5 ppm error.

IR (neat) ν_{max}/cm^{-1}: 3213 (br), 2254, 1802, 1729, 1610, 1576, 1519, 1450, 1418, 1346, 1267, 1088, 1052.

*3-(1-(4-Acetylphenyl)ethoxy)-4,5-dichloro-6-hydroxyphthalonitrile (**4c**)*

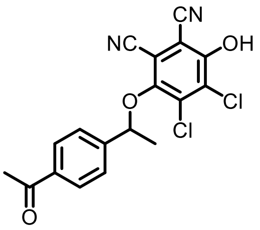

3-(1-(4-Acetylphenyl)ethoxy)-4,5-dichloro-6-hydroxyphthalonitrile was prepared from 4-ethyl-acetophenone via general procedure 1 and was purified by column chromatography to afford an off-white solid (86.0 mg, 57%). Isolated with trace amounts of 4,5-dichloro-3,6-dihydroxyphthalonitrile hydroquinone.

^{1}H NMR (400 MHz, MeCN-d_3) δ 8.02 – 7.94 (m, 2H), 7.64 – 7.57 (m, 2H), 5.58 (q, J = 6.4 Hz, 1H), 2.57 (s, 3H), 1.70 (d, J = 6.5 Hz, 3H).

^{13}C NMR (126 MHz, MeCN-d_3) δ 198.7, 154.4, 151.5, 145.9, 135.8, 129.5, 129.5, 129.1, 128.2, 114.1, 113.7, 103.1, 102.5, 84.7, 27.1, 22.0.

HRMS (Nanospray-) calc: $[M–H]^-$ ($C_{18}H_{11}N_2O_3Cl_2$) 373.0147; measured: 373.0145 = 0.5 ppm error.

IR (neat) ν_{max}/cm^{-1}: 3276 (br), 2988, 2242, 1670, 1608, 1574, 1451, 1420, 1273, 1211, 1051, 901.

*3-(1-(4-Bromophenyl)ethoxy)-4,5-dichloro-6-hydroxyphthalonitrile (**4d**)*

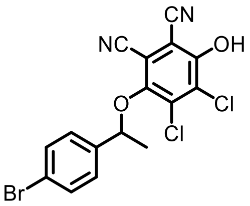

3-(1-(4-Bromophenyl)ethoxy)-4,5-dichloro-6-hydroxyphthalonitrile was prepared from 1-bromo-4-ethylbenzene via general procedure 1 to afford a beige solid (79 mg, 48 %).

Under flow conditions (general procedure 3) this substrate achieved a 65% yield.

^{1}H NMR (500 MHz, Acetone-d_6) δ 7.60 – 7.56 (m, 2H), 7.52 – 7.48 (m, 2H), 5.62 (*J* = 6.4 Hz), 1.74 (d, *J* = 6.4 Hz).

^{13}C NMR (126 MHz, Acetone-d_6) δ 154.6, 151.0, 140.4, 135.5, 132.4, 130.0, 129.3, 123.1, 113.8, 113.3, 110.8, 103.2, 84.3, 22.1.

HRMS (ESI) calc: [M-H]$^-$ ($C_{16}H_9N_2O_2BrCl_2$) 408.9152; measured 408.9160 = 1.96 ppm error.

IR (neat) ν_{max}/cm^{-1}: 2970, 1740, 1421, 1369, 1216

*3-(1-(4-Bromophenyl)butoxy)-4,5-dichloro-6-hydroxyphthalonitrile (**4e**)*

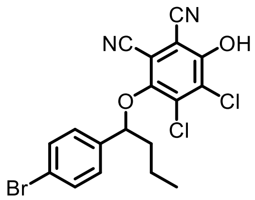

3-(1-(4-Bromophenyl)butoxy)-4,5-dichloro-6-hydroxyphthalonitrile was prepared from 1-bromo-4-butylbenzene via general procedure 1 to afford a beige solid (56 mg, 32%).

Under flow conditions (general procedure 3) this substrate achieved a 29% yield.

^{1}H NMR (500 MHz, Acetone-d_6) δ 7.56 (d, *J* = 8.0 Hz, 2H), 7.46 (d, *J* = 8.1 Hz, 2H), 5.52 (dd, *J* = 8.3, 5.8 Hz, 1H), 2.29 – 2.23 (m, 1H), 1.37 – 1.33 (m, 1H), 1.31 – 1.25 (m, 2H), 0.93 (t, *J* = 7.4 Hz, 3H).

^{13}C NMR (126 MHz, Acetone-d_6) δ 154.2, 151.4, 138.6, 135.3, 132.4, 131.0, 129.3, 123.4, 113.9, 113.3, 110.5, 103.2, 88.0, 38.2, 19.3, 14.0.

HRMS (Nanospray–) calc: [M$^-$]($C_{18}H_{12}N_2O_2Cl_2Br$) 436.9459; measured 436.9469 = 2.3 ppm error.

IR (neat) ν_{max}/cm^{-1}: 3243, 2960, 2235, 1690, 1418

*4,5-Dichloro-3-(1-(9,10-dioxo-9,10-dihydroanthracen-2-yl)ethoxy)-6-hydroxyphthalonitrile (**4f**)*

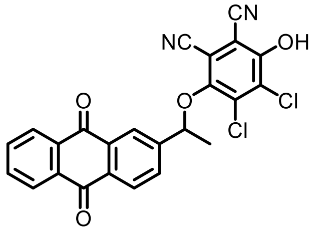

4,5-Dichloro-3-(1-(9,10-dioxo-9,10-dihydroanthracen-2-yl)ethoxy)-6-hydroxyphthalonitrile was prepared from 2-ethylanthracene-9,10-dione via general procedure 1 to afford an off-white solid (113 mg, 61%).

^{1}H NMR (500 MHz, DMSO-d_6) δ 8.33 (d, J = 1.8 Hz, 1H), 8.25 (d, J = 8.0 Hz, 1H), 8.22 (dq, J = 5.8, 3.7 Hz, 2H), 8.06 (dd, J = 8.0, 1.9 Hz, 1H), 7.97 – 7.92 (m, 2H), 5.70 (q, J = 6.4 Hz, 1H), 1.70 (d, J = 6.5 Hz, 3H).

^{13}C NMR (126 MHz, DMSO-d_6) δ 182.3, 182.2, 155.0, 148.6, 147.0, 134.7, 134.6, 134.2, 133.2, 133.1, 133.0, 132.9, 132.6, 129.4, 127.3, 126.8, 126.8, 124.7, 113.7, 113.5, 109.2, 101.9, 82.9, 21.8.

HRMS (ESI) calc: [M-H] ($C_{24}H_{11}N_2O_4Cl_2$) 461.0096; measured 461.0094, = 0.4 ppm error.

IR (neat) ν_{max}/cm^{-1}: 3399,.0, 1739, 1217, 1023

*4,5-Dichloro-3-((6-fluoro-4-oxo-1,2,3,4-tetrahydronaphthalen-1-yl)oxy)-6-hydroxyphthalonitrile (**4g**)*

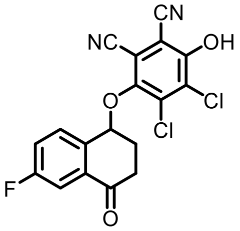

4,5-Dichloro-3-((6-fluoro-4-oxo-1,2,3,4-tetrahydronaphthalen-1-yl)oxy)-6-hydroxyphthalonitrile was prepared from 7-fluoro-3,4-dihydronaphthalen-1(2H)-one via general procedure 1 to afford an off-white solid (95 mg, 61%).

Under flow conditions (general procedure 3) this substrate achieved a 67% yield.

^{1}H NMR (500 MHz, Acetone-d_6) δ 7.69 (dd, *J* = 9.1, 2.8 Hz, 1H), 7.46 (dd, *J* = 8.5, 5.2 Hz, 1H), 7.36 (td, *J* = 8.4, 2.8 Hz, 1H), 5.72 (t, *J* = 4.1 Hz, 1H), 3.21 – 3.13 (m, 1H), 2.75 (dtd, *J* = 10.2, 9.0, 4.7 Hz, 2H), 2.63 (ddt, *J* = 12.1, 10.6, 3.4 Hz, 1H).

^{13}C NMR (126 MHz, Acetone-d_6) δ 195.5 (d, *J* = 1.4 Hz), 164.4 (d, *J* = 248.5 Hz), 155.0, 150.3, 136.0 (d, *J* = 3.3 Hz), 135.9 , 135.6 (d, *J* = 6.3 Hz), 132.9 (d, *J* = 7.7 Hz), 129.4, 121.2 (d, *J* = 22.3 Hz), 114.0 (d, *J* = 22.5 Hz), 113.3, 111.4, 110.5, 102.4, 81.0, 33.9, 29.5.

^{19}F NMR (377 MHz, Acetone-d_6) δ -111.6 (td, *J* = 8.8, 5.2 Hz).

HRMS (Nanospray) calc: $[M–H]^-$ ($C_{18}H_8N_2O_4Cl_2F$)388.9896; Measured: 388.9897 = 0.3 ppm error.

IR (neat) $ν_{max}$/cm^{-1}: 2970, 2236, 1689, 1420, 1220, 907

*3-(1-(3-(tert-Butyl)phenyl)ethoxy)-4,5-dichloro-6-hydroxyphthalonitrile (**4h**)*

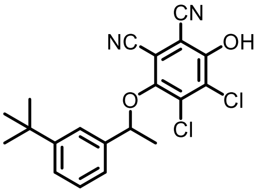

3-(1-(3-(tert-Butyl)phenyl)ethoxy)-4,5-dichloro-6-hydroxyphthalonitrile was prepared from 1-(tert-butyl)-3-ethylbenzene via general procedure 1 to afford a beige solid (62 mg, 40%).

Under flow conditions (general procedure 3) this substrate achieved a 35% yield.

^{1}H NMR (500 MHz, Acetone-d_6) δ 7.52 (q, J = 1.3 Hz, 1H), 7.40 (dtd, J = 6.0, 3.7, 1.8 Hz, 1H), 7.33 – 7.30 (m, 2H, C2-H), 5.69 (q, J = 6.5 Hz, 1H), 1.79 (d, J = 6.4 Hz, 3H), 1.29 (s, 9H).

^{13}C NMR (126 MHz, Acetone-d_6) δ 154.2, 152.1, 151.5, 139.9, 135.7, 129.3, 129.1, 126.7, 125.5, 125.3, 113.9, 113.3, 111.0, 103.1, 85.4, 35.3, 31.5, 21.8.

HRMS (Nanospray) calc: [M^{-}]($C_{20}H_{17}N_2O_2Cl_2$) 387.0667; measured 387.0668 = 0.3 ppm error.

IR (neat) ν_{max}/cm^{-1}: 3251,.1, 2236, 2073, 706.

*4,5-Dichloro-3-hydroxy-6-((1-phenylbut-3-yn-1-yl)oxy)phthalonitrile (**4i**)*

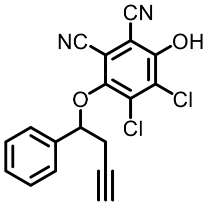

4,5-Dichloro-3-hydroxy-6-((1-phenylbut-3-yn-1-yl)oxy)phthalonitrile was prepared from but-3-yn-1-ylbenzene via general procedure 1 to afford a dark brown solid (87 mg,61%).

^{1}H NMR (500 MHz, Acetone-d_6) δ 7.59 – 7.55 (m, 2H), 7.43 – 7.38 (m, 3H), 5.67 (dd, *J* = 7.7, 6.0 Hz, 1H), 3.20 – 3.02 (m, 2H), 2.40 (t, *J* = 2.7 Hz, 1H).

^{13}C NMR (126 MHz, Acetone-d_6) δ 154.5, 151.0, 138.2, 135.3, 130.1, 129.3, 129.2, 128.8, 113.8, 113.2, 110.7, 103.2, 86.1, 79.9, 73.0, 26.5.

HRMS (Nanospray) calc: [M$^-$] ($C_{18}H_9N_2O_2Cl_2$) 355.0041; measured 355.004 = 0.8 ppm error

IR (neat) ν_{max}/cm^{-1}: 3292, 2235, 1698,1417, 1203

*4,5-Dichloro-3-(1-(4'-fluoro-[1,1'-biphenyl]-4-yl)ethoxy)-6-hydroxyphthalonitrile (**4j**)*

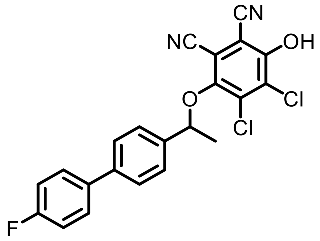

4,5-Dichloro-3-(1-(4'-fluoro-[1,1'-biphenyl]-4-yl)ethoxy)-6-hydroxyphthalonitrile was prepared from 4-ethyl-4'-fluoro-1,1'-biphenyl via general procedure 1 to afford an off-white solid (91 mg, 53%).

^{1}H NMR (500 MHz, Acetone-d_6) δ 7.72 – 7.69 (m, 2H), 7.68 – 7.61 (m, 4H), 7.22 (t, *J* = 8.7 Hz, 2H, C25-H), 5.70 (q, *J* = 6.5 Hz, 1H), 1.77 (d, *J* = 6.5 Hz, 3H).

^{13}C NMR (126 MHz, Acetone-d_6) δ 163.4 (d, *J* = 244.9 Hz), 154.4, 151.3, 141.1, 140.1, 137.6 (d, *J* = 3.2 Hz), 135.6, 129.7 (d, *J* = 8.2 Hz), 129.3, 128.6, 127.7, 116.4 (d, *J* = 21.7 Hz), 113.9, 113.4, 110.9, 103.3, 84.7, 22.1.

^{19}F NMR (471 MHz, Acetone-d_6) δ -116.92 (tt, *J* = 9.1, 5.3 Hz).

HRMS (ESI) calc: $[M+Na]^+$ ($C_{22}H_{13}N_2O_4Cl_2F$) 449.0230; Measured: 449.0233 = 0.67 ppm error.

IR (neat) ν_{max}/cm^{-1}: 3275, 2239, 1739, 1602, 1498.

*3-(Benzhydryloxy)-4,5-dichloro-6-hydroxyphthalonitrile (**4k**)*

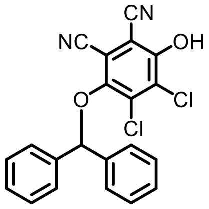

3-(Benzhydryloxy)-4,5-dichloro-6-hydroxyphthalonitrile was prepared from diphenylmethane via general procedure 1 to afford an off-white solid (74 mg, 47%). Isolated with trace amounts of 4,5-dichloro-3,6-dihydroxyphthalonitrile hydroquinone.

^{1}H NMR (600 MHz, Acetone-d_6) δ 7.53 – 7.50 (m, 4H), 7.40 – 7.37 (m, 4H), 7.35 (dd, *J* = 7.2, 6.9 Hz, 2H), 6.72 (s, 1H).

^{13}C NMR (151 MHz, Acetone-d_6) δ 154.4, 150.8, 139.9, 135.5, 129.7, 129.4, 129.0, 128.7, 113.6, 113.2, 110.8, 103.1, 88.7.

HRMS (Nanospray) calc: $[M\text{-}H]^-$ ($C_{21}H_{11}N_2O_2Cl_2$) 393.0198; Measured 393.0193 = 1.3 ppm error.

IR (neat) ν_{max}/cm^{-1}: 3209, 2251,1693, 1450, 1185, 885

*Ethyl 2-(4-(1-(2,3-dichloro-5,6-dicyano-4-hydroxyphenoxy)-2-methylpropyl)phenyl)propanoate (**4l**)*

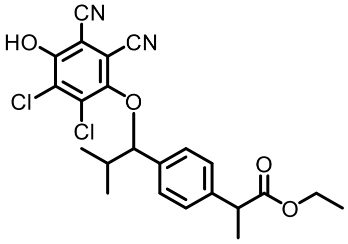

Ethyl 2-(4-(1-(2,3-dichloro-5,6-dicyano-4-hydroxyphenoxy)-2-methylpropyl)phenyl)propanoate was prepared from ibuprofen ethyl ester via general procedure 1 and was purified by column chromatography (0-70% EtOAc in pentane) to afford an off-white solid (88.1 mg, 48 %). Isolated with trace amounts of 4,5-dichloro-3,6-dihydroxyphthalonitrile hydroquinone.

^{1}H NMR (400 MHz, MeCN-d_3) δ 7.31 – 7.27 (m, 2H), 7.25 – 7.21 (m, 2H), 5.30 (d, *J* = 8.3 Hz, 1H), 4.09 – 4.00 (m, 2H), 3.70 (qd, *J* = 7.3, 2.1 Hz, 1H), 2.54 – 2.43 (m, 1H), 1.38 (d, *J* = 7.1 Hz, 3H), 1.23 (d, *J* = 6.6 Hz, 3H), 1.11 (t, *J* = 7.1 Hz, 3H), 0.79 (d, *J* = 6.8 Hz, 3H).

^{13}C NMR (126 MHz, MeCN-d_3) δ 175.0, 153.6, 151.8, 142.9*, 136.2, 130.0*, 129.3, 129.1, 128.3, 114.3, 113.6, 102.8, 102.5, 93.2*, 61.5, 45.8*, 34.3*, 19.8, 18.9, 18.8*, 14.4.

Peaks marked * are present as two peaks, as this compound was synthesised as a mixture of diastereomers.

HRMS (Nanospray-) calc: $[M–H]^–$ ($C_{23}H_{21}N_2O_4Cl_2$) 459.0878; measured 459.0972 = 1.3 ppm error.

IR (neat) ν_{max}/cm^{-1}:3241 (br), 2976, 2250, 1716, 1574, 1481, 1275, 1204, 1075, 890.

*(2R)-4-(2,3-Dichloro-5,6-dicyano-4-hydroxyphenoxy)-4-phenylbutan-2-yl 2,4,6-triisopropylbenzoate (**4m**)*

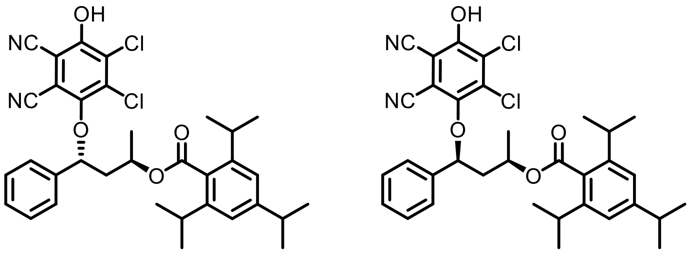

anti (major) syn (minor)

(2*R*)-4-(2,3-Dichloro-5,6-dicyano-4-hydroxyphenoxy)-4-phenylbutan-2-yl-2,4,6-triisopropylbenzoate was prepared from (R)-4-phenylbutan-2-yl 2,4,6-triisopropylbenzoate via general procedure 1 to afford a beige solid (131 mg, 54%) with a diastereotopic excess 1:1.15 syn:anti. Isolated with trace amounts of 4,5-dichloro-3,6-dihydroxyphthalonitrile hydroquinone.

^{1}H NMR (600 MHz, Acetone-d_6)

Major δ 7.50 (ddq, *J* = 7.2, 4.6, 2.5 Hz, 2H), 7.44 – 7.37 (m, 3H), 7.11 (s, 2H), 5.67 (dd, *J* = 7.8, 5.9 Hz, 1H), 5.33 (h, *J* = 6.4 Hz, 1H), 2.95 – 2.89 (m, 2H), 2.87 (q, *J* = 6.9 Hz, 1H), 2.70 (ddd, *J* = 13.8, 7.8, 5.8 Hz, 1H), 2.59 (dd, *J* = 13.5, 6.7 Hz, 1H), 1.45 (d, *J* = 6.5 Hz, 3H), 1.27-1.21 (m, 18H).

Minor δ 7.50 (ddq, *J* = 7.2, 4.6, 2.5 Hz, 2H), 7.45 – 7.35 (m, 3H), 7.11 (s, 2H), 5.64 (dd, *J* = 7.8, 5.9 Hz, 1H), 5.06 – 5.01 (m, 1H), 2.95 – 2.89 (m, 2H), 2.87 (q, *J* = 6.9 Hz, 1H2.81 (ddd, *J* = 14.3, 8.5, 5.9 Hz, 1H), 2.56 – 2.52 (m, 1H), 1.44 (d, *J* = 6.3 Hz, 3H), 1.27-1.21 (m, 18H).

^{13}C NMR (151 MHz, Acetone-d_6)

Major δ 170.4, 154.3, 151.0, 150.8, 145.4, 137.9, 135.0, 131.6, 130.3, 129.5, 129.1, 128.9, 121.5, 113.7, 113.1, 110.6, 103.0, 85.2, 69.6, 43.1, 35.1, 32.1, 24.6, 24.2, 24.2, 20.4.

Minor δ 170.3, 154.4, 150.9, 150.9, 145.4, 137.5, 135.2, 131.6, 130.5, 129.6, 129.1, 129.0, 121.5, 113.8, 113.1, 110.8, 103.0, 85.9, 69.7, 42.1, 35.1, 32.0, 24.5, 24.34, 24.2, 20.6.

HRMS (Nanospray) calc: $[M-H]^-$ ($C_{34}H_{35}N_2O_4Cl_2$) 605.1974; Measured: 605.1983 = 1.5 ppm error.

IR (neat) ν_{max}/cm^{-1}: 3228, 2961, 2254, 1721, 1273.

*4,5-Dichloro-3-((5-cyano-1-phenyl-3-(4,4,5,5-tetramethyl-1,3,2-dioxaborolan-2-yl)pentyl)oxy)-6-hydroxyphthalonitrile (**4n**)*

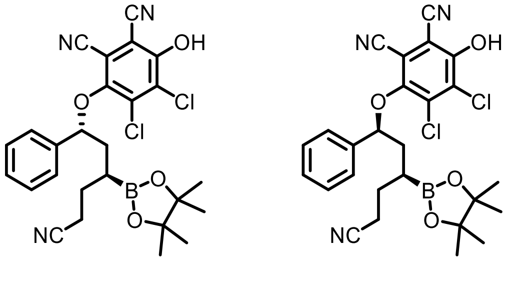

anti (major) syn (minor)

4,5-Dichloro-3-((5-cyano-1-phenyl-3-(4,4,5,5-tetramethyl-1,3,2-dioxaborolan-2-yl)pentyl)oxy)-6-hydroxyphthalonitrile was prepared from 6-phenyl-4-(4,4,5,5-tetramethyl-1,3,2-dioxaborolan-2-yl)hexanenitrile via general procedure 1 to afford a light brown solid (110 mg, 52%) with diastereotopic excess (1:1.20) syn:anti. Isolated with trace amounts of 4,5-dichloro-3,6-dihydroxyphthalonitrile hydroquinone.

^{1}H NMR (500 MHz, Acetone-d_6)

Major δ 7.49 – 7.46 (m, 2H), 7.39 – 7.32 (m, 3H), 5.78– 5.75 (m, 1H), 2.51 – 2.43 (m, 2H), 2.42 – 2.34 (m, 1H), 2.27 – 2.21 (m, 1H), 1.83 – 1.78 (m, 2H), 1.26 (dd, *J* = 11.9, 3.8 Hz, 12H), 0.95-0.89 (m, 1H)

Minor δ 7.49 – 7.46 (m, 2H), 7.39 – 7.32 (m, 3H), 5.78– 5.75 (m, 1H), 2.56 (ddd, *J* = 16.6, 8.2, 6.3 Hz, 1H), 2.51 – 2.33 (m, 1H), 2.42 – 2.34 (m, 1H), 2.20 – 2.15 (m, 1H), 1.78 – 1.71 (m, 2H), 1.26 (dd, *J* = 11.9, 3.8 Hz, 12H), 0.95-0.89 (m, 1H).

^{13}C NMR (126 MHz, Acetone-d_6)

Major δ 153.9, 151.3, 138.8, 135.1, 130.0, 129.2, 129.1, 128.9, 120.5, 113.9, 113.2, 110.1, 103.0, 87.7, 84.3, 37.5, 27.5, 25.2, 16.5.

Minor δ 154.0, 151.0, 138.4, 135.4, 130.0, 129.2, 129.1, 129.0, 120.4, 113.8, 113.2, 110.5, 103.0, 87.7, 84.3, 36.8, 28.2, 25.1, 16.4.

HRMS (Nanospray) calc: $[M-H]^-$ ($C_{26}H_{25}N_3O_4Cl_2B$) 524.1315; Measured: 524.1326 = 2.1 ppm error.

IR (neat) ν_{max}/cm^{-1}: 3205, 2979, 2253, 1694, 1450

*Ethyl 2-(4-bromophenyl)-2-(2,3-dichloro-5,6-dicyano-4-hydroxyphenoxy)acetate (**4o**)*

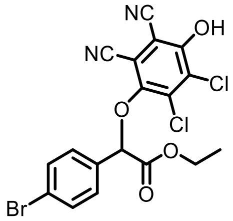

Ethyl 2-(4-bromophenyl)-2-(2,3-dichloro-5,6-dicyano-4-hydroxyphenoxy)acetate was prepared from ethyl 2-(4-bromophenyl)acetate via general procedure 1 to afford a brown solid (130 mg, 69%).

^{1}H NMR (500 MHz, Acetone-d_6) δ 7.67 – 7.63 (m, 2H), 7.60 – 7.57 (m, 2H), 6.06 (s, 1H), 4.27 – 4.15 (m, 2H), 1.20 (t, *J* = 7.1 Hz, 3H).

^{13}C NMR (101 MHz, Acetone-d_6) δ 168.3, 154.5, 150.7, 134.7, 134.7, 132.8, 131.1, 129.0, 124.3, 113.4, 113.1, 110.6, 103.3, 83.6, 62.6, 14.2.

HRMS (ESI) calc: [M-H^{-}]($C_{18}H_{11}N_2O_4Cl_2Br$) 466.9196; measured 466.9187 = 1.93 ppm error.

IR (neat) ν_{max}/cm^{-1}: 3224, 2981, 2237, 1748, 779

*Ethyl 2-(2,3-dichloro-5,6-dicyano-4-hydroxyphenoxy)-2-phenylacetate (**4p**)*

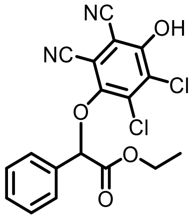

Ethyl 2-(2,3-dichloro-5,6-dicyano-4-hydroxyphenoxy)-2-phenylacetate was prepared from ethyl 2-phenylacetate via general procedure 1 to afford a brown solid (136 mg, 87%).

Under flow conditions (general procedure 3) this substrate achieved a 93% yield at 0.400 mmol scale. At 4.00 mmol a yield of 99% was achieved. At 20.0 mmol scale a yield of 83% was achieved.

^{1}H NMR (400 MHz, Acetone-*d*$_6$**)** δ 7.62 (dd, *J* = 6.6, 3.0 Hz, 2H), 7.45 (q, *J* = 3.5 Hz, 3), 6.09 (s, 1), 4.20 (p, *J* = 7.0 Hz, 2H), 1.18 (t, *J* = 7.1 Hz, 3H).

^{13}C NMR (101 MHz, Acetone-*d*$_6$**)** δ 168.7, 154.5, 150.7, 135.2, 134.7, 130.6, 129.6, 129.2 129.1, 113.4, 113.2, 110.5, 103.2, 84.2, 62.4, 14.2.

HRMS (Nanospray) calc: [M-H]$^{+}$($C_{18}H_{11}N_2O_4Cl_2$) 389.0096; measured 389.0089 = -1.8 ppm error.

IR (neat) ν_{max}/cm^{-1}: 2979, 1738, 1419, 1216, 1024

*Ethyl 2-(2,3-dichloro-5,6-dicyano-4-hydroxyphenoxy)-2-(4-fluorophenyl)acetate (**4q**)*

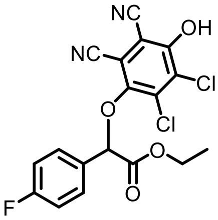

Ethyl 2-(2,3-dichloro-5,6-dicyano-4-hydroxyphenoxy)-2-(4-fluorophenyl)acetate was prepared from ethyl 2-(4-fluorophenyl)acetate via general procedure 1 to afford an off-white solid (118 mg, 72%).

Under flow conditions (general procedure 3) this substrate achieved an 83% yield.

^{1}H NMR (400 MHz, Acetone-*d*$_6$) δ 7.70 – 7.65 (m, 2H), 7.25 – 7.19 (m, 2H), 6.07 (s, 1H), 4.21 (qd, *J* = 7.1, 4.7 Hz, 2H), 1.19 (t, *J* = 7.1 Hz, 3H).

^{13}C NMR (101 MHz, Acetone-*d*$_6$) δ 168.5, 164.3 (d, *J* = 247.3 Hz), 154.5, 150.7, 134.8, 131.5 (d, *J* = 8.7 Hz), 131.5, 129.0, 116.5 (d, *J* = 21.9 Hz), 113.4, 113.1, 110.6, 103.2, 83.6, 62.5, 14.2.

^{19}F NMR (377 MHz, Acetone-*d*$_6$) δ -112.8 (m, 1F).

HRMS (ESI) calc: [M-H]$^-$ ($C_{18}H_{11}N_2O_4FCl_2$) 406.9996; Measured: 406.9991 = 1.2 ppm error.

IR (neat) ν_{max}/cm^{-1}: 3636, 3231, 2950, 2526, 2237, 842

*Butyl 2-(2,3-dichloro-5,6-dicyano-4-hydroxyphenoxy)-2-(4-fluorophenyl)acetate (**4r**)*

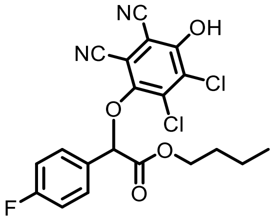

Butyl 2-(2,3-dichloro-5,6-dicyano-4-hydroxyphenoxy)-2-(4-fluorophenyl)acetate)was prepared from butyl 2-(4-fluorophenyl)acetate via general procedure 1 to afford a beige solid (140 mg, 80%).

^{1}H NMR (400 MHz, Acetone-d_6) δ 7.71 – 7.66 (m, 2H), 7.25 – 7.19 (m, 2H), 6.10 (s, 1H), 4.16 (td, *J* = 6.5, 0.8 Hz, 2H), 1.58 – 1.51 (m, 2H), 1.27 – 1.21 (m, 2H), 0.83 (t, *J* = 7.4 Hz, 3H).

^{13}C NMR (101 MHz, Acetone-d_6) δ 168.6, 164.2 (d, *J* = 247.3 Hz), 154.5, 150.6, 134.7, 131.6 (d, *J* = 3.2 Hz), 131.4 (d, *J* = 8.7 Hz), 129.0, 116.5 (d, *J* = 22.0 Hz), 113.4, 113.1, 110.6, 103.2, 83.5, 66.2, 31.1, 19.5, 13.8.

^{19}F NMR (377 MHz, Acetone-d_6) δ -112.7 (m, 1F).

HRMS (Nanospray) calc: [M-H]$^-$ ($C_{20}H_{14}N_2O_4Cl_2F$) 435.0315; Measured: 435.0307 = -1.8 ppm error.

IR (neat) ν_{max}/cm^{-1}: 3697, 3227, 2966, 2238, 1750

*4,5-Dichloro-3-(cyano(4-fluorophenyl)methoxy)-6-hydroxyphthalonitrile (**4s**)*

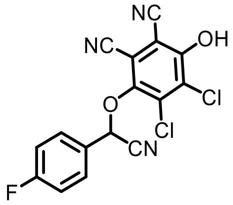

4,5-Dichloro-3-(cyano(4-fluorophenyl)methoxy)-6-hydroxyphthalonitrile was prepared from 2-(4-fluorophenyl)acetonitrile via general procedure 1 to afford an off-white solid (142 mg, 98%).

^{1}H NMR (500 MHz, Acetone-*d*$_6$) δ 7.80 (dd, *J* = 8.5, 5.0 Hz, 2H), 7.31 (t, *J* = 8.5 Hz, 2H), 6.41 (s, 1H).

^{13}C NMR (126 MHz, Acetone-*d*$_6$) δ 164.9 (d, *J* = 249.3 Hz), 155.8, 148.6, 135.6, 131.9 (d, *J* = 9.0 Hz), 129.4, 129.0 (d, *J* = 3.3 Hz), 117.2 (d, J = 22.4 Hz), 116.5, 112.9, 112.9, 111.6, 103.4, 73.7.

^{19}F NMR (376 MHz, Acetone-*d*$_6$) δ -110.7 (tt, *J* = 8.7, 5.2 H).

HRMS (Nanospray) calc: [M^{-}]($C_{16}H_5N_3O_2Cl_2F$) 359.9743; measured 359.9741 = -0.6 ppm error.

IR (neat) ν_{max}/cm^{-1}: 2700, 1736, 1011, 1421, 1367

*4,5-Dichloro-3-(cyano(phenyl)methoxy)-6-hydroxyphthalonitrile (**4t**)*

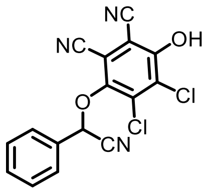

4,5-Dichloro-3-(cyano(phenyl)methoxy)-6-hydroxyphthalonitrile was prepared from 2-phenylacetonitrile via general procedure 1 to afford an off-white solid (135 mg, 98%).

^{1}H NMR (600 MHz, Acetone-d_6) δ 7.79 – 7.78 (m, 2H), 7.60 – 7.58 (m, 3H), 6.47 (s, 1H)

^{13}C NMR (151 MHz, Acetone-d_6) δ 155.9, 148.8, 135.6, 132.8, 131.9, 130.3, 129.4, 129.4, 116.8, 113.0, 113.0, 111.7, 103.5, 74.6.

HRMS (Nanospray) calc: $[M\text{-}H]^+$($C_{16}H_7N_3O_2Cl_2$) 341.9837; measured 341.9848 = 3.2 ppm error

IR (neat) ν_{max}/cm^{-1}: 2970, 1739, 1421, 1367, 1217

4,5-Dichloro-3-hydroxy-6-(2-oxo-1,2-diphenylethoxy)phthalonitrile (**4u**)

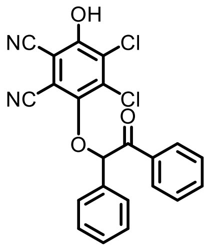

4,5-Dichloro-3-hydroxy-6-(2-oxo-1,2-diphenylethoxy)phthalonitrile was prepared from 1,2-diphenylethan-1-one via general procedure 1 to afford an off-white solid (88 mg, 52%). Isolated with trace amounts of 4,5-dichloro-3,6-dihydroxyphthalonitrile hydroquinone.

^{1}H NMR (500 MHz, Acetone-d_6) δ 8.05 – 8.03 (m, 2H), 7.68 – 7.64 (m, 2H), 7.68 – 7.57 (m, 1H), 7.47 (t, J = 7.8 Hz, 2H), 7.43 – 7.39 (m, 3H), 7.22 (s, 1H).

^{13}C NMR (126 MHz, Acetone-d_6) δ 193.4, 154.2, 151.3, 135.2, 134.9, 134.6, 131.3, 130.8, 130.2, 130.0, 129.7, 129.6, 129.0, 113.6, 113.5, 110.6, 103.2, 87.4.

Data are in agreement with the literature.[4]

Primary Benzylic Substrates

3-(Benzyloxy)-4,5-dichloro-6-hydroxyphthalonitrile (**4v**)

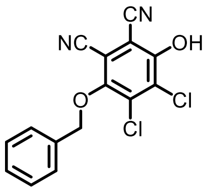

3-(Benzyloxy)-4,5-dichloro-6-hydroxyphthalonitrile was prepared from toluene via general procedure 2 to afford a cream solid (112 mg, 88%).

^{1}H NMR (400 MHz, DMSO-d_6) δ 7.51 – 7.48 (m, 2H), 7.46 – 7.39 (m, 2H), 5.12 (s, 2H).

^{13}C NMR (101 MHz, DMSO-d_6) δ 155.2, 150.8, 135.5, 134.7, 129.6, 129.4, 129.4, 129.1, 113.9, 113.6, 109.5, 102.5, 77.5.

Data are in agreement with the literature.[5]

*4,5-Dichloro-3-((4-fluorobenzyl)oxy)-6-hydroxyphthalonitrile (**4w**)*

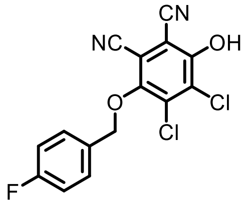

4,5-Dichloro-3-((4-fluorobenzyl)oxy)-6-hydroxyphthalonitrile was prepared from 4-fluorotoluene via general procedure 2 to afford a beige solid (94 mg, 70%).

^{1}H NMR (500 MHz, Acetone-d_6) δ 7.65 – 7.61 (m, 2H), 7.23 – 7.18 (m, 2H, C1-H), 5.24 (s, 2H).

^{13}C NMR (126 MHz, Acetone-d_6) δ 164.0 (d, *J* = 245.7 Hz), 154.7, 152.2, 135.5, 132.4 (d, *J* = 3.2 Hz), 132.4 (d, *J* = 8.7 Hz), 129.1, 116.3 (d, *J* = 21.8 Hz), 113.5, 113.24, 110.8, 103.2, 77.4.

^{19}F NMR (471 MHz, Acetone-d_6) δ -114.2 (tt, *J* = 9.1, 5.4 Hz)

HRMS (Nanospray) calc: $[M-H]^-$ ($C_{15}H_6N_2O_2FCl_2$) 334.9790; Measured: 334.9791 = 0.3 ppm error.

IR (neat) ν_{max}/cm^{-1}: 3696, 3291, 2922, 2238, 1511, 1033

Data are in agreement with the literature.[5] Previously reported spectra are in DMSO-d_6 therefore we have included copies of our spectra recorded in acetone-d_6.

*3-((4-Bromobenzyl)oxy)-4,5-dichloro-6-hydroxyphthalonitrile (**4x**)*

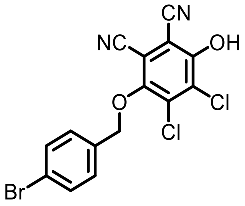

3-((4-Bromobenzyl)oxy)-4,5-dichloro-6-hydroxyphthalonitrile was prepared from 4-bromotoluene via general procedure 2 to afford a beige solid (111 mg, 70%).

^{1}H NMR (400 MHz, Acetone-d_6) δ 7.65 – 7.61 (m, 2H), 7.56 – 7.50 (m, 2H), 5.24 (s, 2H)

^{13}C NMR (101 MHz, Acetone-d_6) δ 154.9, 152.1, 135.6, 135.5, 132.6, 131.9, 129.2, 123.4, 113.5, 113.3, 110.6, 103.2, 77.3

HRMS (Nanospray) calc: $[M-H]^-$ ($C_{15}H_6N_2O_2Cl_2Br$) 394.8990; Measured: 394,8988 = -0.5 ppm error.

IR (neat) ν_{max}/cm^{-1}: 3696, 3282, 2945, 2238, 1447, 1033

*4,5-Dichloro-3-hydroxy-6-((4-iodobenzyl)oxy)phthalonitrile (**4i**)*

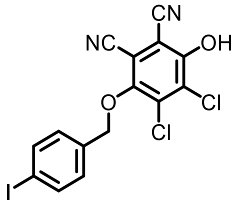

4,5-Dichloro-3-hydroxy-6-((4-iodobenzyl)oxy)phthalonitrile was prepared from 4-iodotoluene via general procedure 2 to afford a cream solid (45 mg, 25%).

^{1}H NMR (500 MHz, Acetone-d_6) δ 7.85 – 7.82 (m, 2H), 7.42 – 7.39 (m, 2H), 5.23 (s, 2H)

^{13}C NMR (126 MHz, Acetone-d_6) δ 154.8, 152.2, 138.6, 136.1, 135.5, 131.9, 129.2, 113.5, 113.2, 110.7, 103.2, 95.2, 77.4.

HRMS (Nanospray) calc: $[M-H]^-$ ($C_{15}H_6N_2O_2Cl_2I$) 442.8851; Measured: 442.8867 = 3.6 ppm error.

IR (neat) ν_{max}/cm^{-1}: 3703, 3297, 2950, 2236, 1443, 1056

*3-((3-Bromobenzyl)oxy)-4,5-dichloro-6-hydroxyphthalonitrile (**4z**)*

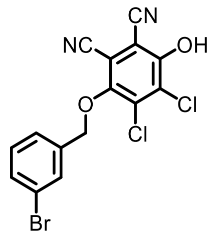

3-((3-Bromobenzyl)oxy)-4,5-dichloro-6-hydroxyphthalonitrile was prepared from 3-bromotoluene via general procedure 2 to afford a cream solid (119 mg, 75%).

^{1}H NMR (400 MHz, Acetone-d_6) δ 7.78 (t, J = 1.9 Hz, 1H), 7.60 – 7.57 (m, 2H), 7.40 (t, J = 7.8 Hz, 1H), 5.25 (s, 2H).

^{13}C NMR (101 MHz, Acetone-d_6) δ 154.8, 152.0, 138.8, 135.4, 132.6, 132.5, 131.4, 129.2, 128.6, 122.9, 113.4, 113.2, 110.6, 103.1, 77.1.

HRMS (Nanospray) calc: $[M-H]^-$ ($C_{15}H_6N_2O_2Cl_2Br$) 394.8990; Measured: 394.8993 = 0.8 ppm error.

IR (neat) ν_{max}/cm^{-1}: 3703, 3310, 2950, 2473, 1419, 1032, 777

*3-((2-Bromobenzyl)oxy)-4,5-dichloro-6-hydroxyphthalonitrile (**4aa**)*

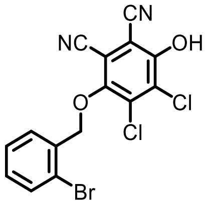

3-((2-Bromobenzyl)oxy)-4,5-dichloro-6-hydroxyphthalonitrile was prepared from 2-bromotoluene via general procedure 2 to afford a cream solid (86 mg, 54%).

^{1}H NMR (500 MHz, Acetone-d_6) δ 7.75 (dd, *J* = 7.7, 1.7 Hz, 1H), 7.68 (dd, *J* = 8.0, 1.3 Hz, 1H), 7.48 (td, *J* = 7.6, 1.3 Hz, 1H), 7.37 (td, *J* = 7.7, 1.7 Hz, 1H), 5.37 (s, 2H).

^{13}C NMR (126 MHz, Acetone-d_6) δ 154.7, 152.2, 135.7, 135.5, 133.7, 132.0, 131.6, 129.1, 128.9, 124.1, 113.3, 113.2, 110.7, 103.2, 77.3.

HRMS (Nanospray) calc: $[M-H]^-$ ($C_{15}H_6N_2O_2Cl_2Br$) 394.8990; Measured: 394.8980 = -2.5 ppm error

IR (neat) ν_{max}/cm^{-1}: 3697, 3258, 2822, 2238, 1422, 1032

*4,5-Dichloro-3-hydroxy-6-((4'-(trifluoromethyl)-[1,1'-biphenyl]-4-yl)methoxy)phthalonitrile (**4ab**)*

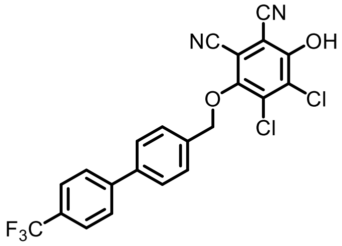

4,5-Dichloro-3-hydroxy-6-((4'-(trifluoromethyl)-[1,1'-biphenyl]-4-yl)methoxy)phthalonitrile was prepared from 4-methyl-4'-(trifluoromethyl)-1,1'-biphenyl via general procedure 2 to afford a cream solid (130 mg, 70%).

^{1}H NMR (400 MHz, Acetone-d_6) δ 7.93 (d, *J* = 8.2 Hz, 2H), 7.82 (dd, *J* = 8.3, 1.6 Hz, 4H) 7.73 (d, *J* = 8.3 Hz, 2H), 5.32 (s, 2H).

^{13}C NMR (101 MHz, Acetone-d_6) δ 154.6, 152.4, 145.1, 140.8, 136.4, 135.5, 130.6, 129.8 (d, *J* = 32.4 Hz), 129.1, 128.5, 128.3, 126.6 (q, *J* = 3.8 Hz), 125.4 (d, *J* = 271.1 Hz), 113.5, 113.2, 110.7, 103.1, 77.8.

^{19}F NMR (377 MHz, Acetone-d_6) δ -62.9 (3F, S)

HRMS (Nanospray) calc: $[M-H]^-$ ($C_{22}H_{10}N_2O_2Cl_2F_3$) 461.0071; Measured: 461.0076 = 1.1 ppm error.

IR (neat) ν_{max}/cm^{-1}: 3703, 3336, 2950, 2248, 1325, 1080, 816

*4,5-Dichloro-3-((4'-cyano-[1,1'-biphenyl]-4-yl)methoxy)-6-hydroxyphthalonitrile (**4ac**)*

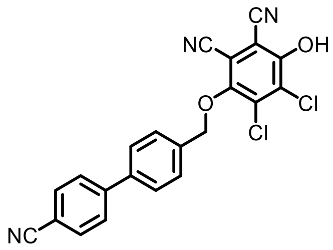

4,5-Dichloro-3-((4'-cyano-[1,1'-biphenyl]-4-yl)methoxy)-6-hydroxyphthalonitrile was prepared 4'-methyl-[1,1'-biphenyl]-4-carbonitrile via general procedure 2 to afford a cream solid (39 mg, 23%). Isolated with trace amounts of 4,5-dichloro-3,6-dihydroxyphthalonitrile hydroquinone.

^{1}H NMR (600 MHz, DMSO-*d*$_6$) δ 7.94 – 7.91 (m, 4H), 7.82 (d, *J* = 8.0 Hz, 2H), 7.65 (d, *J* = 7.8 Hz, 2H), 5.19 (s, 2H).

^{13}C NMR (151 MHz, DMSO-*d*$_6$) δ 155.1, 150.0, 144.0, 138.6, 135.7, 134.1, 132.9, 129.5, 129.2, 127.6, 127.3, 118.8, 113.6, 113.1, 110.3, 108.9, 101.8, 76.5.

HRMS (Nanospray) calc: [M-H]$^-$ ($C_{22}H_{11}Cl_2N_3O_2$) 419.0228; Measured: 419.0220 = 0.8 ppm error.

IR (neat) ν_{max}/cm^{-1}: 3403, 2255, 1654, 1023

Further Functionalisation Characterisation Data

2-Vinylanthracene-9,10-dione (**5f**)

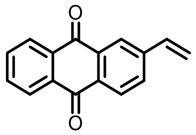

2-Vinylanthracene-9,10-dione was prepared from 4,5-dichloro-3-(1-(9,10-dioxo-9,10-dihydroanthracen-2-yl)ethoxy)-6-hydroxyphthalonitrile via general procedure 4 to afford a white solid (18 mg/ 78%). A reaction time of 18 hours was used and resulted in the formation of the vinyl product instead of the hydroxylation product.

^{1}H NMR (600 MHz, $CDCl_3$) δ 8.35-8.30 (m, 3H), 8.28 (d, *J* = 8.1 Hz, 1H), 7.84-7.77 (m, 3H), 6.87 (dd, *J* = 17.6, 10.9 Hz, 1H), 6.05 (d, *J* = 17.6 Hz, 1H) , 5.54 (d, *J* = 10.9 Hz, 1H).

^{13}C NMR (151 MHz, $CDCl_3$) δ 183.4, 182.9, 143.4, 135.5, 134.3, 134.2, 134.0, 133.8, 133.7, 132.7, 131.6, 128.0, 127.4, 127.4, 125.0, 118.5

Data are in agreement with the literature.[6]

1-(4-Nitrophenyl)ethan-1-ol (6b)

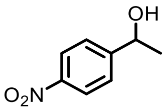

1-(4-Nitrophenyl)ethan-1-ol was prepared from 4,5-dichloro-3-hydroxy-6-(1-(4-nitrophenyl)ethoxy)phthalonitrile via general procedure 4 affording a colourless oil (5.3 mg,32%).

^{1}H NMR (500 MHz, CDCl3) δ 8.21 – 8.18 (m, 2H), 7.55 – 7.53 (m, 2H), 5.02 (q, *J* = 6.5 Hz, 1H), 1.52 (d, *J* = 6.5 Hz, 3H).

^{13}C NMR (126 MHz, CDCl3) δ 153.2, 147.3, 126.3, 123.9, 69.6, 25.6.

Data are in agreement with the literature. [7]

Ethyl 2-hydroxy-2-phenylacetate (**6p**)

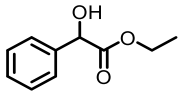

Ethyl-2-hydroxyl-2-phenylacetate was prepared from ethyl 2-(2,3-dichloro-5,6-dicyano-4-hydroxyphenoxy)-2-phenylacetate via general procedure 4 affording a colourless oil (10.8 mg, 60%).

^{1}H NMR (500 MHz, CDCl3) δ 7.44 – 7.41 (m, 2H, H4), 7.38 – 7.31 (m, 3H), 5.16 (d, *J* = 5.2 Hz, 1H), 4.22 (ddq, *J* = 47.2, 10.7, 7.1 Hz, 2H), 3.44 (d, *J* = 5.8 Hz, 1H), 1.23 (t, *J* = 7.1 Hz, 3H).

^{13}C NMR (126 MHz, CDCl3) δ 173.8, 138.6, 128.7, 128.6, 126.7, 73.0 ,62.4, 14.2.

Data are in agreement with the literature.[8]

2-(4-Fluorophenyl)-2-hydroxyacetonitrile (**6s**)

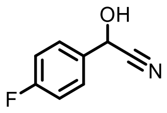

2-(4-Fluorophenyl)2-hydroxyacetonitrile was prepared from 4,5-dichloro-3-(cyano(4-fluorophenyl)methoxy)-6-hydroxyphthalonitrile via general procedure 4 affording a colourless oil (9.2 mg, 60%).

^{1}H NMR (500 MHz, Acetone-d_6) δ 7.65 – 7.60 (m, 2H), 7.26 – 7.21 (m, 2H), 6.10 (d, J = 6.4 Hz, 1H), 5.81 (dd, J = 6.3, 0.7 Hz, 1H).

^{13}C NMR (126 MHz, Acetone-d_6) δ 163.9 (d, J = 245.9 Hz), 134.5 (d, J = 3.2 Hz), 129.6 (d, J = 8.4 Hz), 120.5, 116.5 (d, J = 22.3 Hz), 62.8.

^{19}F NMR (471 MHz, Acetone-d_6) δ –114.4 (tt, J = 8.8, 5.3 Hz).

Data are in agreement with the literature.[9]

1-Bromo-4-(1-chloroethyl)benzene (**7d**)

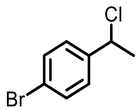

1-Bromo-4-(1-chloroethyl)benzene was prepared from 3-(1-(4-bromophenyl)ethoxy)-4,5-dichloro-6-hydroxyphthalonitrile via general procedure 5 with DCDMH to afford a colourless oil (14.2 mg ,65%).

^{1}H NMR (500 MHz, $CDCl_3$) δ 7.50 – 7.47 (m, 2H), 7.31 – 7.28 (m, 2H), 5.04 (q, *J* = 6.8 Hz, 1H), 1.82 (d, *J* = 6.8 Hz, 3H).

^{13}C NMR (126 MHz, $CDCl_3$) δ 142.0 , 131.9, 128.4, 122.3, 57.9, 26.6.

Data are in agreement with the literature.[10]

1-Bromo-2-(chloromethyl)benzene (**7aa**)

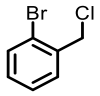

1-Bromo-2-(chloromethyl)benzene was prepared from 3-((2-bromobenzyl)oxy)-4,5-dichloro-6-hydroxyphthalonitrile via general procedure 5 with DCDMH to afford the title compound in 77% yield by GCMS analysis.

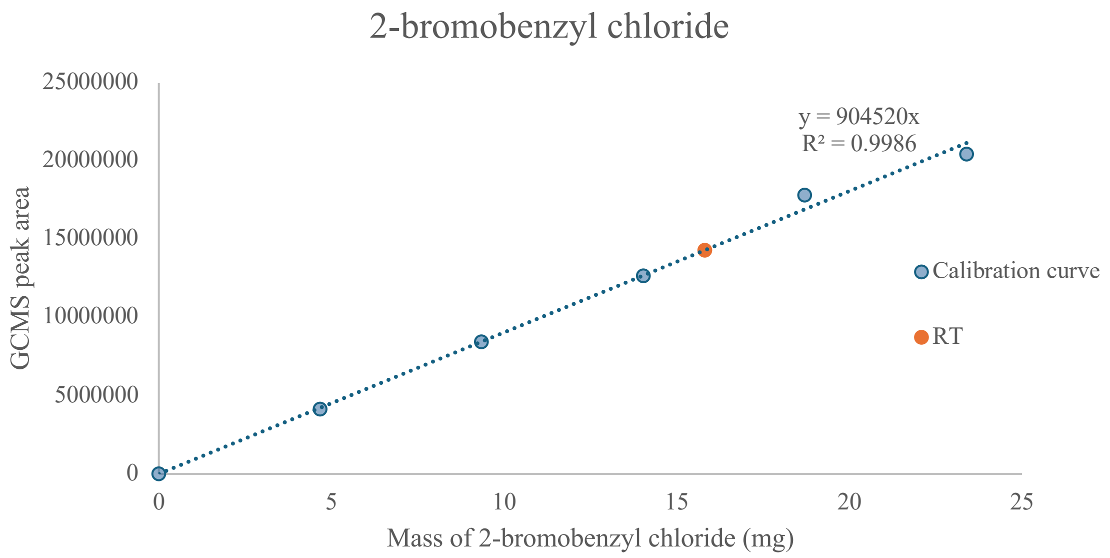

GCMS calibration curve was constructed with authentic sample purchased from a commercial supplier.

Sample: 14310127

M: 904520

Mass of product: 15.8

Percentage: 77

Ethyl 2-chloro-2-phenylacetate (**7p**)

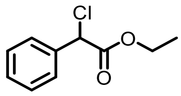

Ethyl 2-chloro-2-phenylacetate was prepared from ethyl 2-(2,3-dichloro-5,6-dicyano-4-hydroxyphenoxy)-2-phenylacetate via general procedure 5 with DCDMH affording a colourless oil (12.5 mg, 63%).

^{1}H NMR (500 MHz, $CDCl_3$) δ 7.51 – 7.48 (m, 2H), 7.40 – 7.34 (m, 3H), 5.34 (s, 1H), 4.29 – 4.16 (m, 2H), 1.26 (t, *J* = 7.1 Hz, 3H).

^{13}C NMR (126 MHz, $CDCl_3$) δ 168.5, 136.0, 129.4, 129.0, 128.1, 62.7, 59.3, 14.1.

Data are in agreement with the literature.[11]

2-Chloro-2-(4-fluorophenyl)acetonitrile (**7s**)

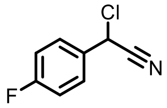

2-Chloro-2-(4-fluorophenyl)acetonitrile was prepared from 4,5-dichloro-3-(cyano(4-fluorophenyl)methoxy)-6-hydroxyphthalonitrile via general procedure 5 with DCDMH affording a colourless oil (15.6 mg, 92%).

^{1}H NMR (600 MHz, Acetone-d_6) δ 7.76 – 7.73 (m, 2H, H^4, H^6), 7.34 – 7.30 (m, 2H, H^1, H^3), 6.35 (s, 1H, H^7).

^{13}C NMR (151 MHz, Acetone-d_6) δ 164.5 (d, *J* = 248.7 Hz), 131.3 (d, *J* = 3.2 Hz), 131.2 (d, *J* = 9.2 Hz), 117.4, 117.3 (d, *J* = 22.4 Hz), 44.3.

^{19}F NMR (471 MHz, Acetone-d_6) δ –111.7 (tt, *J* = 8.8, 5.1 Hz)

HRMS (EI) calc $[M]^+$ (C_8H_5NFCl)169.0089, Measured: 169.0086 = 1.8 ppm error.

IR (neat) ν_{max}/cm^{-1}: 2982, 1744, 1214, 1141, 1024, 735

2-(1-Chloroethyl)anthracene-9,10-dione (**7f**)

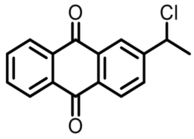

2-(1-Chloroethyl)anthracene-9,10-dione was prepared from 4,5-dichloro-3-(1-(9,10-dioxo-9,10-dihydroanthracen-2-yl)ethoxy)-6-hydroxyphthalonitrile via general procedure 5 with DCDMH affording a colourless oil (18.9 mg ,70%).

^{1}H NMR (500 MHz, $CDCl_3$) δ 8.34 – 8.30 (m, 4H), 7.90 – 7.86 (m, 1H), 7.83 – 7.79 (m, 2H), 5.21 (q, *J* = 6.8 Hz, 1H), 1.92 (d, *J* = 6.9 Hz, 3H).

^{13}C NMR (126 MHz, $CDCl_3$) δ 183.0, 182.8, 149.3, 134.4, 134.4, 133.9, 133.6, 133.6, 133.3, 132.4, 128.2, 127.5, 127.4, 125.4, 57.4, 26.4.

HRMS (EI) calc $[M]^+$($C_{16}H_{11}O_2Cl$) 270.0442, Measured: 270.0441 = 0.37 ppm error.

IR (neat) ν_{max}/cm^{-1}: 2970, 1737, 1675, 1592, 1287, 710

1-Bromo-2-(bromomethyl)benzene (**8aa**)

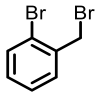

1-Bromo-2-(bromomethyl)benzene was prepared from 3-((2-bromobenzyl)oxy)-4,5-dichloro-6-hydroxyphthalonitrile via general procedure with NBS and heating to 60 °C to afford the title compound in 68% yield by GCMS analysis:

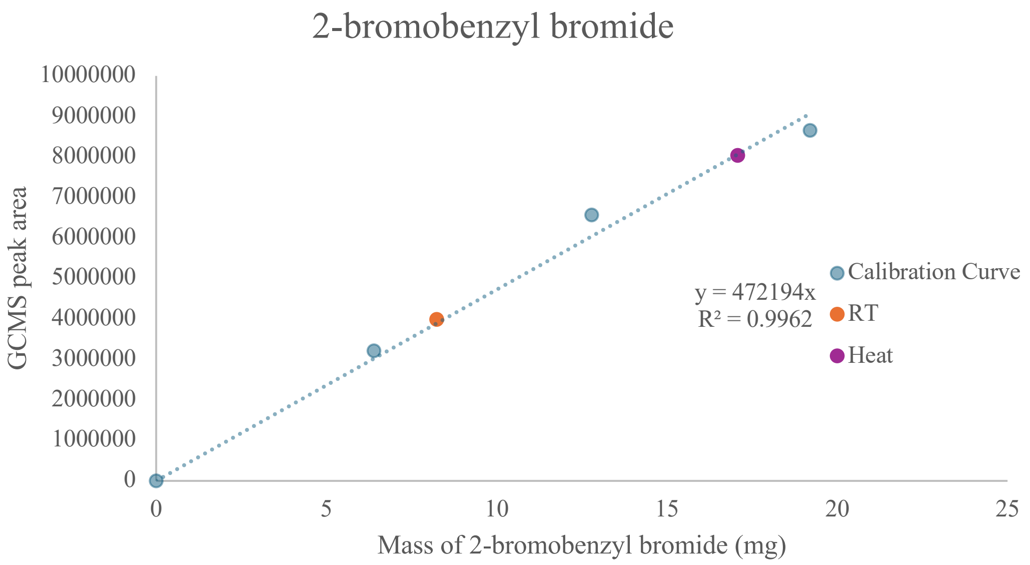

GCMS calibration curve was constructed with authentic sample purchased from a commercial supplier.

Sample: 8033057

M: 472194

Mass of product: 17.0

Percentage: 68

1-(Bromomethyl)-4-fluorobenzene (**8w**)

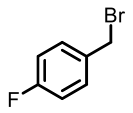

1-(Bromomethyl)-4-fluorobenzene was prepared from 4,5-dichloro-3-((4-fluorobenzyl)oxy)-6-hydroxyphthalonitrile via general procedure 5 with NBS and heating to 60 °C to afford the title compound in 33% yield by GCMS analysis.

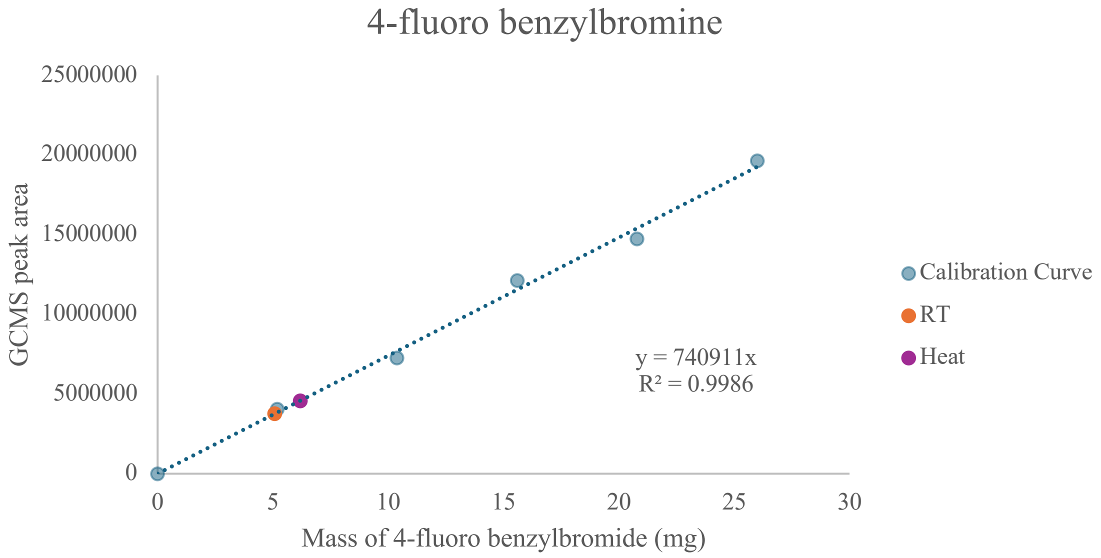

GCMS calibration curve was constructed with authentic sample purchased from a commercial supplier.

Sample: 4587084

M: 740911

Mass of product: 6.2

Percentage: 33

2-Bromo-2-(4-fluorophenyl)acetonitrile (**8s**)

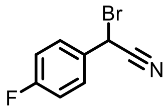

2-Bromo-2-(4-fluorophenyl)acetonitrile was prepared from 4,5-dichloro-3-(cyano(4-fluorophenyl)methoxy)-6-hydroxyphthalonitrile via general procedure 5 with NBS and 60 °C heating affording a colourless oil (15.2 mg, 71%).

^{1}H NMR (600 MHz, Acetone-d_6) δ 7.76 – 7.73 (m, 2H), 7.34 – 7.30 (m, 2H), 6.35 (s, 1H).

^{13}C NMR (151 MHz, Acetone-d_6) δ 164.5 (d, *J* = 248.7 Hz), 131.3 (d, *J* = 3.2 Hz), 131.2 (d, *J* = 9.2 Hz), 117.4, 117.3 (d, *J* = 22.4 Hz), 44.3.

^{19}F NMR (471 MHz, Acetone-d_6) δ –111.7 (tt, *J* = 8.8, 5.1 Hz)

HRMS (EI) calc: $[M\ ]^+$ (C_8H_5NFCl) 169.0089, Measured: 169.0086 = 1.8 ppm error.

IR (neat) ν_{max}/cm^{-1}: 2982, 1744, 1214, 1141, 1024, 735

NMR Spectra of Novel Compounds

Ethyl 1-(1-(4-methoxyphenyl)ethyl)-1H-pyrazole-4-carboxylate (3a)

^{1}H NMR (400 MHz, $CDCl_3$)

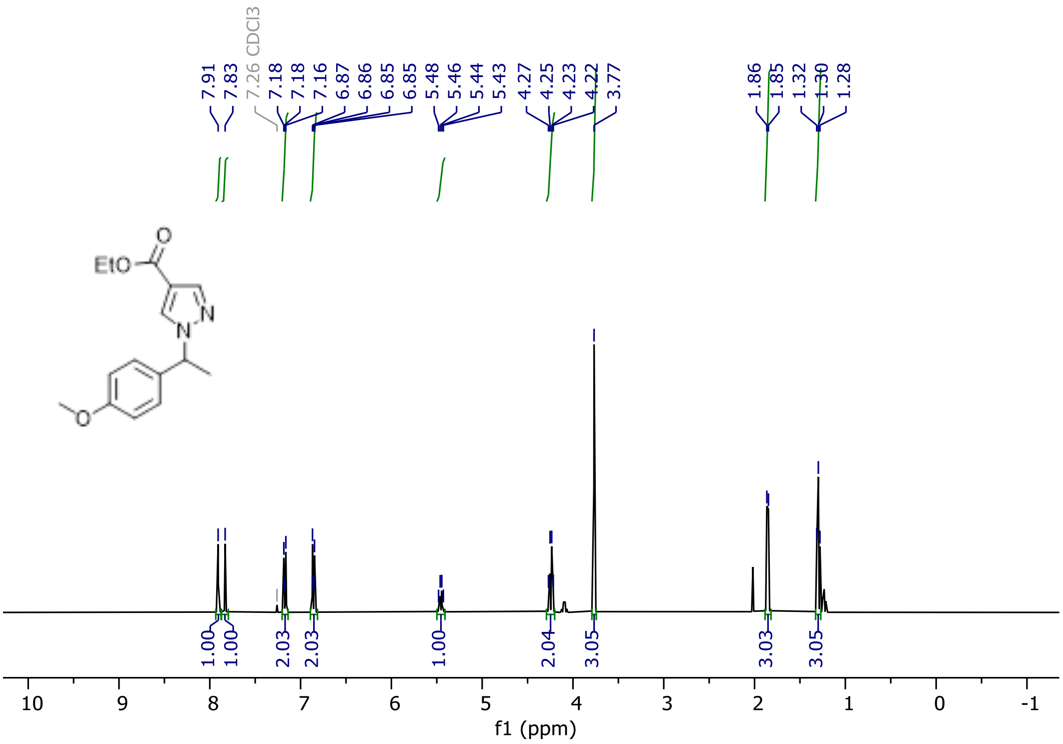

^{13}C NMR (101 MHz, $CDCl_3$)

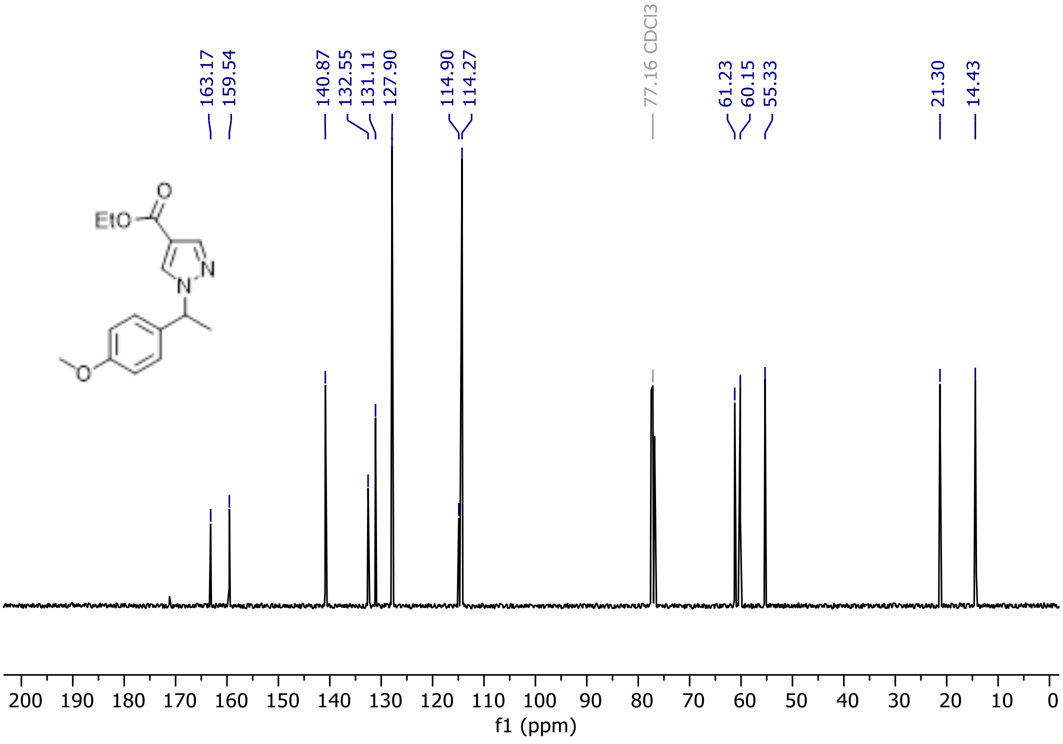

*4,5-Dichloro-3-hydroxy-6-(1-(4-nitrophenyl)ethoxy)phthalonitrile (**4b**)*

^{1}H NMR (400 MHz, MeCN-d_3)

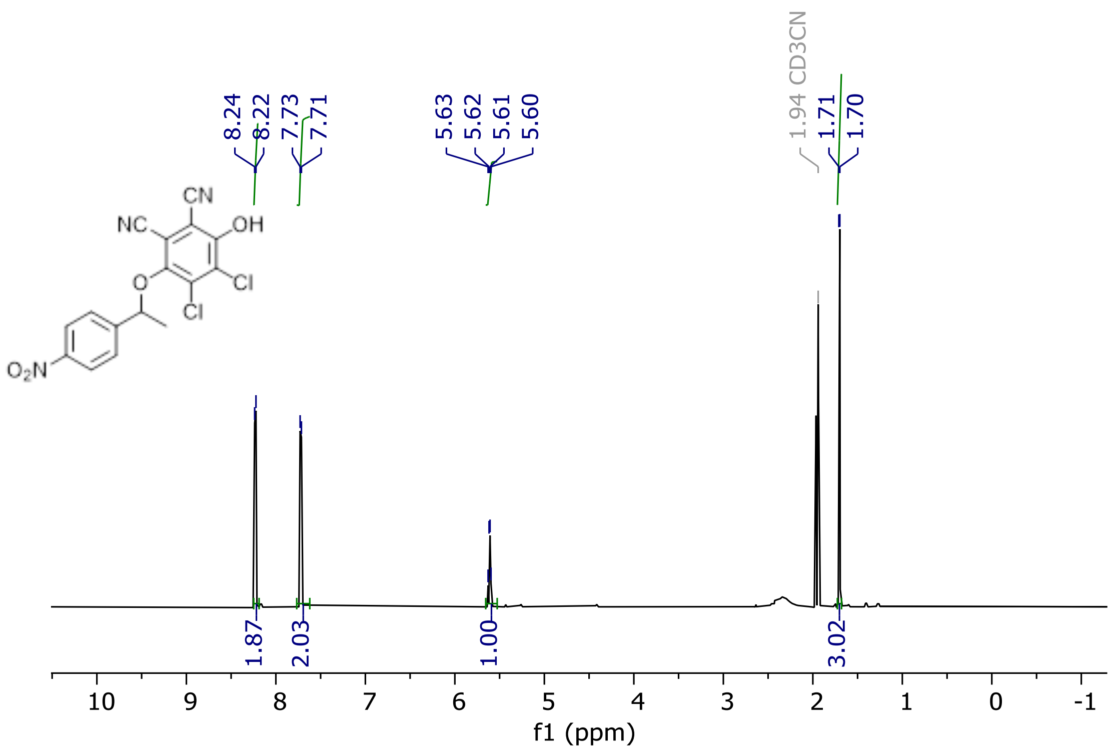

^{13}C NMR (151 MHz, MeCN-d_3)

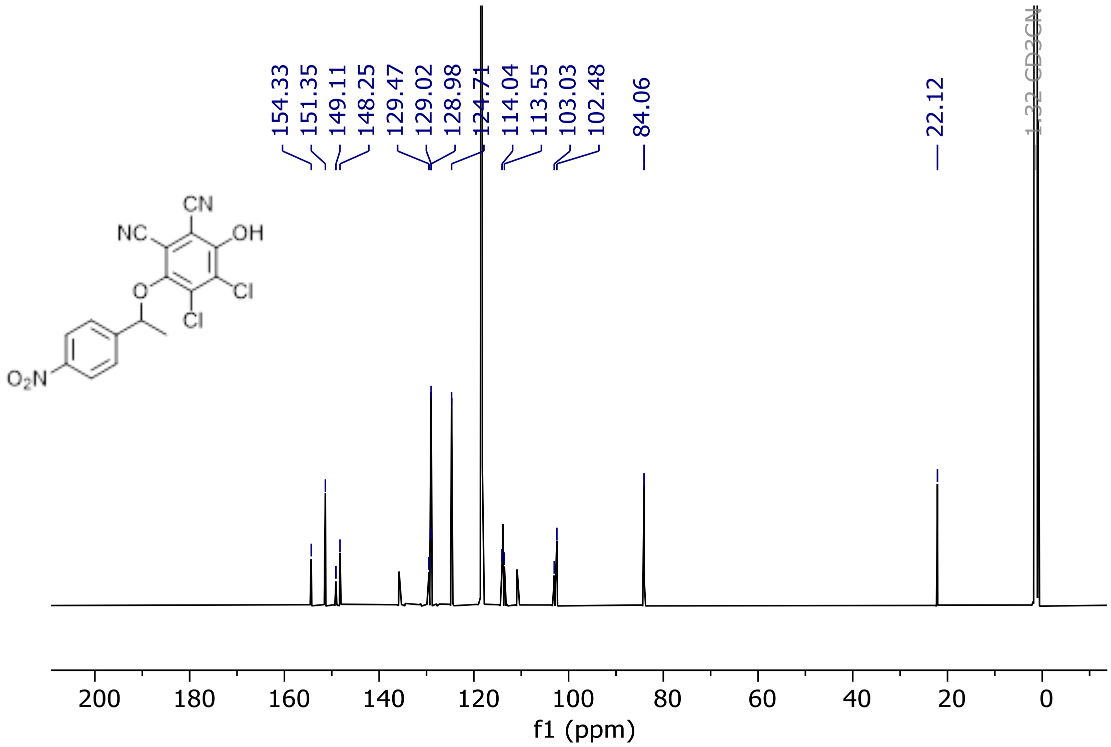

*3-(1-(4-Acetylphenyl)ethoxy)-4,5-dichloro-6-hydroxyphthalonitrile (**4c**)*

[1]**H NMR** (400 MHz, MeCN-d_3)

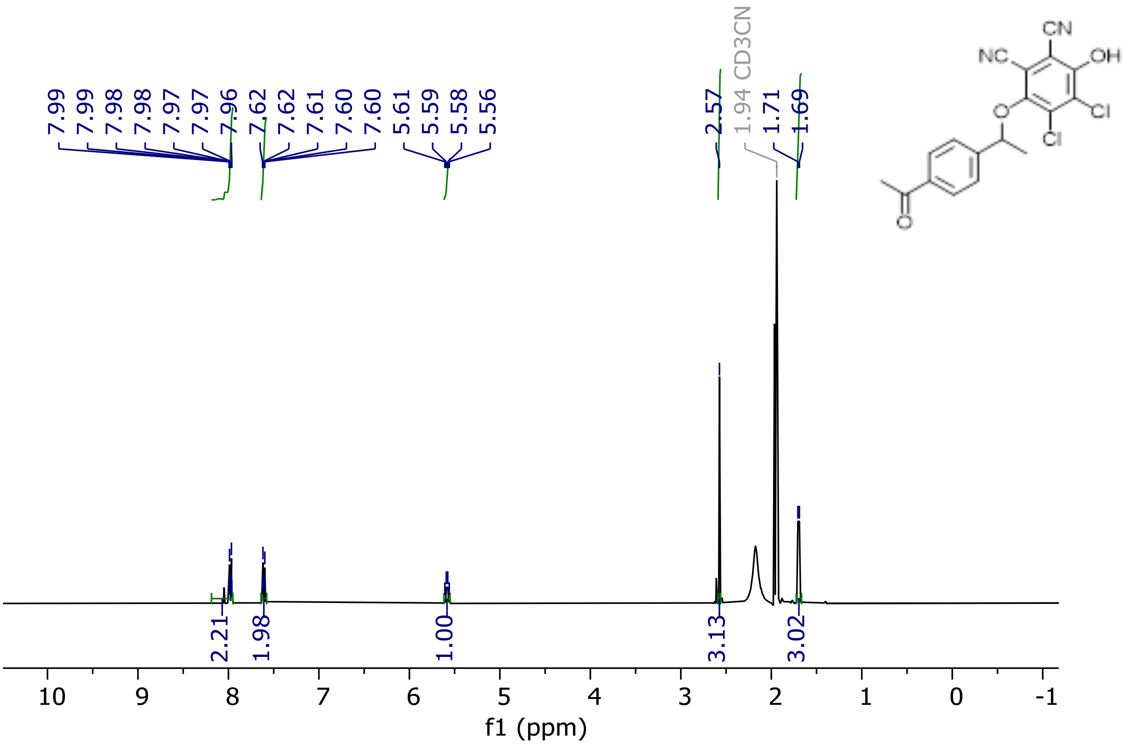

[13]**C NMR** (126 MHz, MeCN-d_3)

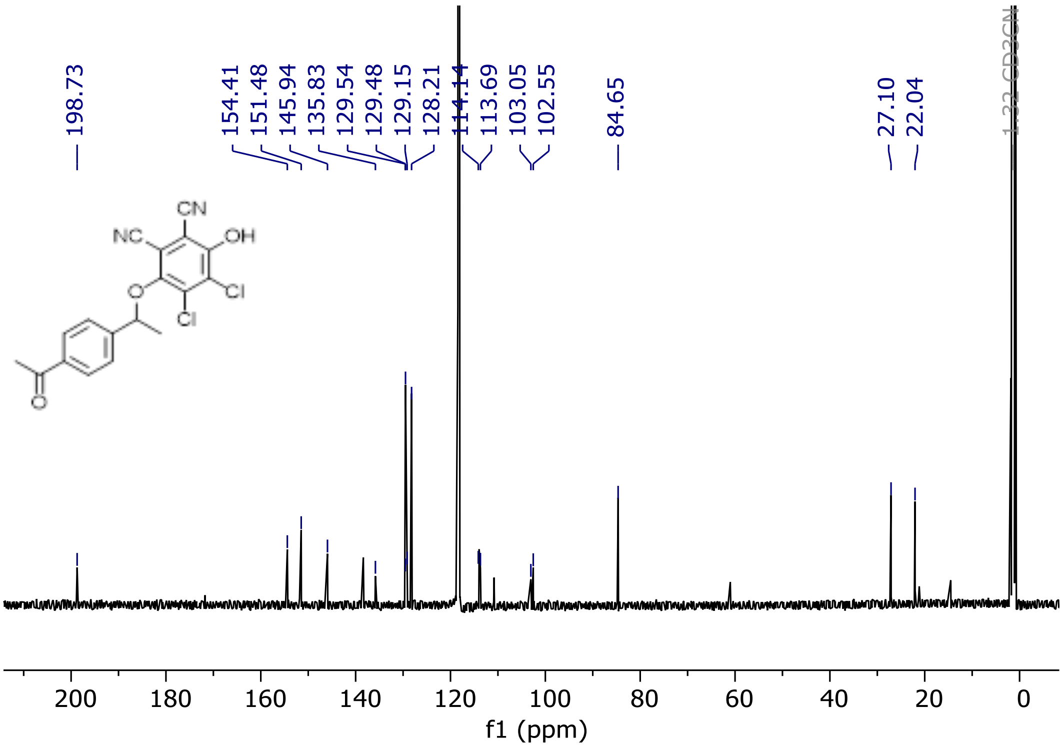

*3-(1-(4-Bromophenyl)ethoxy)-4,5-dichloro-6-hydroxyphthalonitrile (**4d**)*

^{1}H NMR (500 MHz, Acetone-d_6)

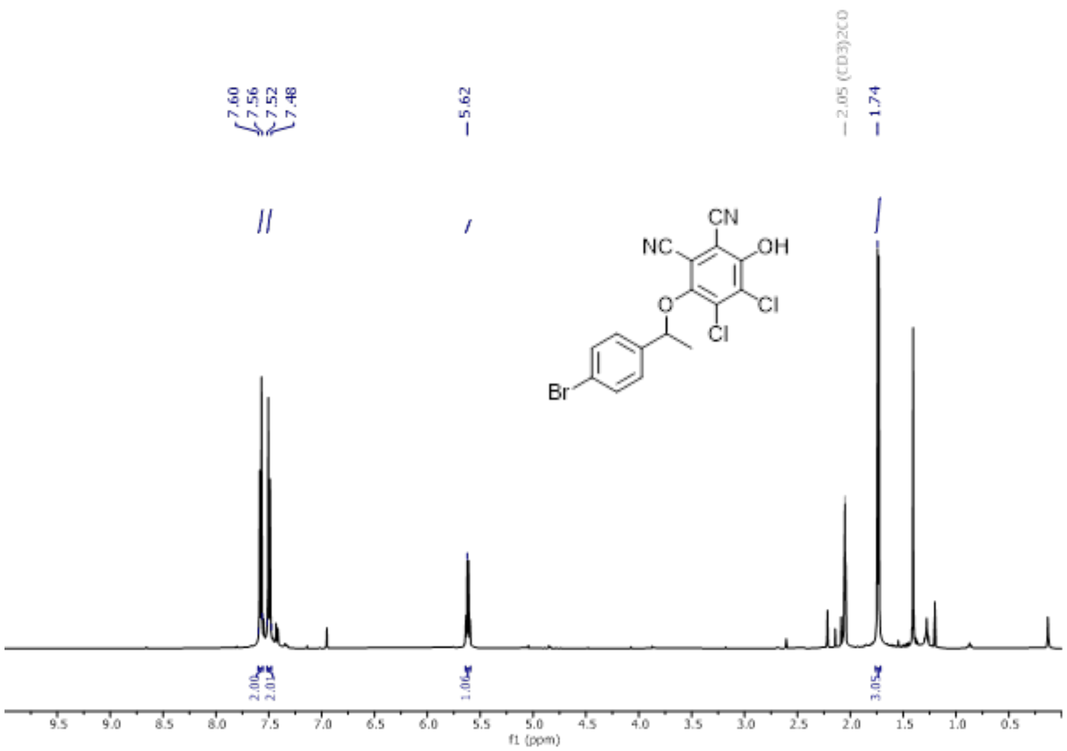

^{13}C NMR (126 MHz, Acetone-d_6)

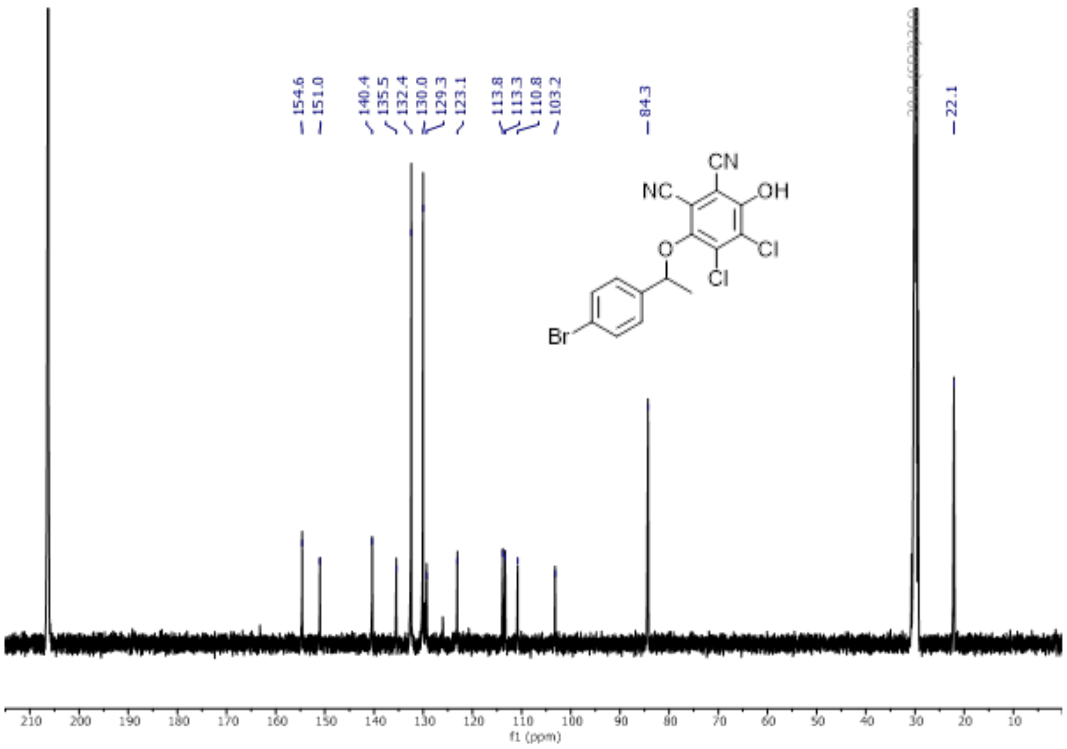

*3-(1-(4-Bromophenyl)butoxy)-4,5-dichloro-6-hydroxyphthalonitrile (**4e**)*

^{1}H NMR (500 MHz, Acetone-d_6)

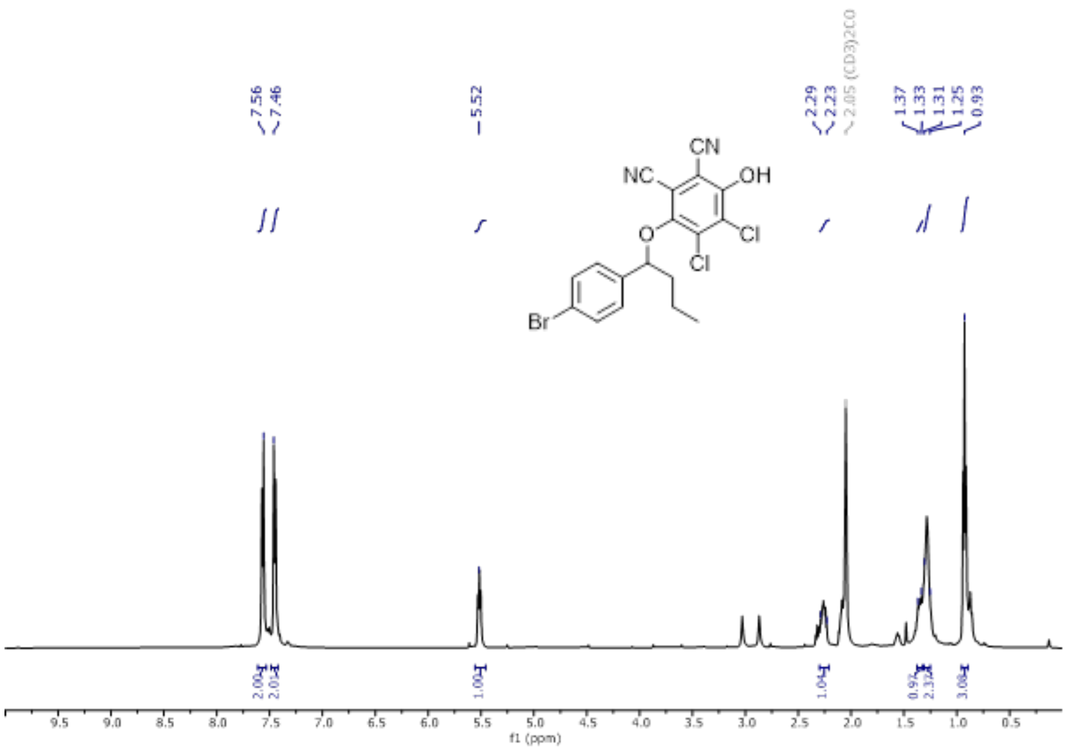

^{13}C NMR (126 MHz, Acetone-d_6)

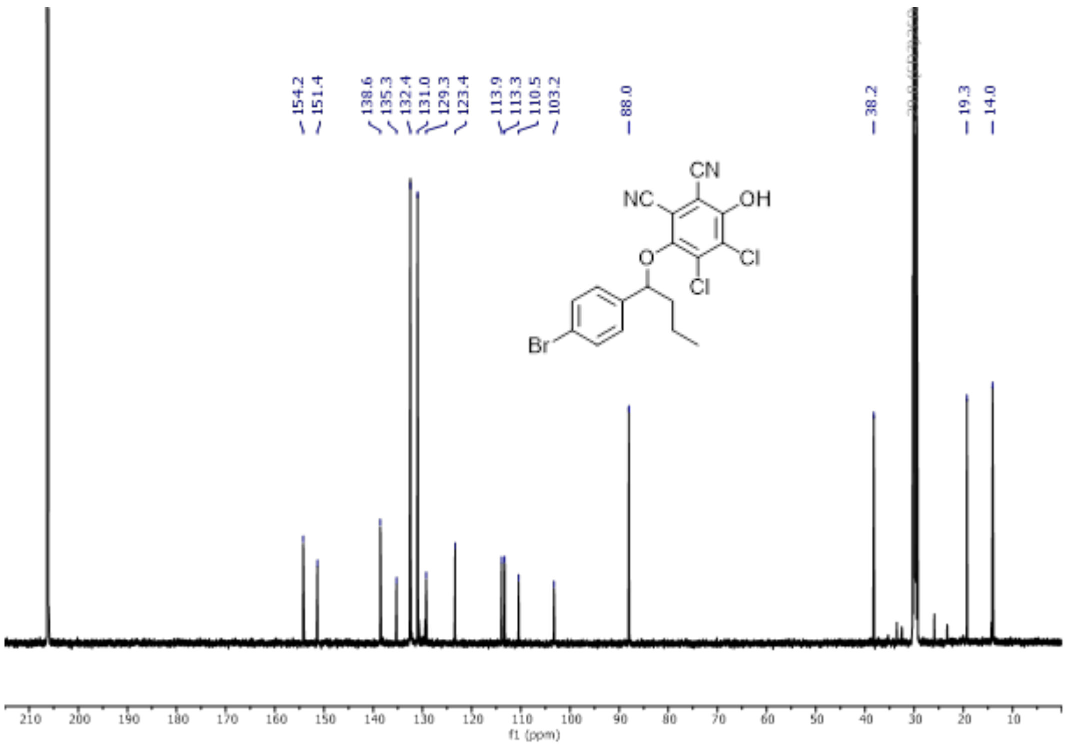

*4,5-Dichloro-3-(1-(9,10-dioxo-9,10-dihydroanthracen-2-yl)ethoxy)-6-hydroxyphthalonitrile (**4f**)*

^{1}**H NMR** (500 MHz, DMSO-d_6)

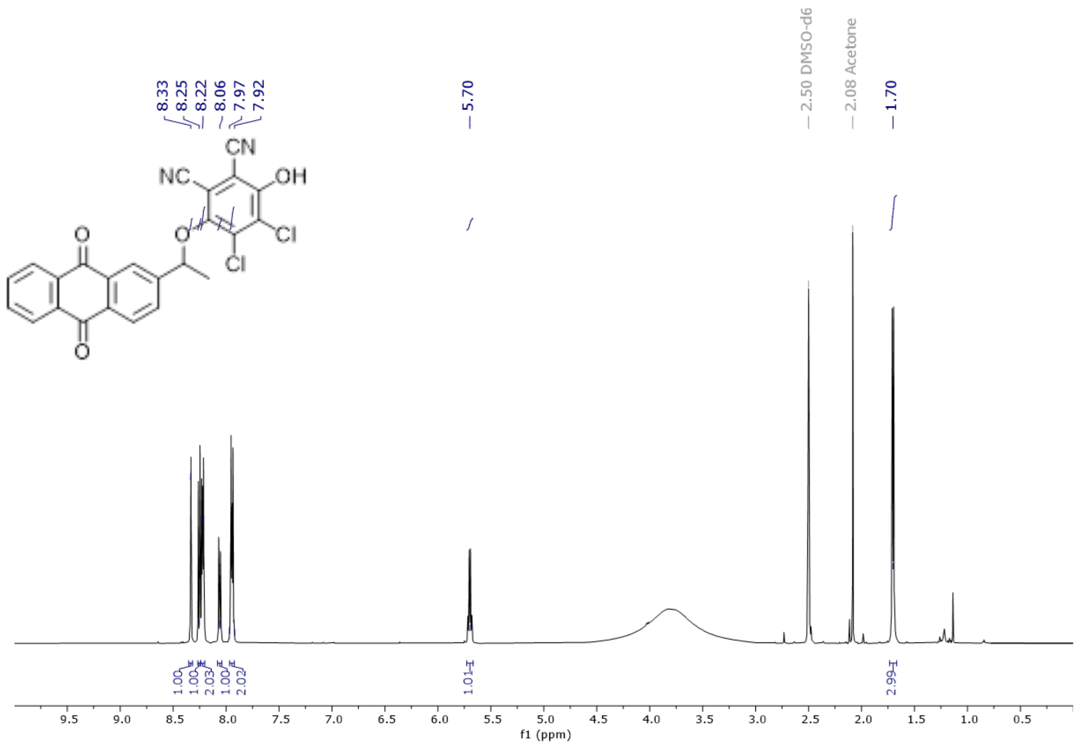

^{13}**C NMR** (126 MHz, DMSO-d_6)

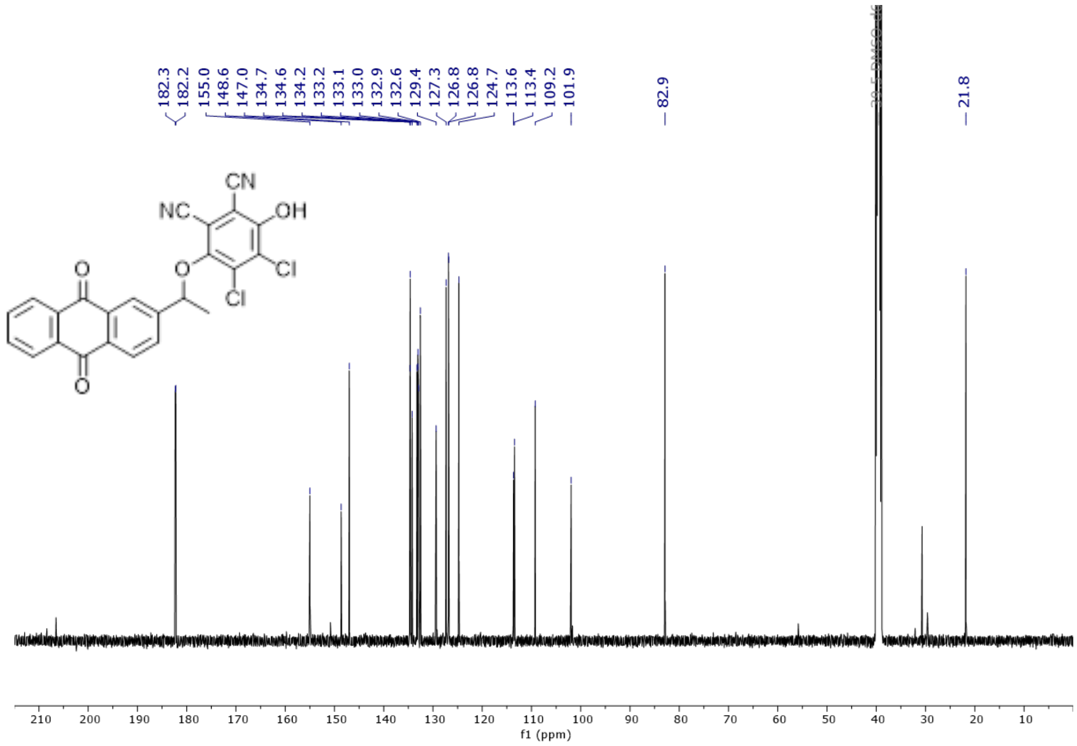

*4,5-Dichloro-3-((6-fluoro-4-oxo-1,2,3,4-tetrahydronaphthalen-1-yl)oxy)-6-hydroxyphthalonitrile (**4g**)*

^{1}H NMR (500 MHz, Acetone-d_6)

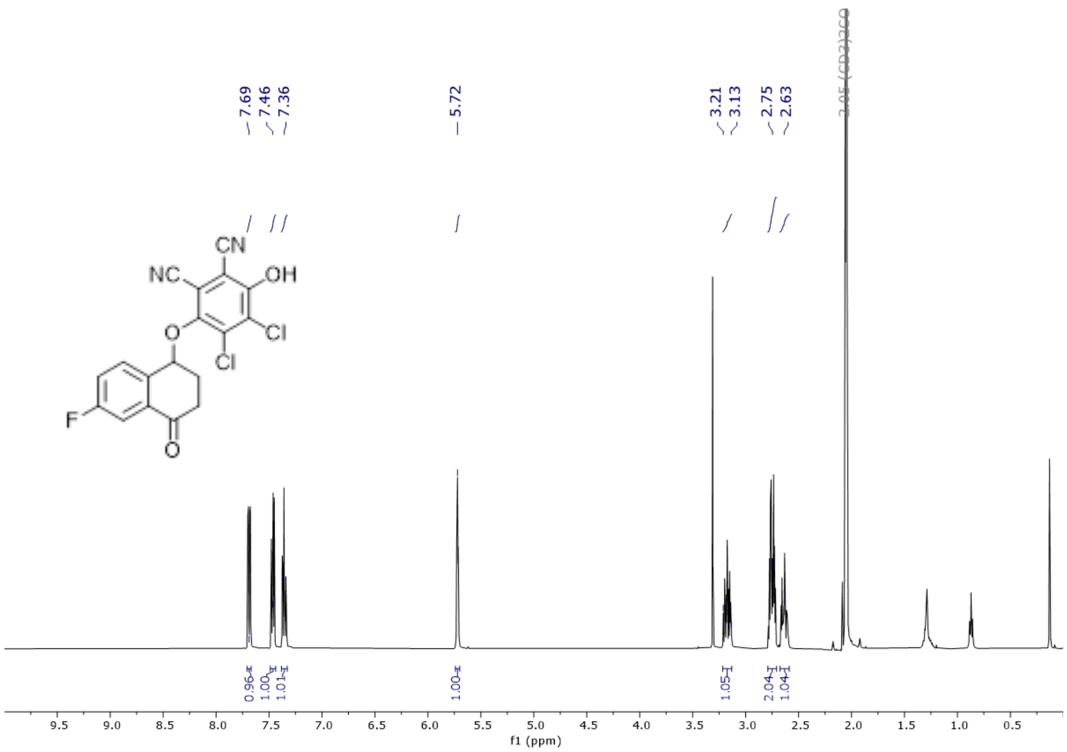

^{13}C NMR (126 MHz, Acetone-d_6)

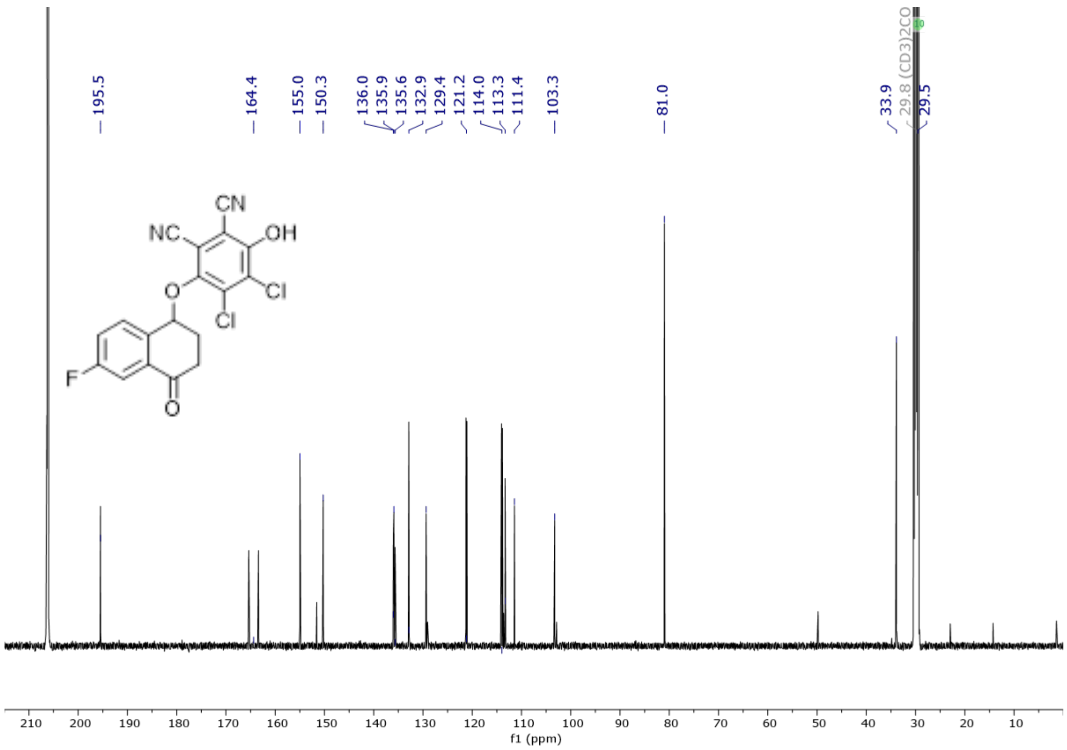

^{19}F NMR (377 MHz, Acetone-d_6)

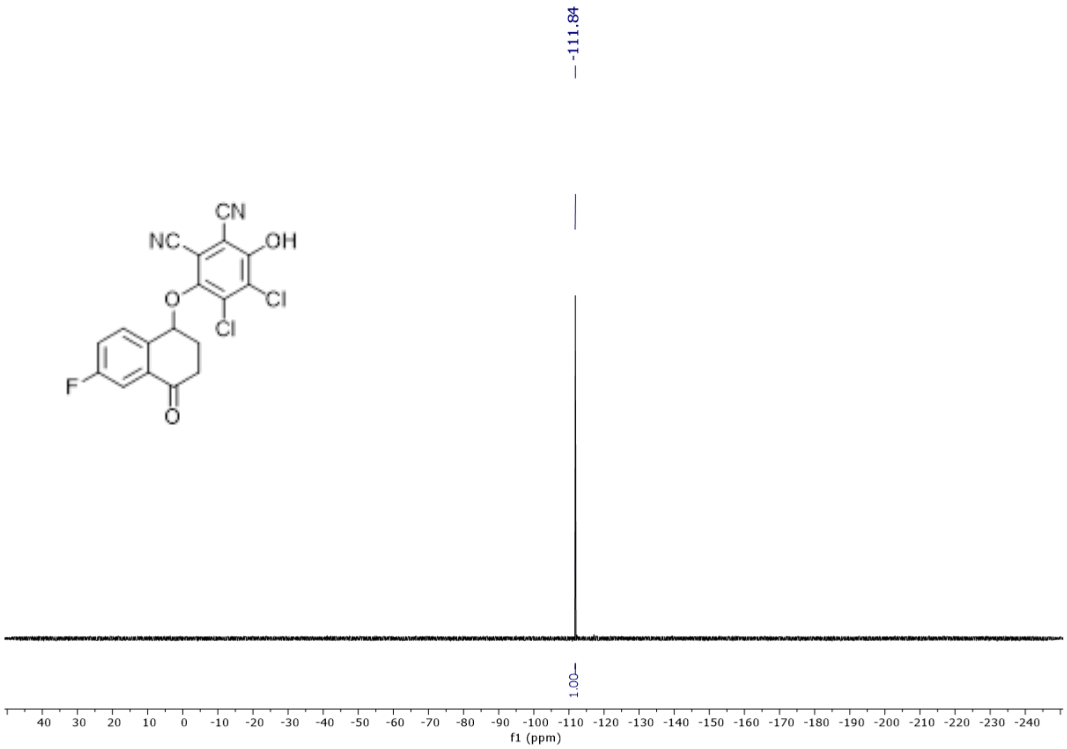

*3-(1-(3-(tert-Butyl)phenyl)ethoxy)-4,5-dichloro-6-hydroxyphthalonitrile (**4h**)*

^{1}H NMR (500 MHz, Acetone-d_6)

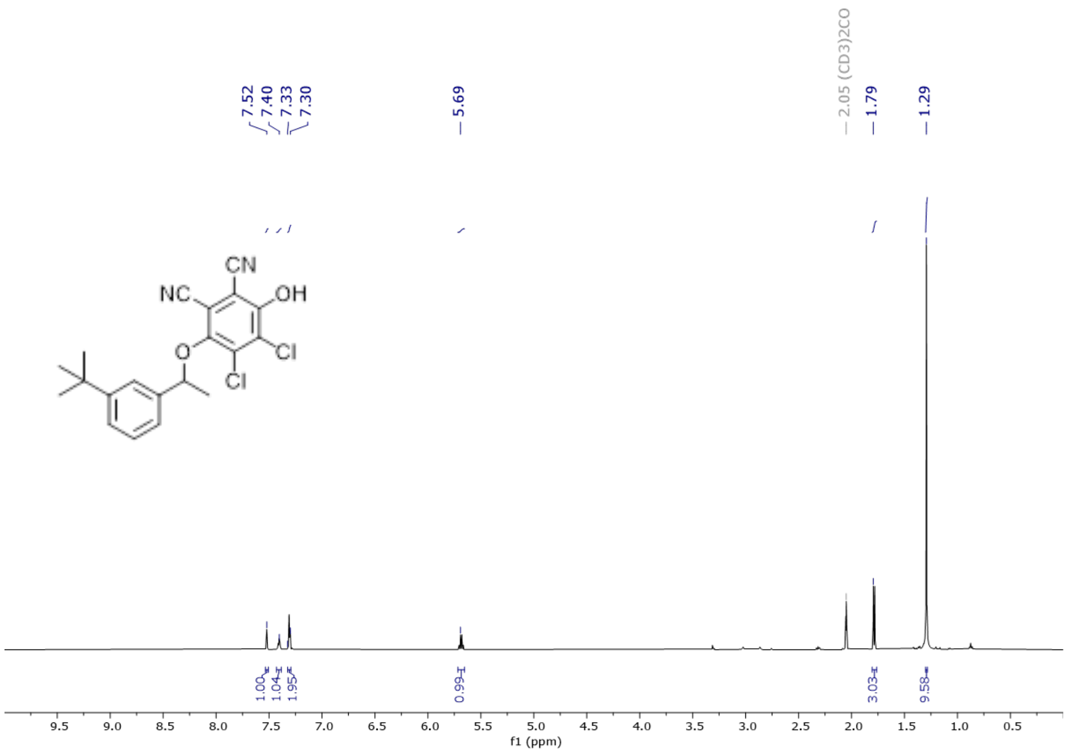

^{13}C NMR (126 MHz, Acetone-d_6)

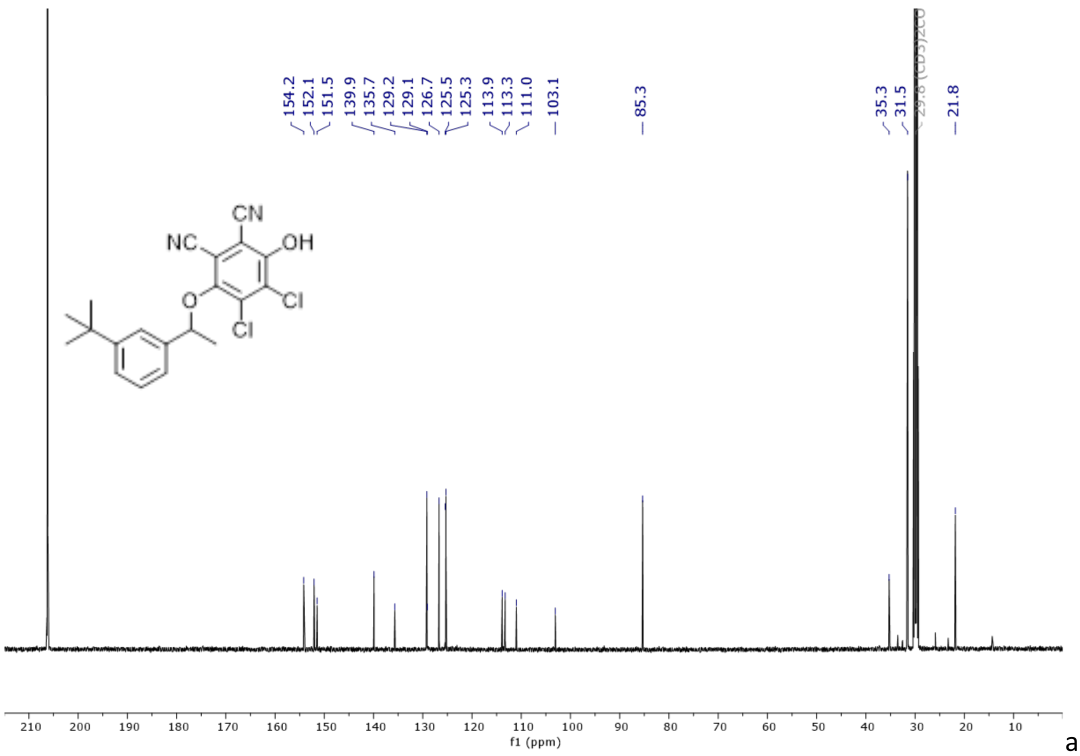

a

*4,5-Dichloro-3-hydroxy-6-((1-phenylbut-3-yn-1-yl)oxy)phthalonitrile (**4i**)*

^{1}H NMR (500 MHz, Acetone-d_6)

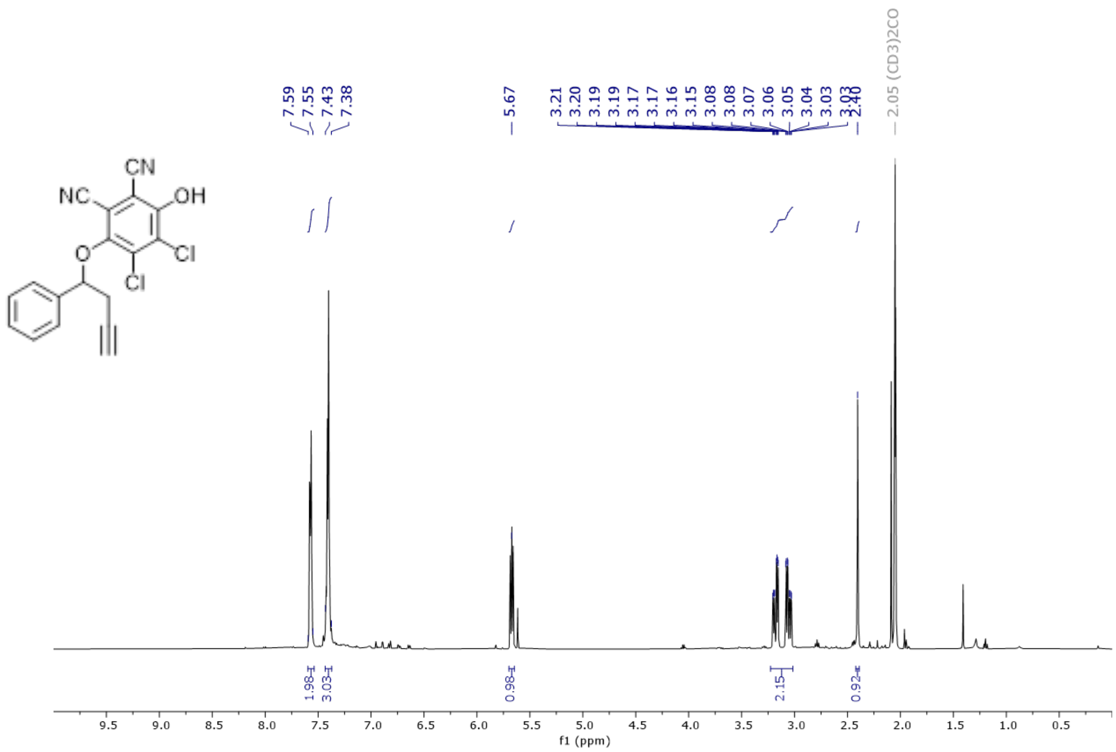

^{13}C NMR (126 MHz, Acetone-d_6)

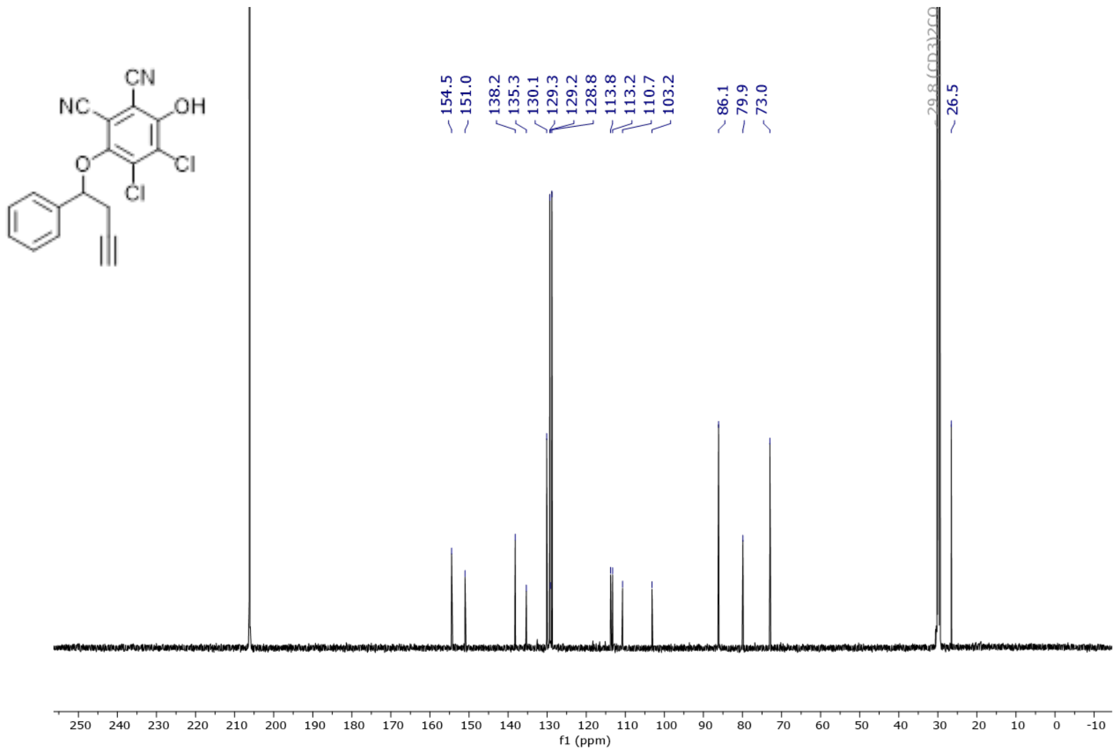

*4,5-Dichloro-3-(1-(4'-fluoro-[1,1'-biphenyl]-4-yl)ethoxy)-6-hydroxyphthalonitrile (**4j**)*

^{1}H NMR (500 MHz, Acetone-d_6)

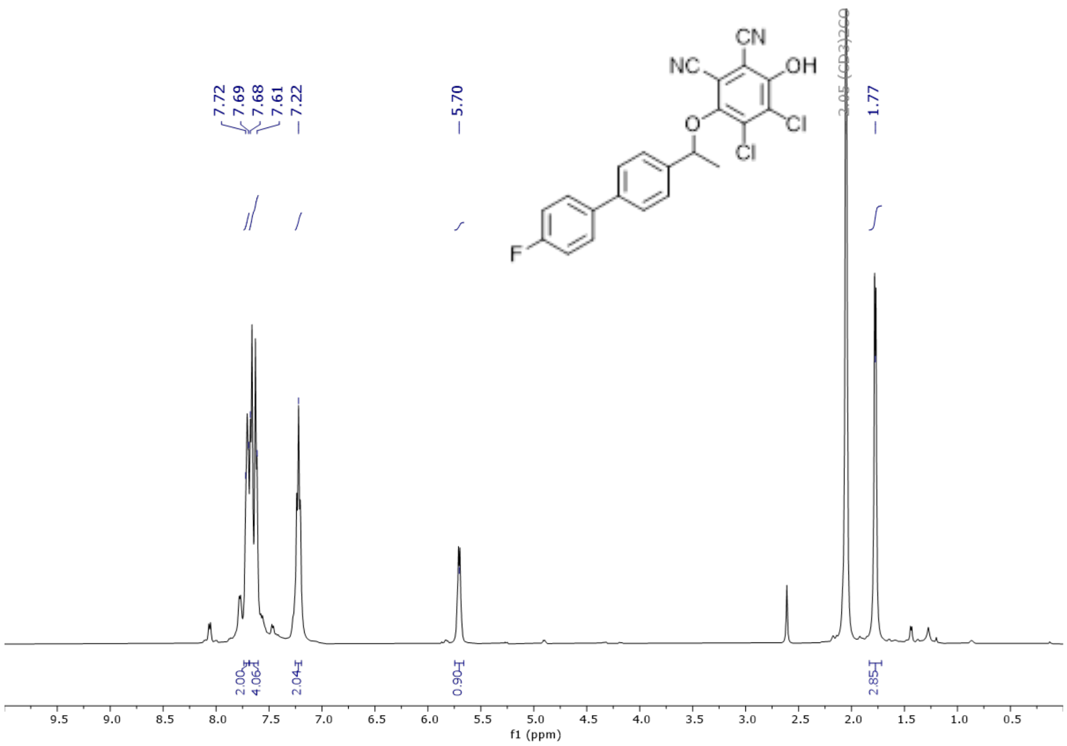

^{13}C NMR (126 MHz, Acetone-d_6)

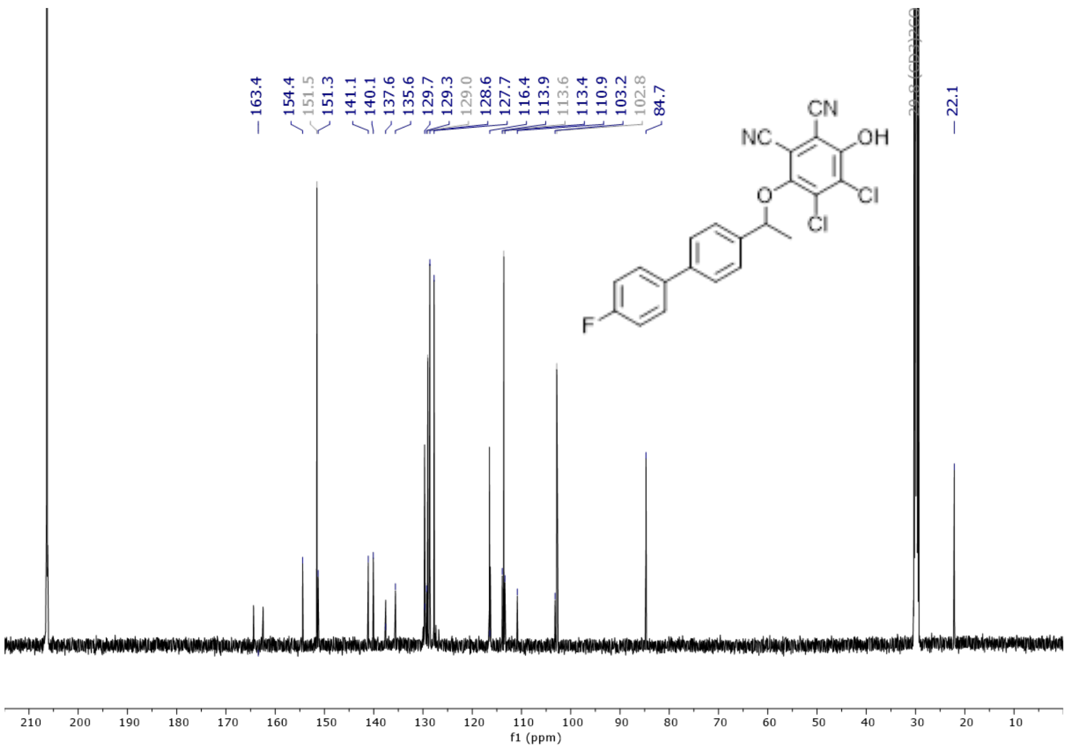

^{19}F NMR (471 MHz, Acetone-d_6)

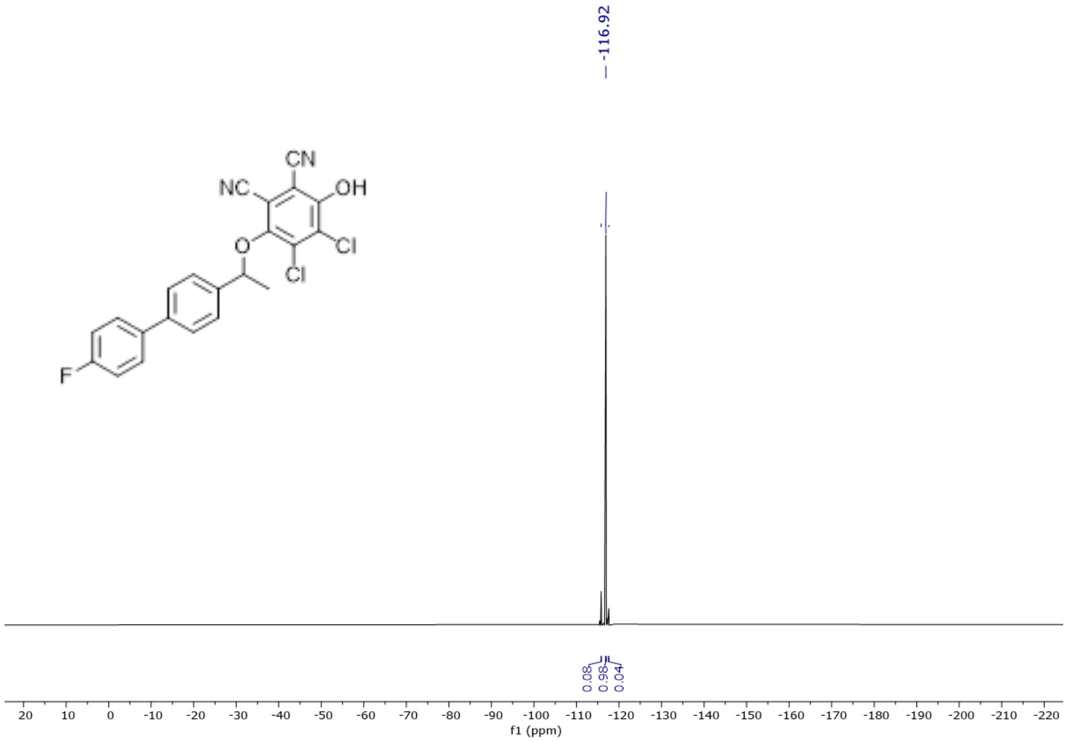

*3-(Benzhydryloxy)-4,5-dichloro-6-hydroxyphthalonitrile (**4k**)*

[1]**H NMR** (600 MHz, Acetone-d_6)

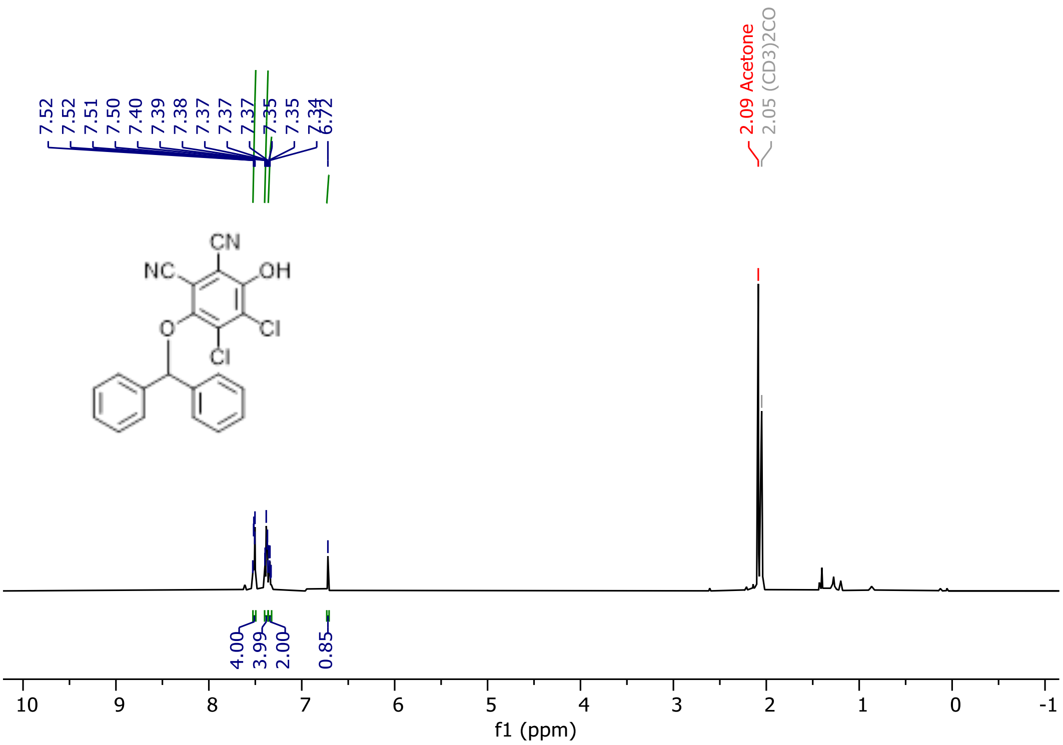

[13]**C NMR** (151 MHz, Acetone-d_6)

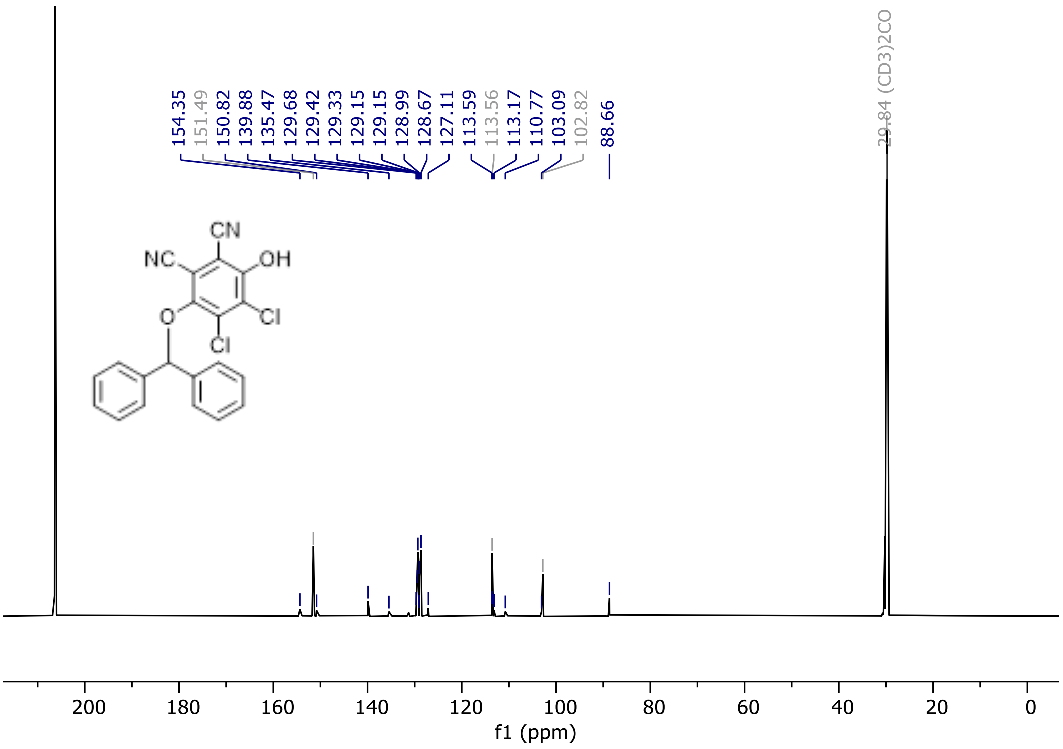

*Ethyl 2-(4-(1-(2,3-dichloro-5,6-dicyano-4-hydroxyphenoxy)-2-methylpropyl)phenyl)propanoate (**4l**)*

^{1}H NMR (400 MHz, MeCN-d_3)

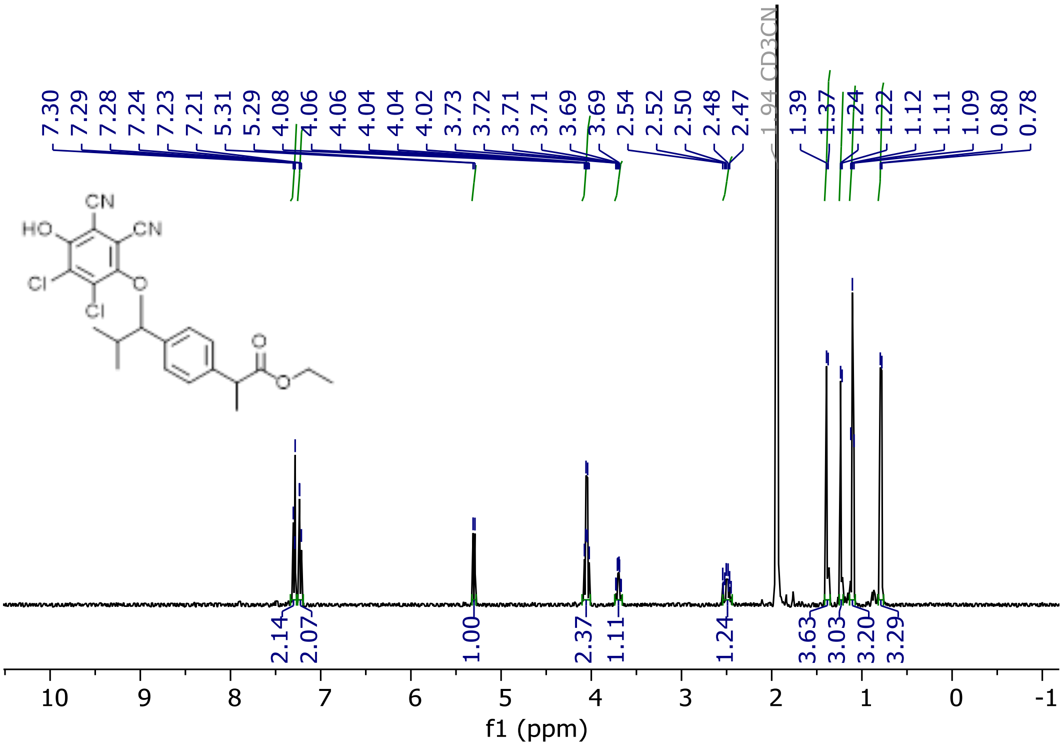

^{13}C NMR (126 MHz, MeCN-d_3)

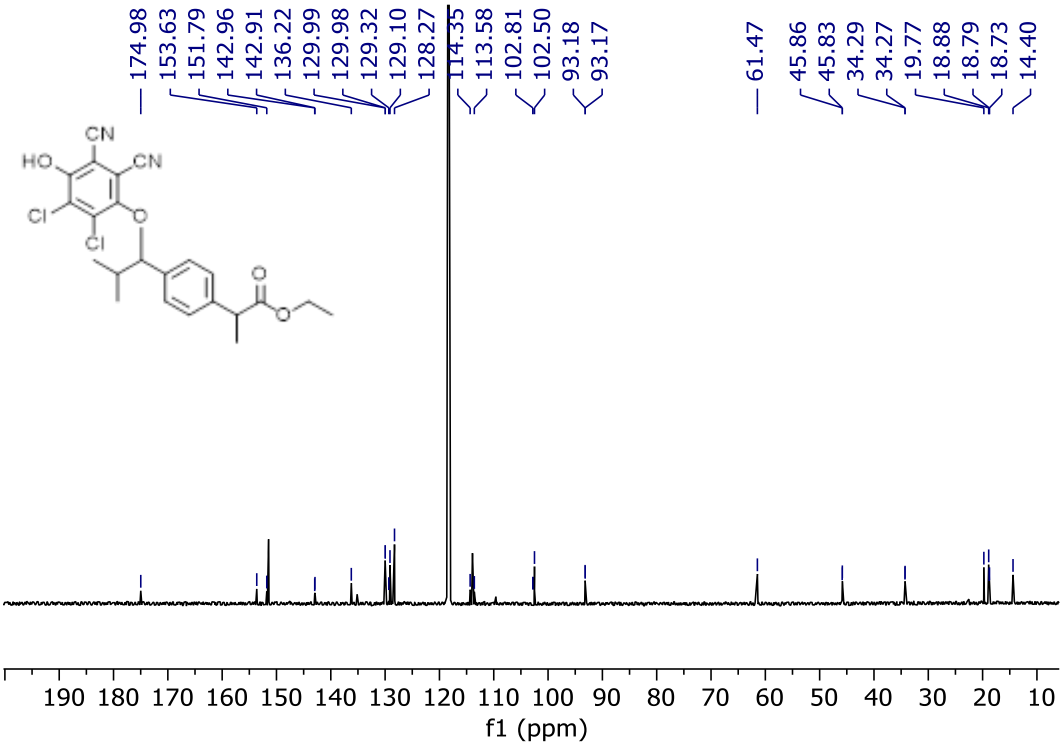

(2R)-4-(2,3-Dichloro-5,6-dicyano-4-hydroxyphenoxy)-4-phenylbutan-2-yl 2,4,6-triisopropylbenzoate ***(4m)***

^{1}H NMR (600 MHz, Acetone-d_6)

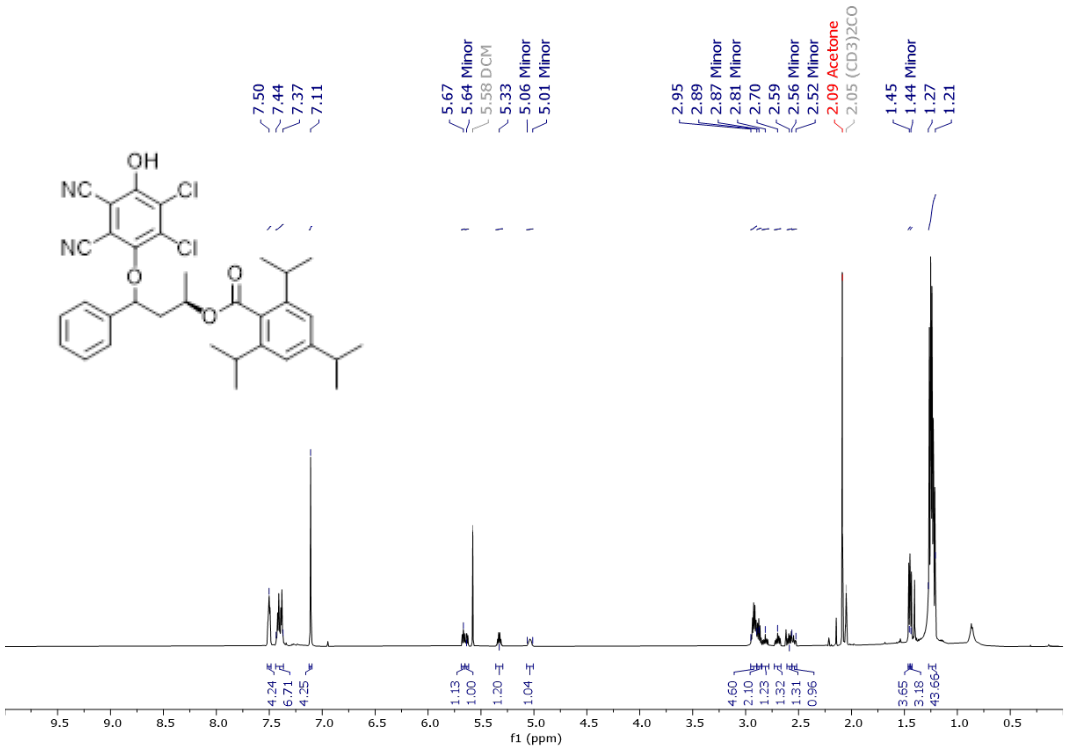

^{13}C NMR (151 MHz, Acetone-d_6)

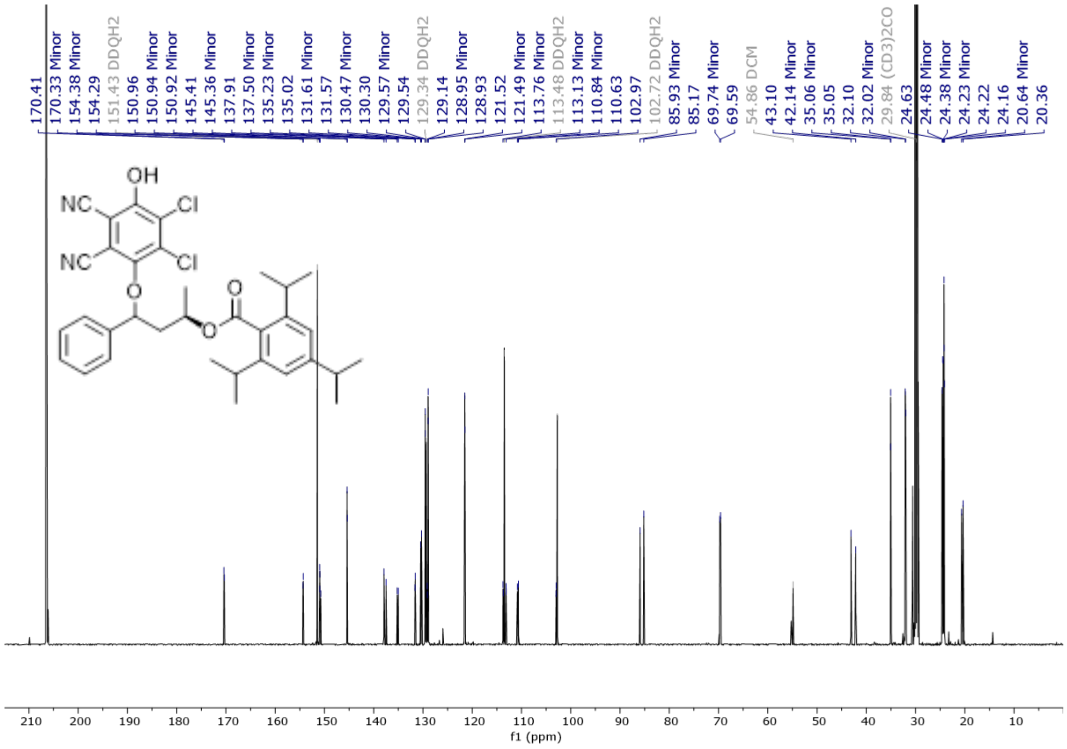

*4,5-Dichloro-3-((5-cyano-1-phenyl-3-(4,4,5,5-tetramethyl-1,3,2-dioxaborolan-2-yl)pentyl)oxy)-6-hydroxyphthalonitrile (**4n**)*

^{1}H NMR (500 MHz, Acetone-d_6)

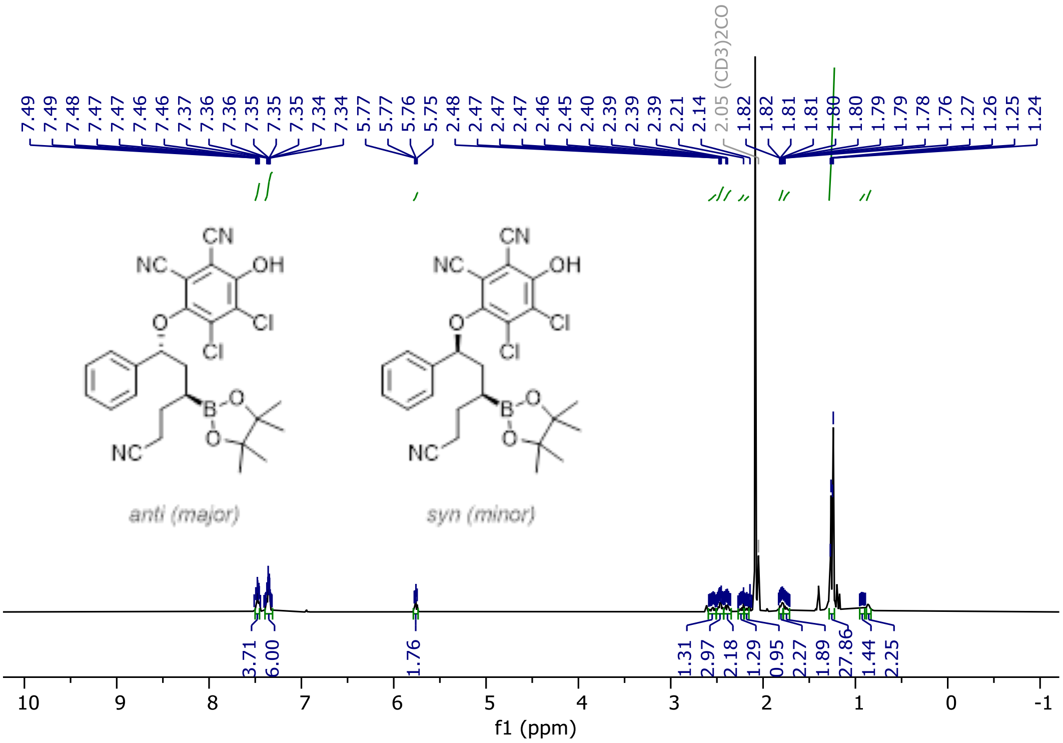

^{13}C NMR (126 MHz, Acetone-d_6)

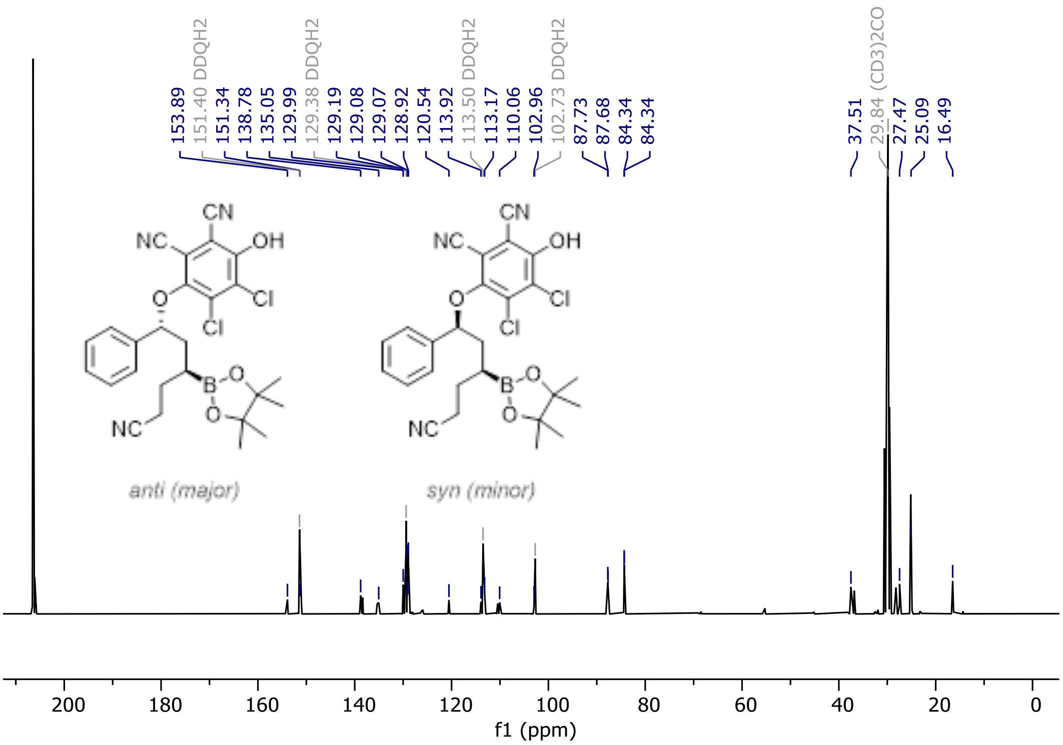

*Ethyl 2-(4-bromophenyl)-2-(2,3-dichloro-5,6-dicyano-4-hydroxyphenoxy)acetate (**4o**)*

^{1}H NMR (500 MHz, Acetone-d_6)

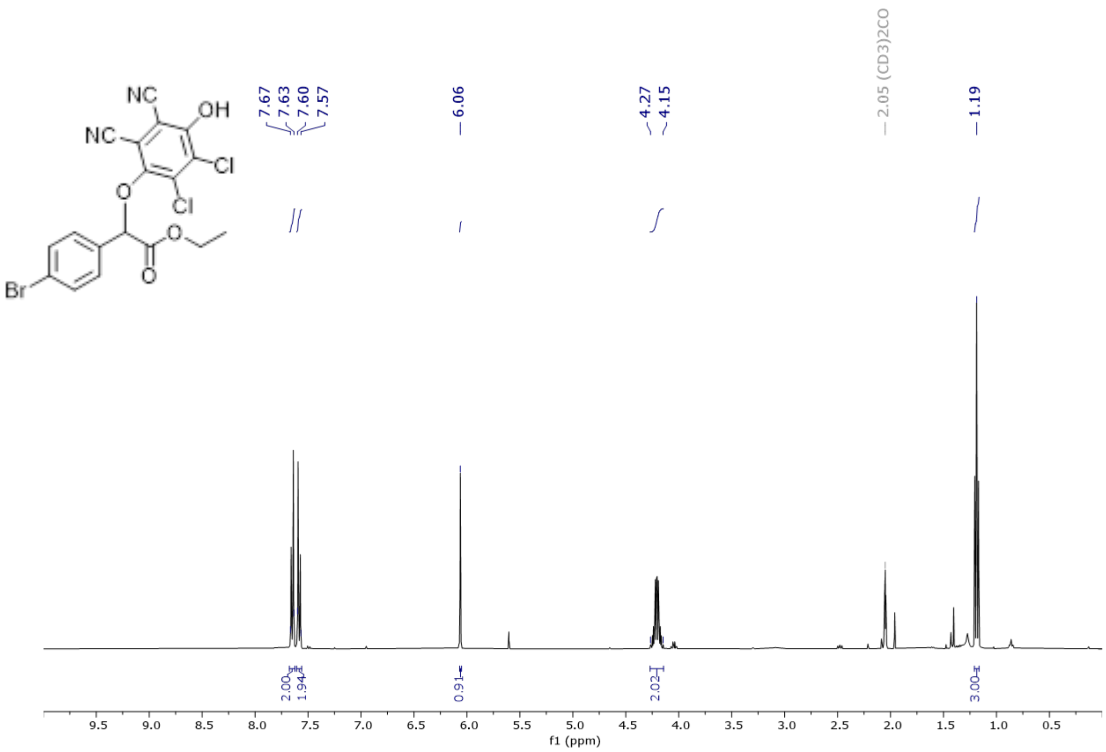

^{13}C NMR (101 MHz, Acetone-d_6)

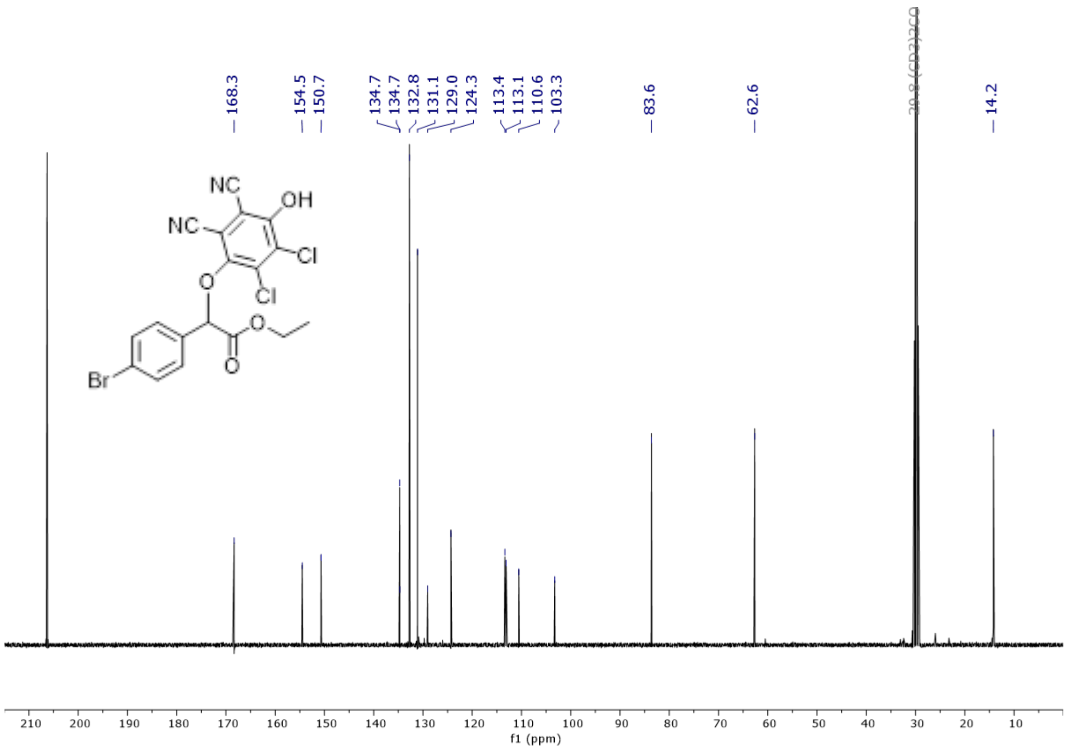

*Ethyl 2-(2,3-dichloro-5,6-dicyano-4-hydroxyphenoxy)-2-phenylacetate (**4p**)*

^{1}H NMR (400 MHz, Acetone-d_6)

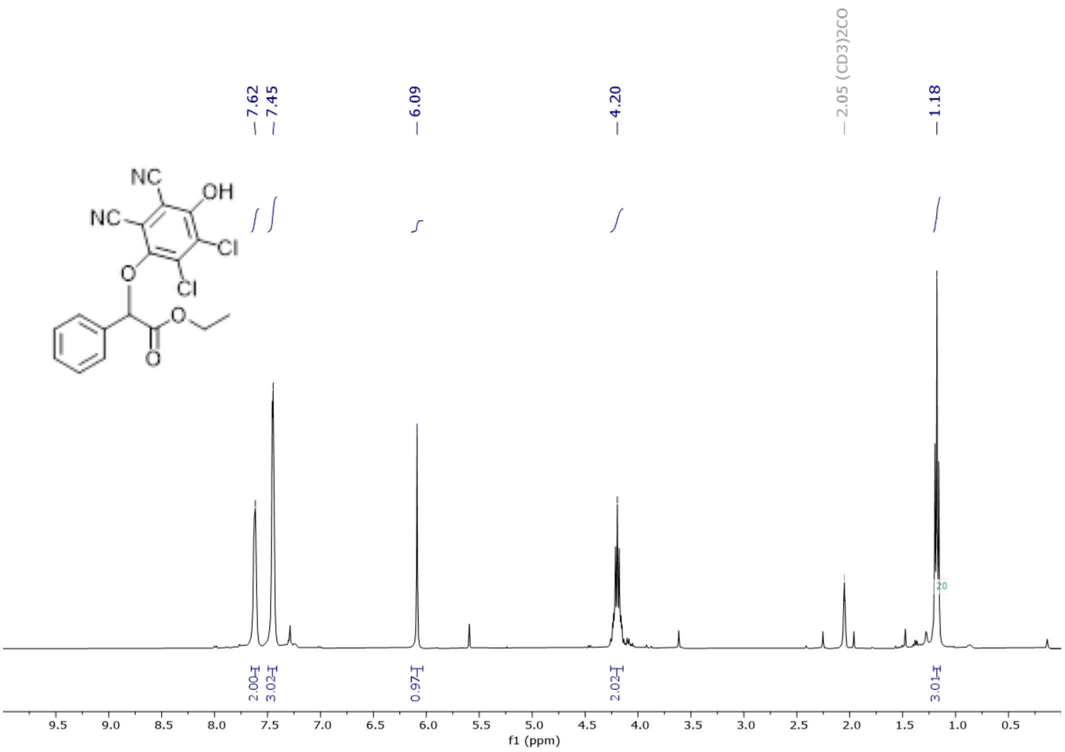

^{13}C NMR (101 MHz, Acetone-d_6)

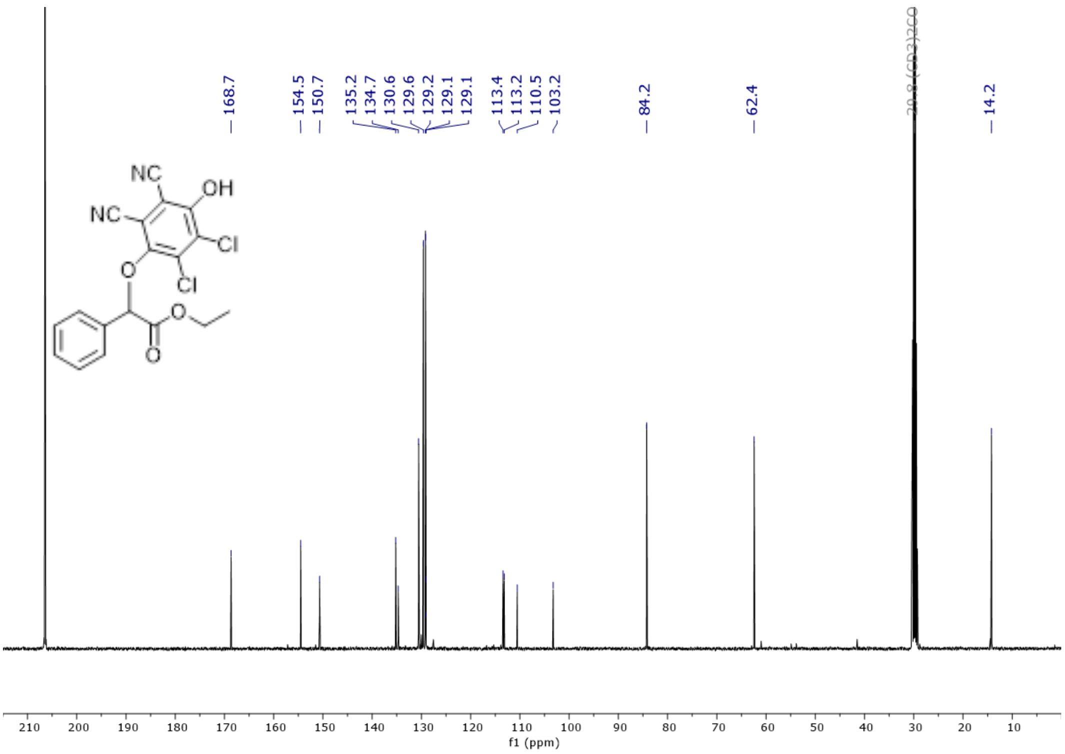

*Ethyl 2-(2,3-dichloro-5,6-dicyano-4-hydroxyphenoxy)-2-(4-fluorophenyl)acetate (**4q**)*

^{1}H NMR (400 MHz, Acetone-d_6)

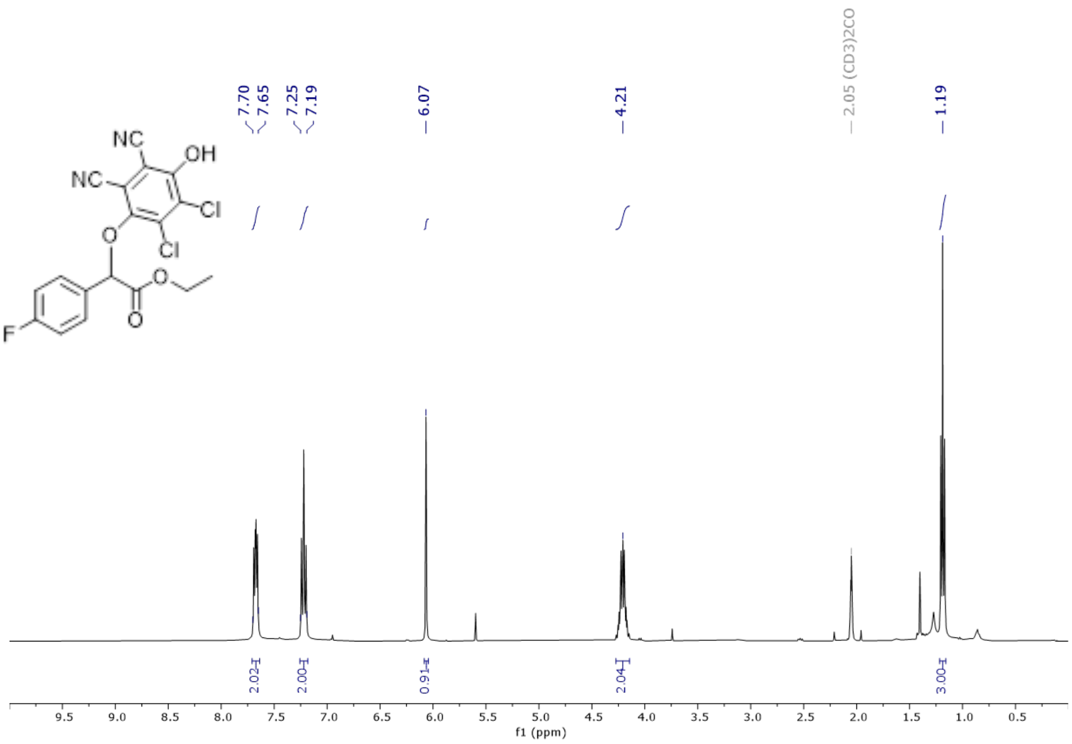

^{13}C NMR (101 MHz, Acetone-d_6)

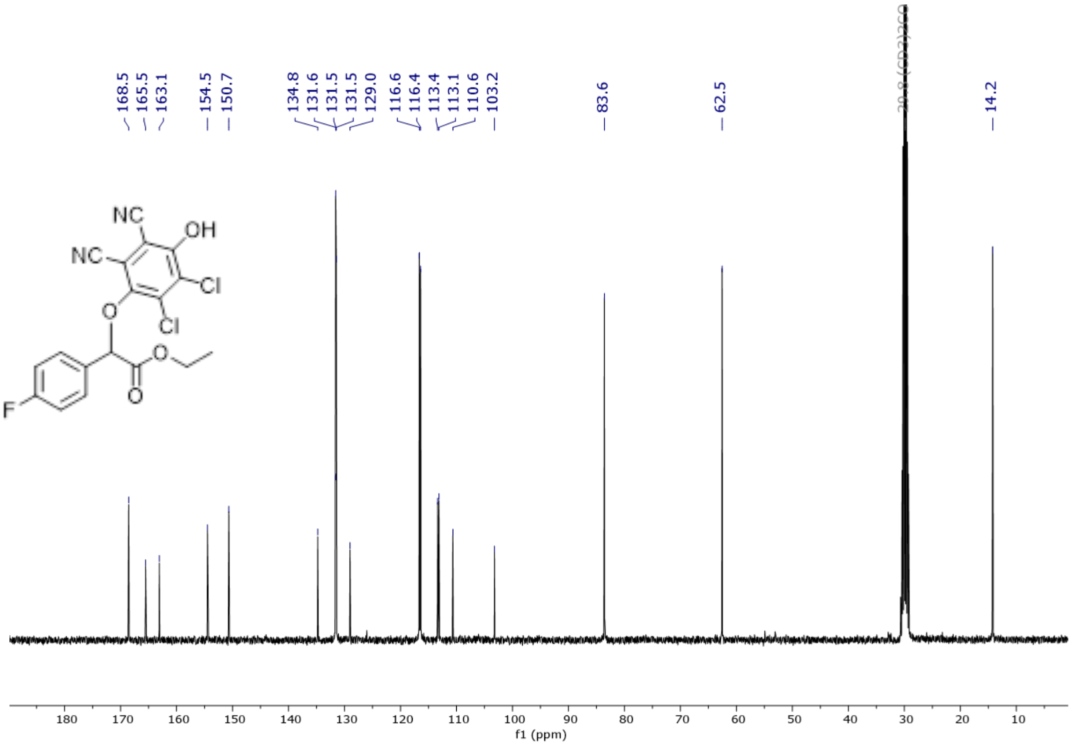

^{19}F NMR (377 MHz, Acetone-d_6)

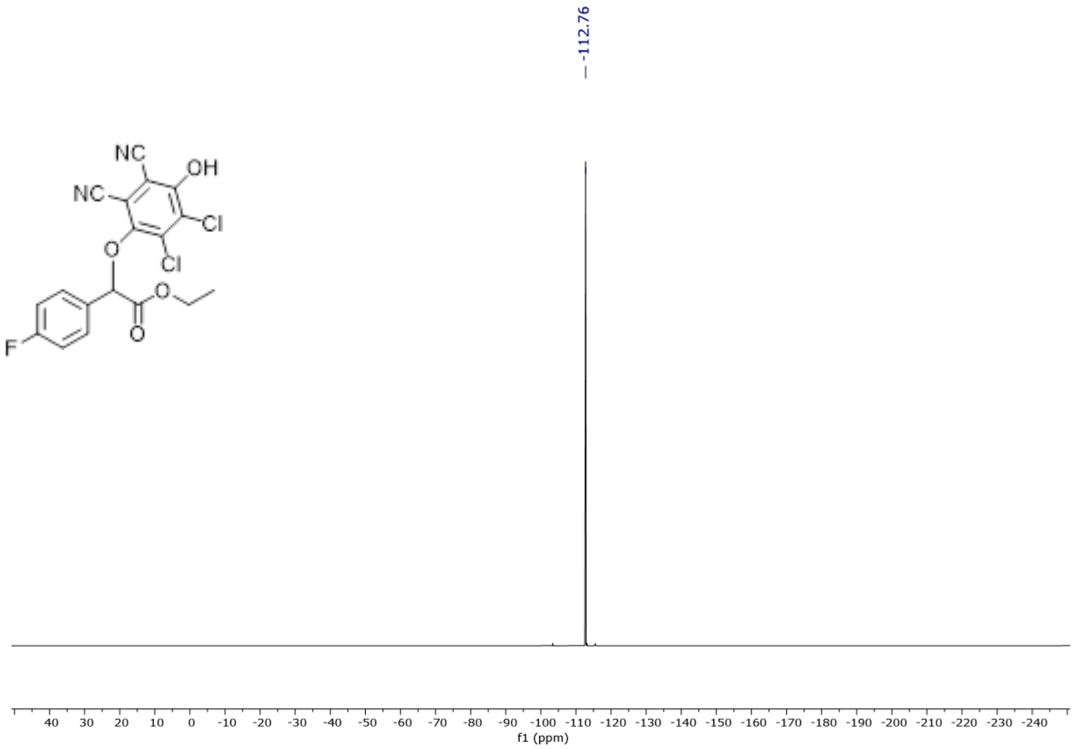

*Butyl 2-(2,3-dichloro-5,6-dicyano-4-hydroxyphenoxy)-2-(4-fluorophenyl)acetate (**4r**)*

^{1}H NMR (400 MHz, Acetone-d_6)

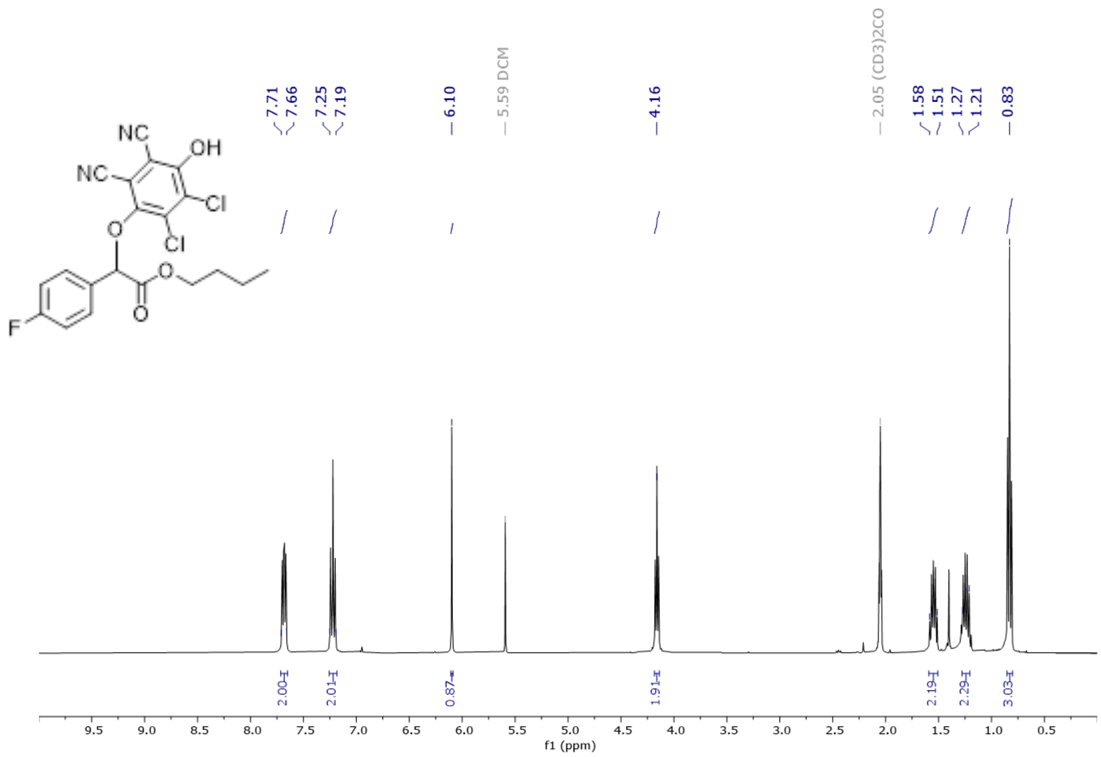

^{13}C NMR (101 MHz, Acetone-d_6)

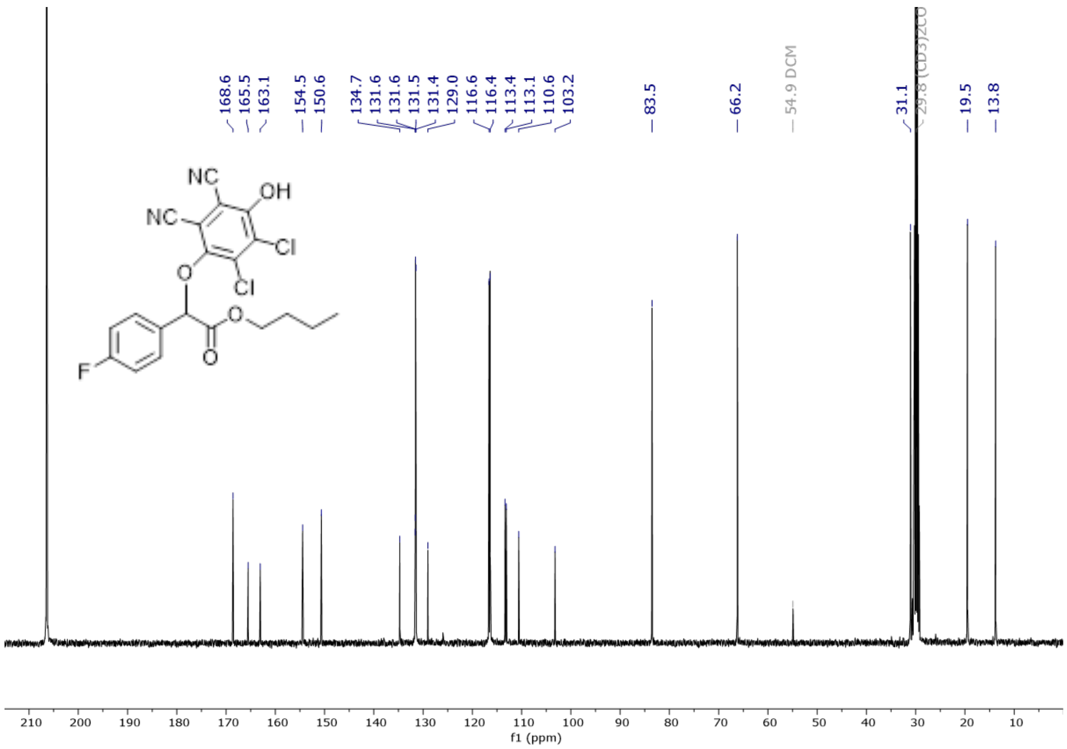

^{19}F NMR (377 MHz, Acetone-d_6)

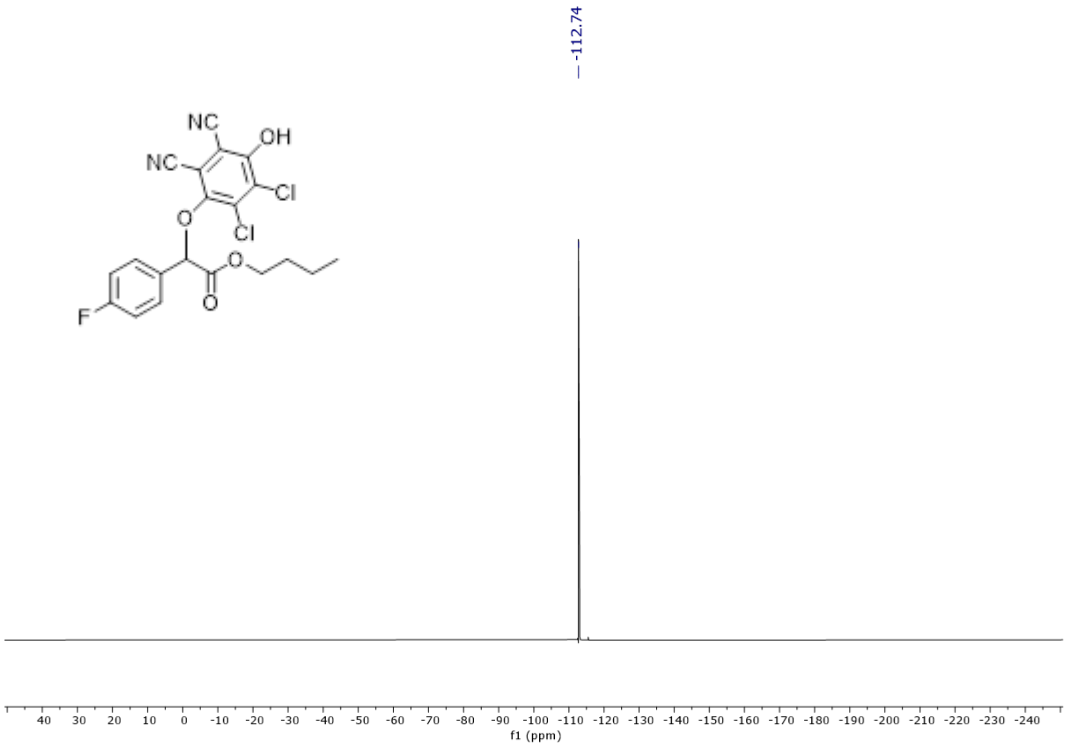

*4,5-Dichloro-3-(cyano(4-fluorophenyl)methoxy)-6-hydroxyphthalonitrile (**4s**)*

^{1}H NMR (500 MHz, Acetone-d_6)

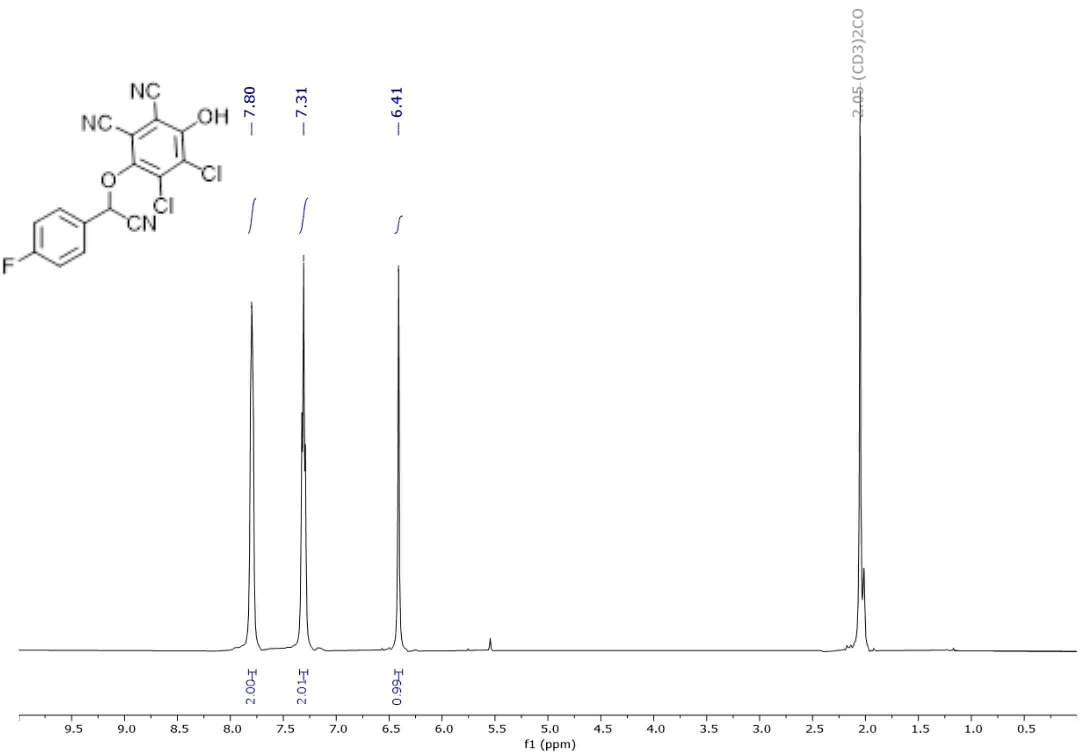

^{13}C NMR (126 MHz, Acetone-d_6)

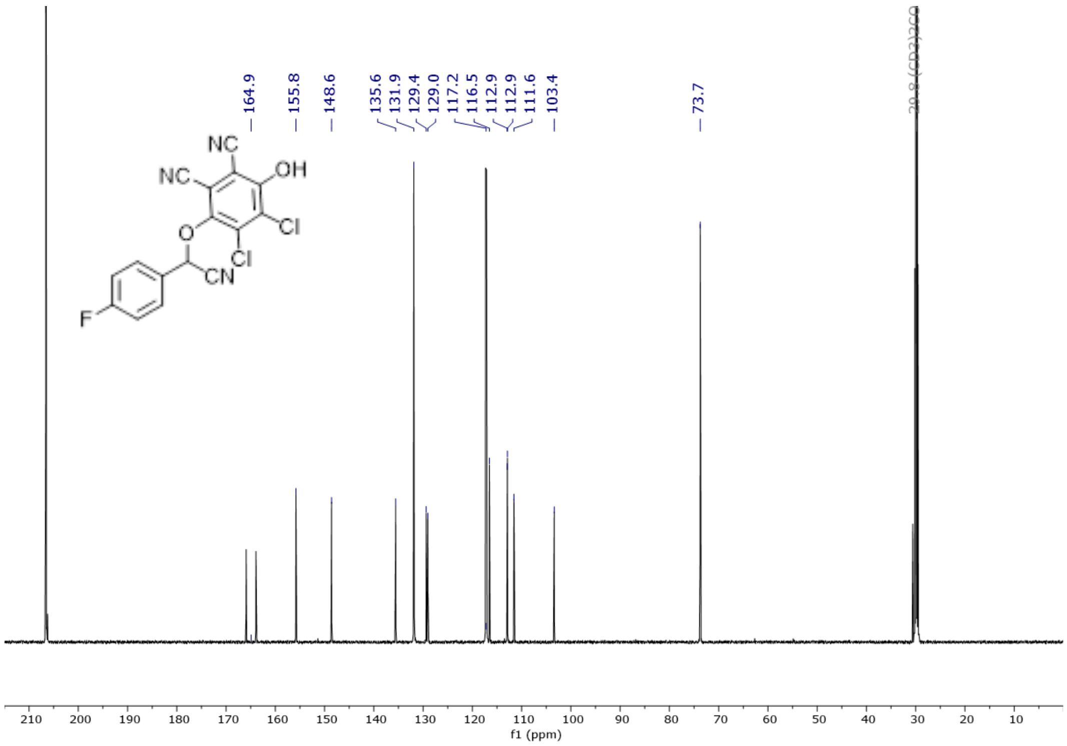

^{19}F NMR (376 MHz, Acetone-d_6)

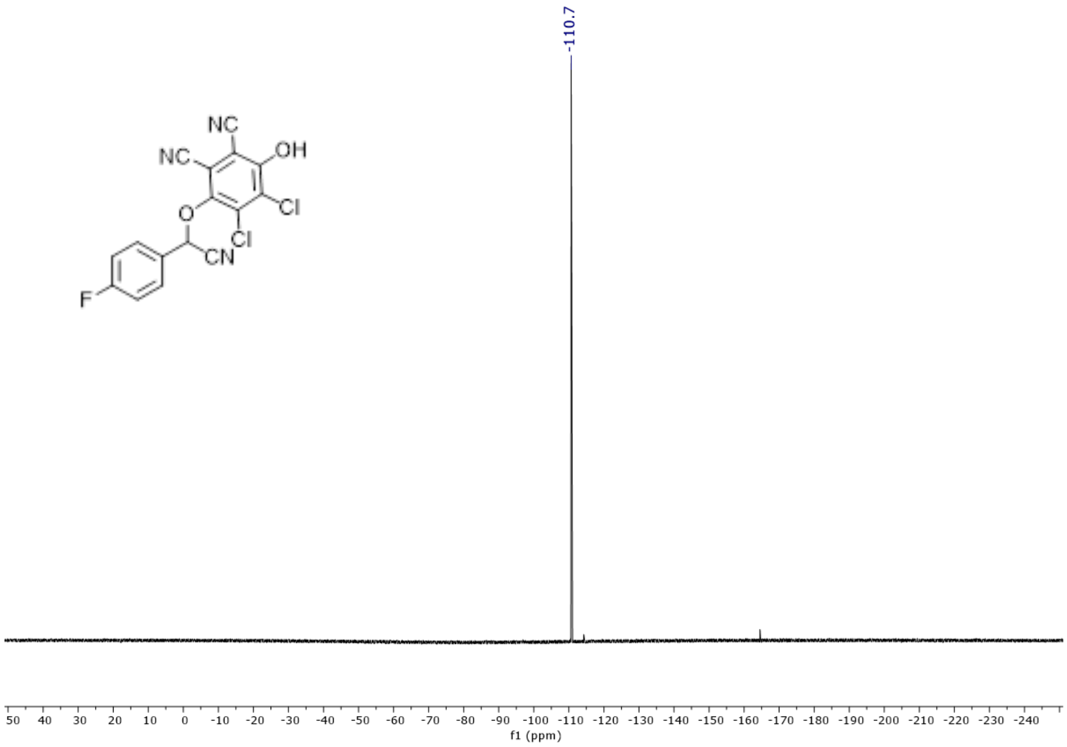

*4,5-Dichloro-3-(cyano(phenyl)methoxy)-6-hydroxyphthalonitrile (**4t**)*

¹H NMR (600 MHz, Acetone-d_6)

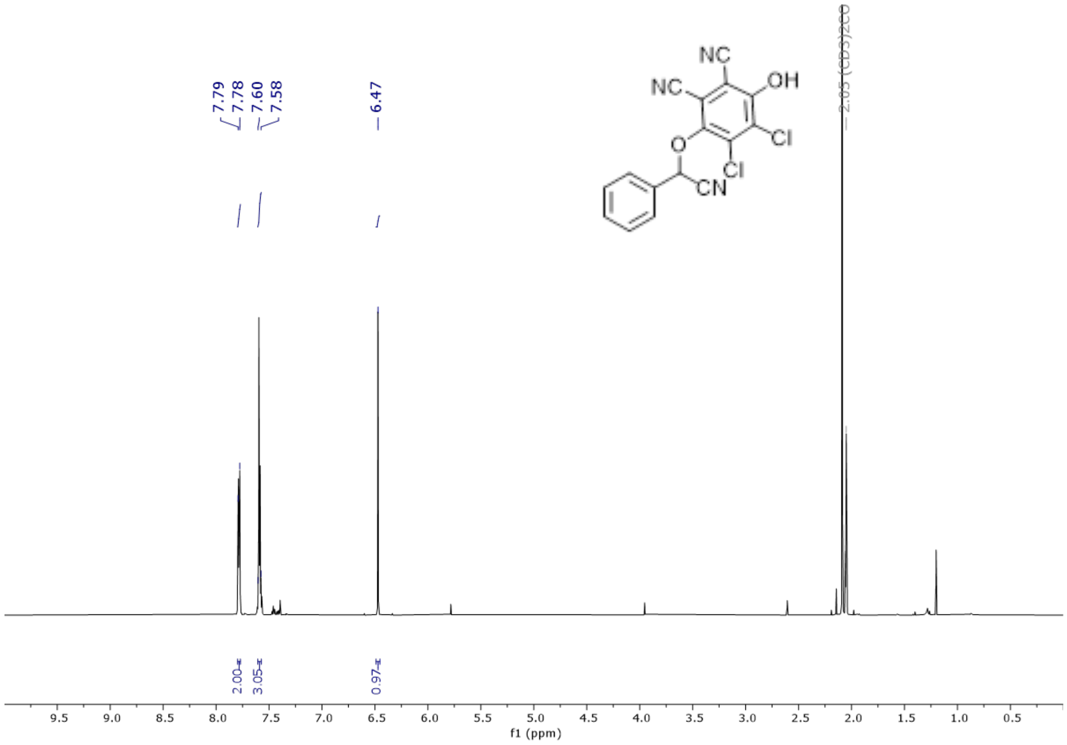

¹³C NMR (151 MHz, Acetone-d_6)

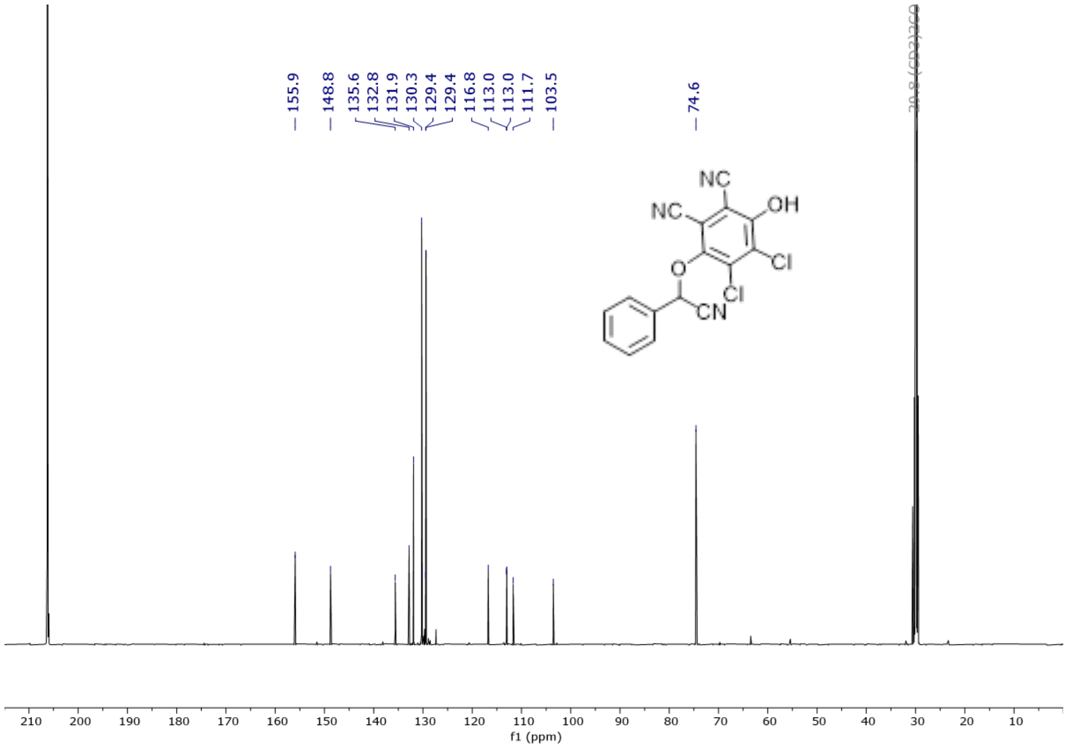

*4,5-Dichloro-3-((4-fluorobenzyl)oxy)-6-hydroxyphthalonitrile (**4w**)*

^{1}H NMR (500 MHz, Acetone-d_6)

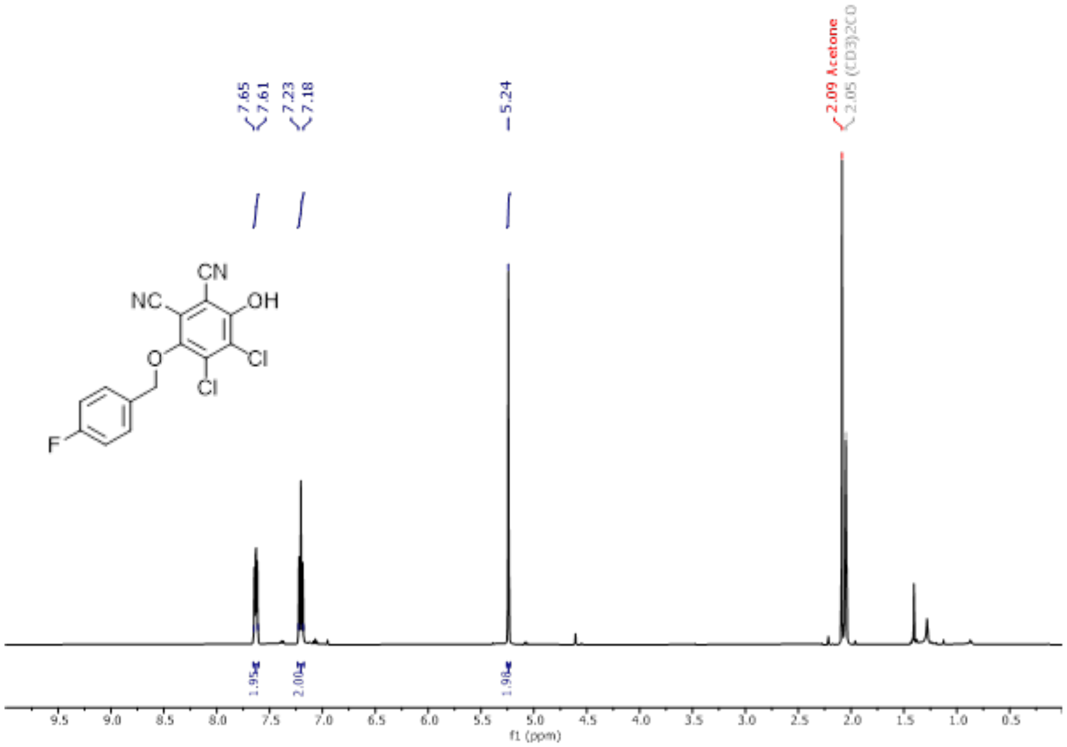

^{13}C NMR (126 MHz, Acetone-d_6)

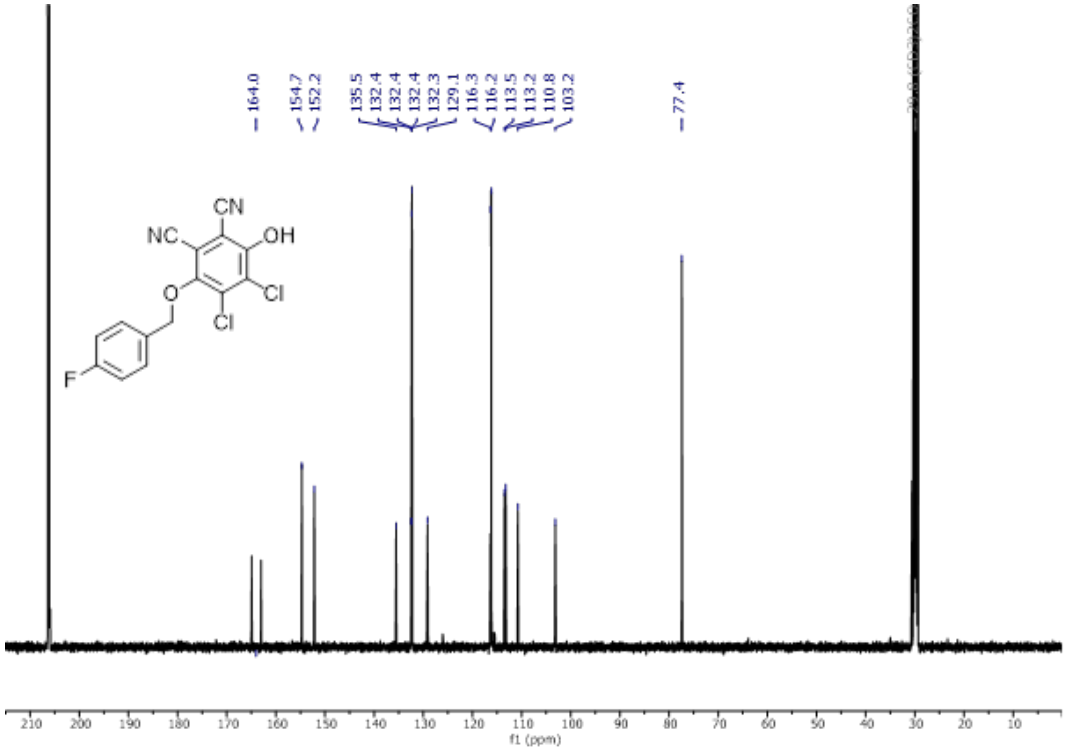

^{19}F NMR (471 MHz, Acetone-d_6)

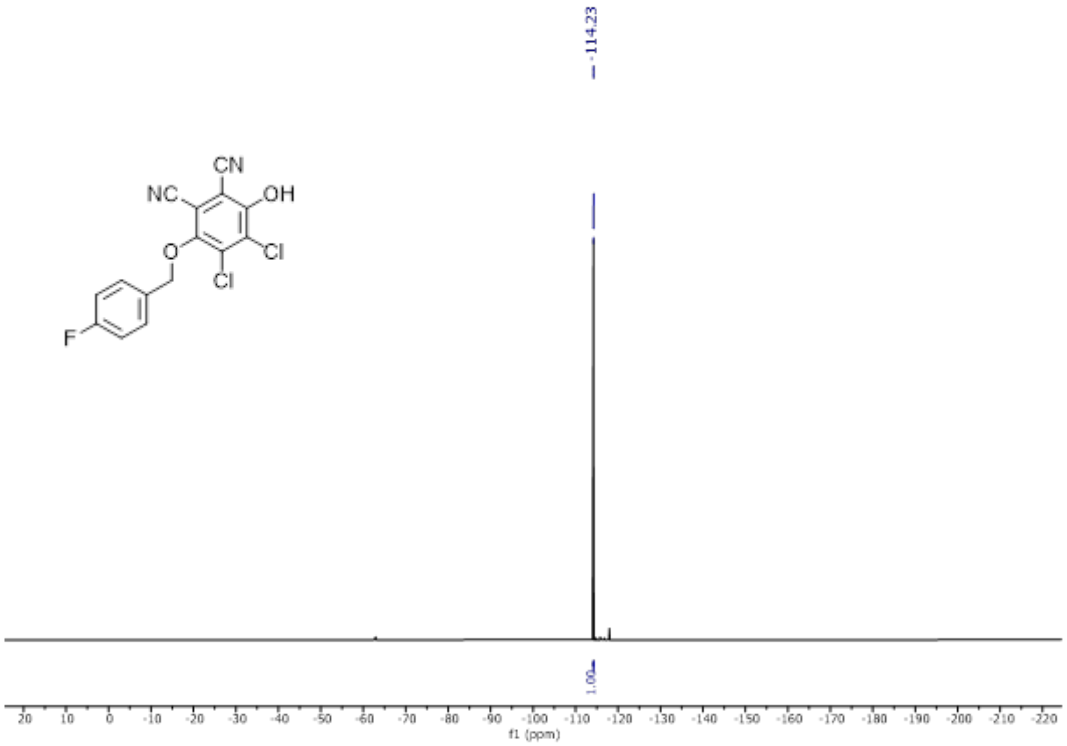

*3-((4-Bromobenzyl)oxy)-4,5-dichloro-6-hydroxyphthalonitrile (**4x**)*

^{1}H NMR (400 MHz, Acetone-d_6)

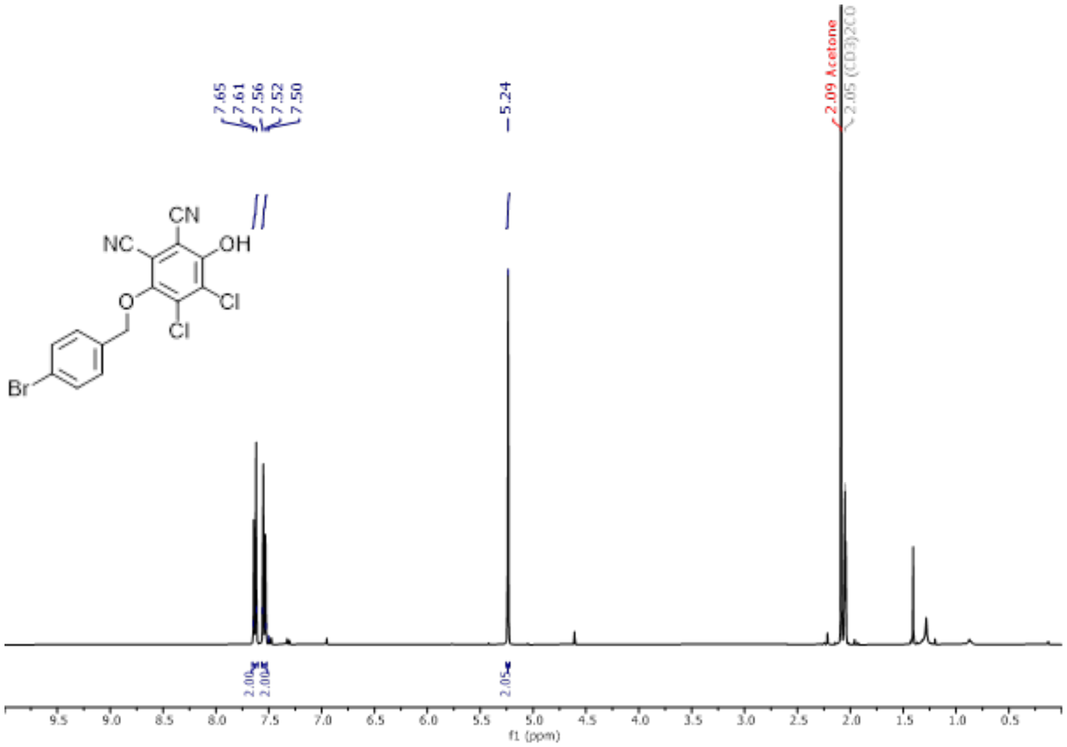

^{13}C NMR (101 MHz, Acetone-d_6)

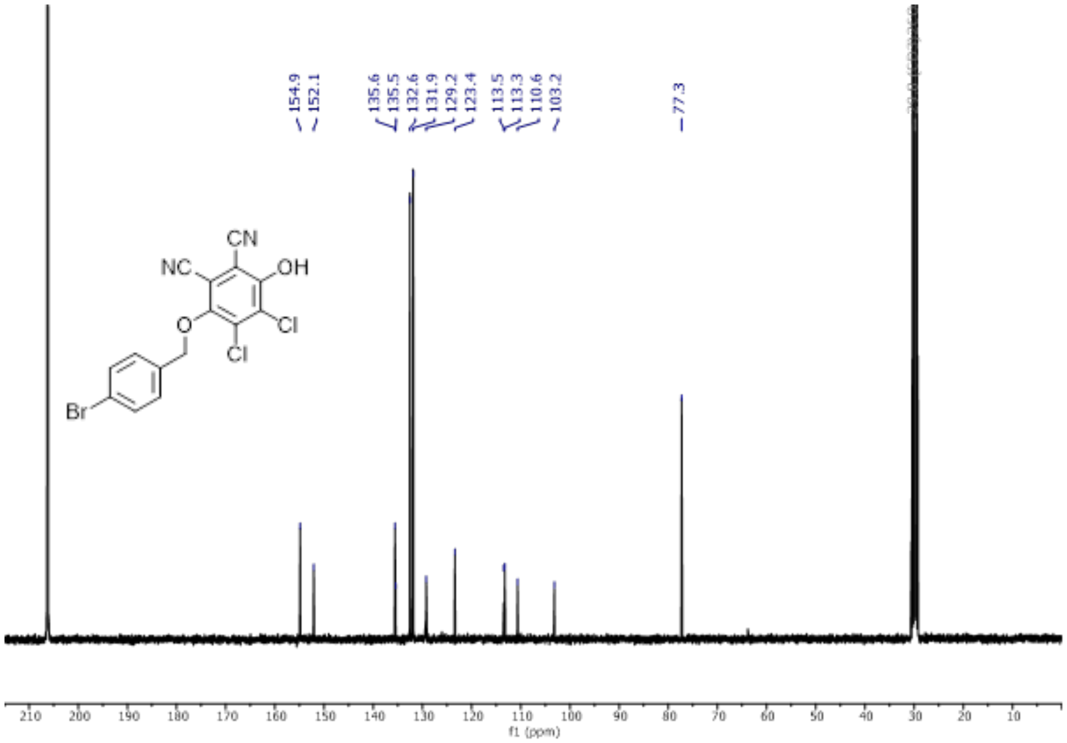

*4,5-Dichloro-3-hydroxy-6-((4-iodobenzyl)oxy)phthalonitrile (**4i**)*

^{1}H NMR (500 MHz, Acetone-d_6)

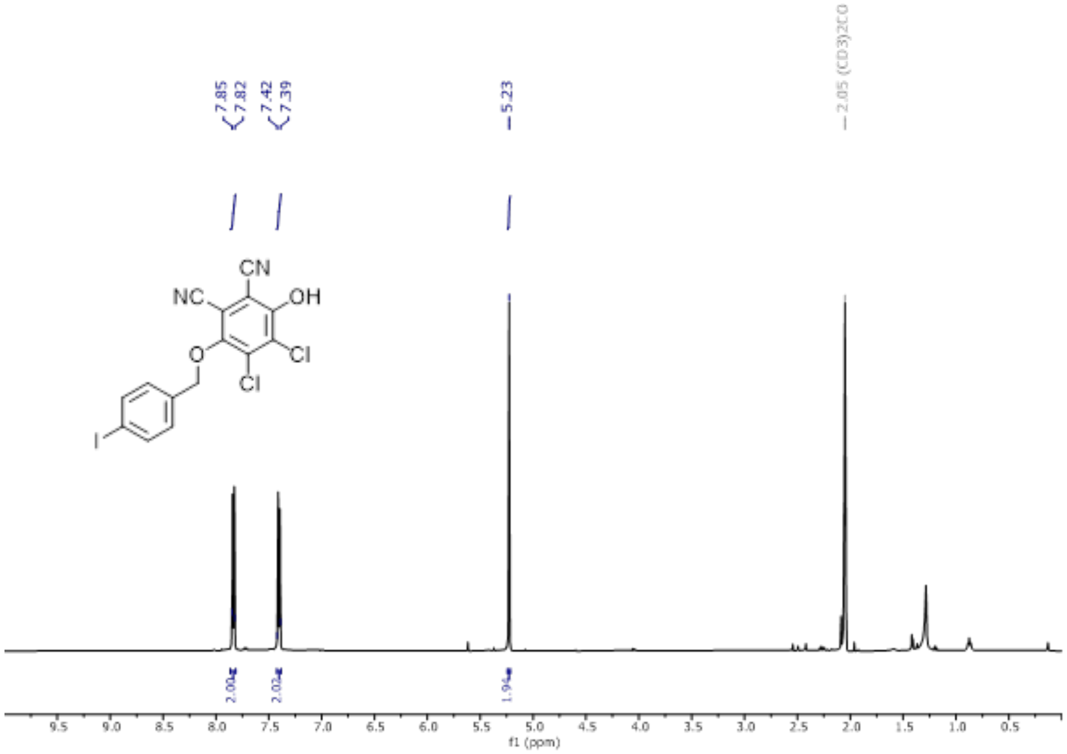

^{13}C NMR (126 MHz, Acetone-d_6)

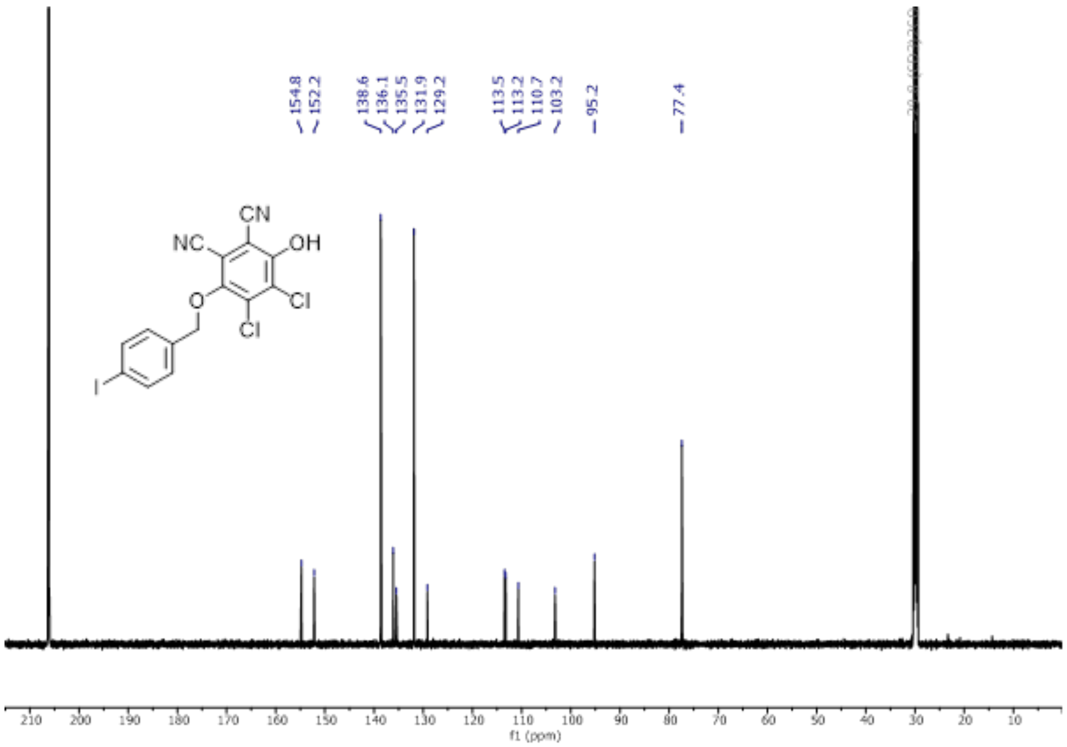

*3-((3-Bromobenzyl)oxy)-4,5-dichloro-6-hydroxyphthalonitrile (**4z**)*

^{1}H NMR (400 MHz, Acetone-d_6)

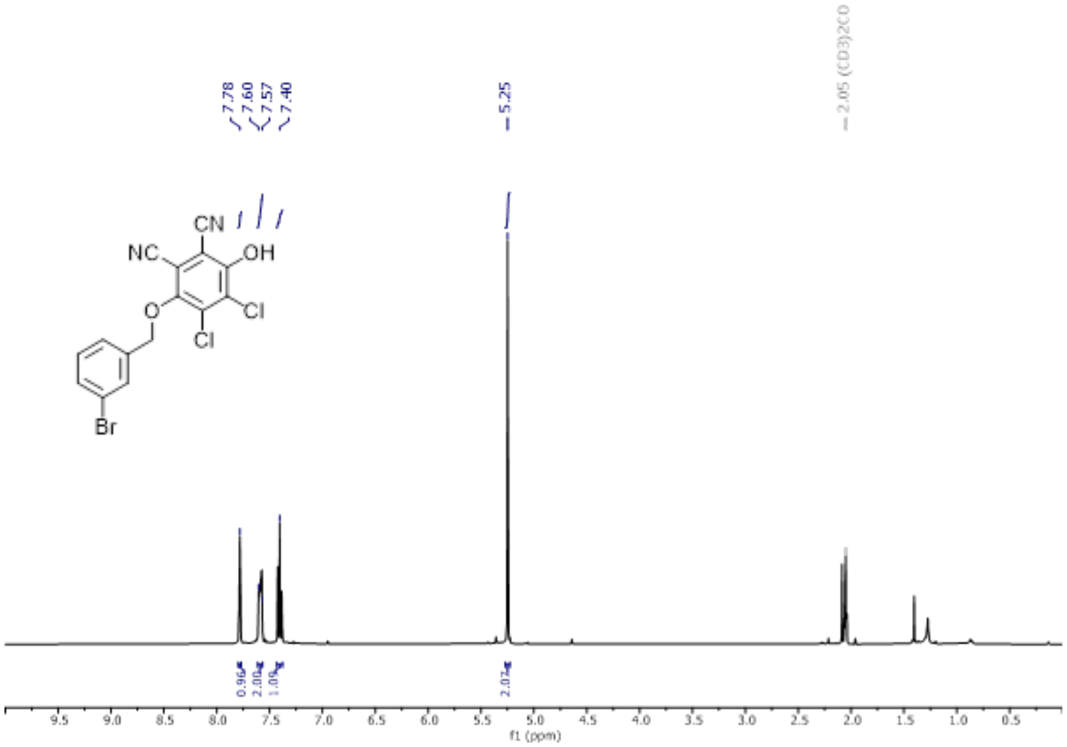

^{13}C NMR (101 MHz, Acetone-d_6)

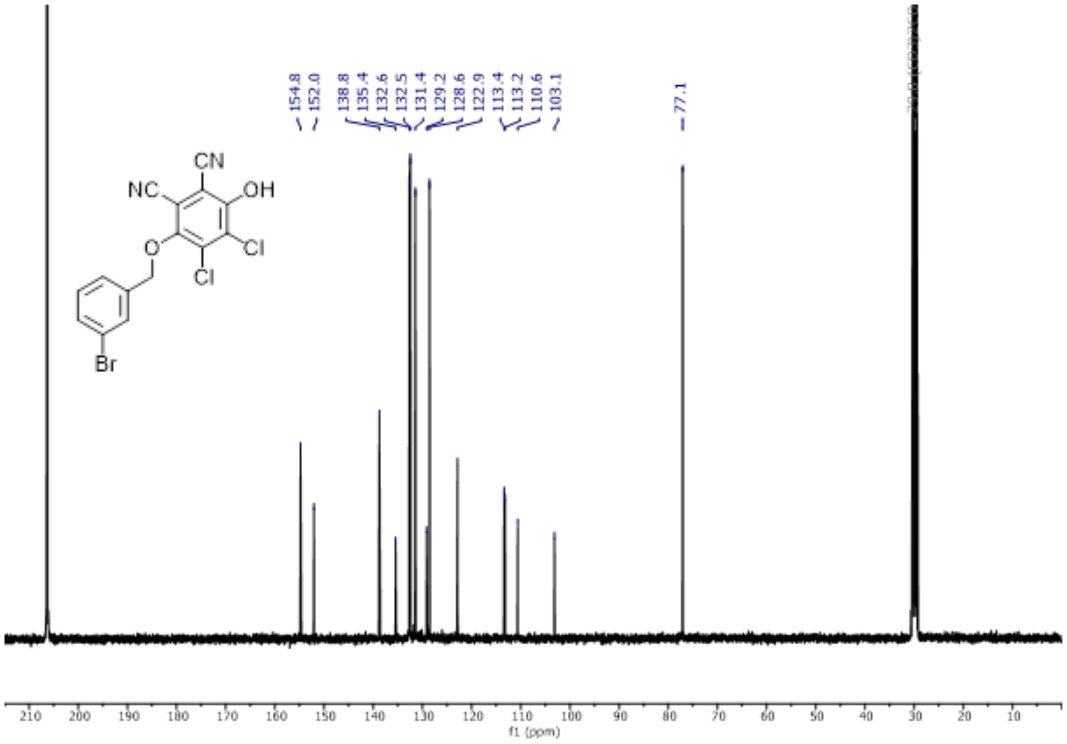

*3-((2-Bromobenzyl)oxy)-4,5-dichloro-6-hydroxyphthalonitrile (**4aa**)*

^{1}H NMR (500 MHz, Acetone-d_6)

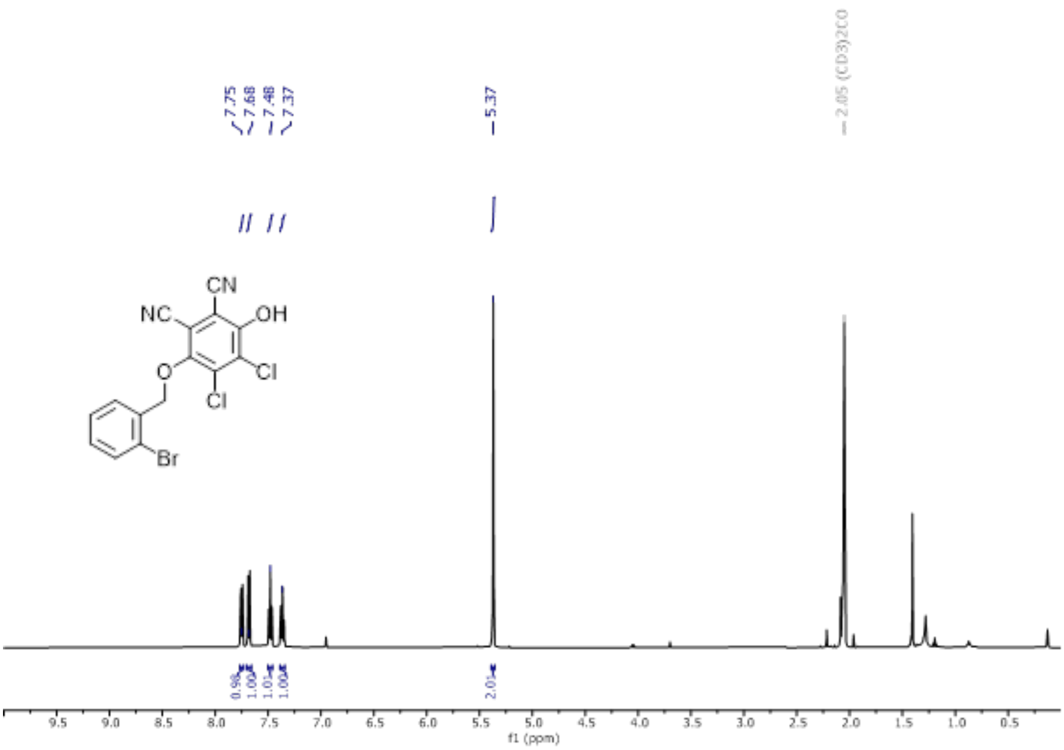

^{13}C NMR (126 MHz, Acetone-d_6)

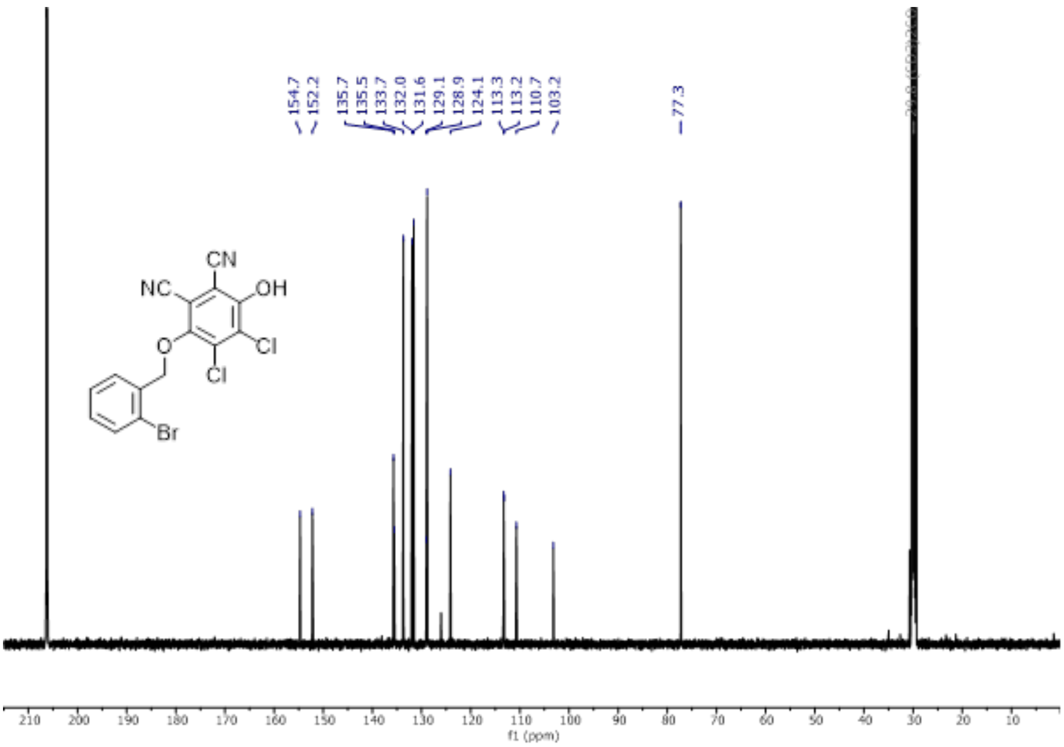

*4,5-Dichloro-3-hydroxy-6-((4'-(trifluoromethyl)-[1,1'-biphenyl]-4-yl)methoxy)phthalonitrile (**4ab**)*

^{1}H NMR (400 MHz, Acetone-d_6)

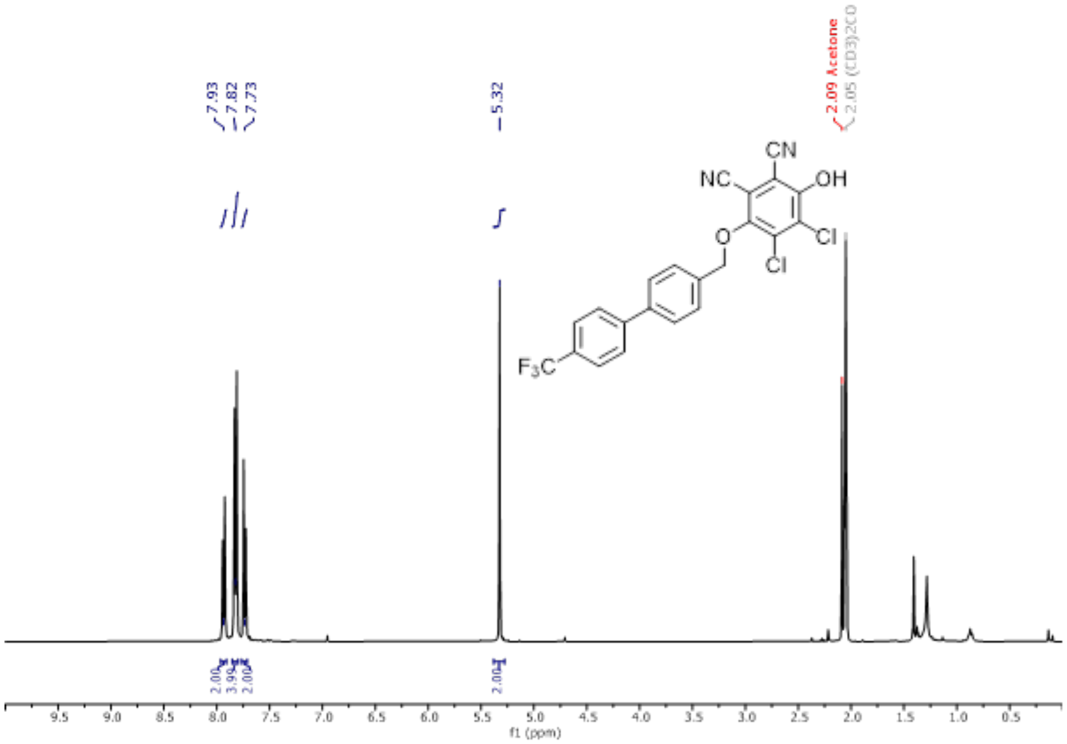

^{13}C NMR (101 MHz, Acetone-d_6)

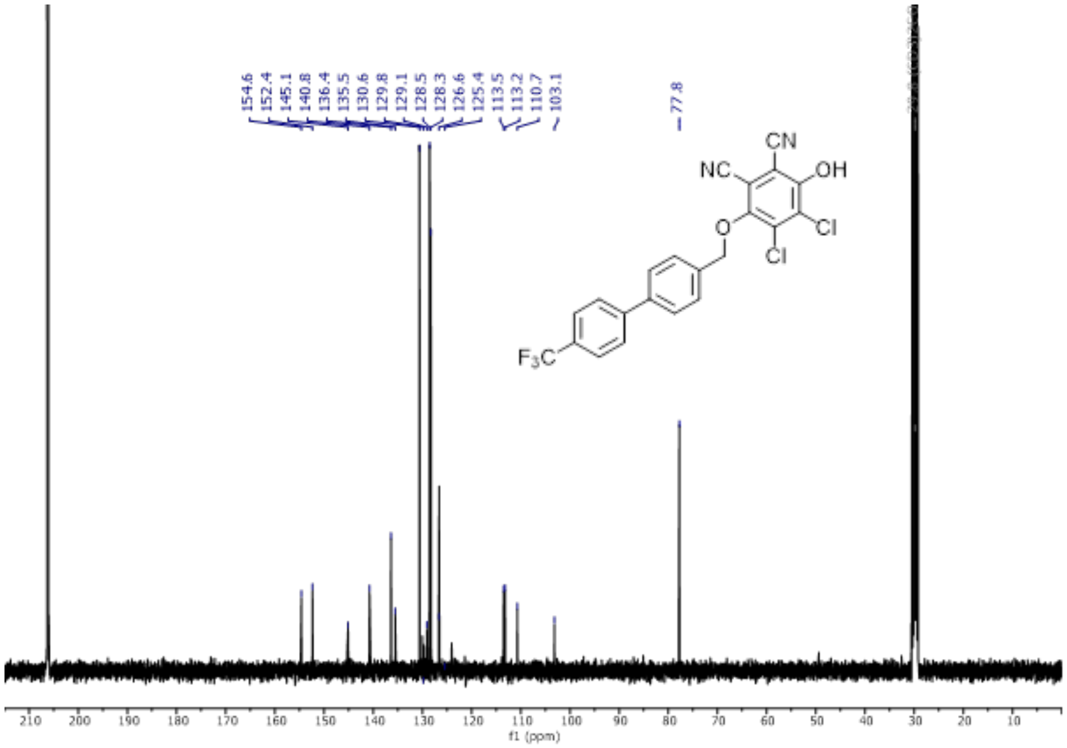

^{19}F NMR (377 MHz, Acetone-d_6)

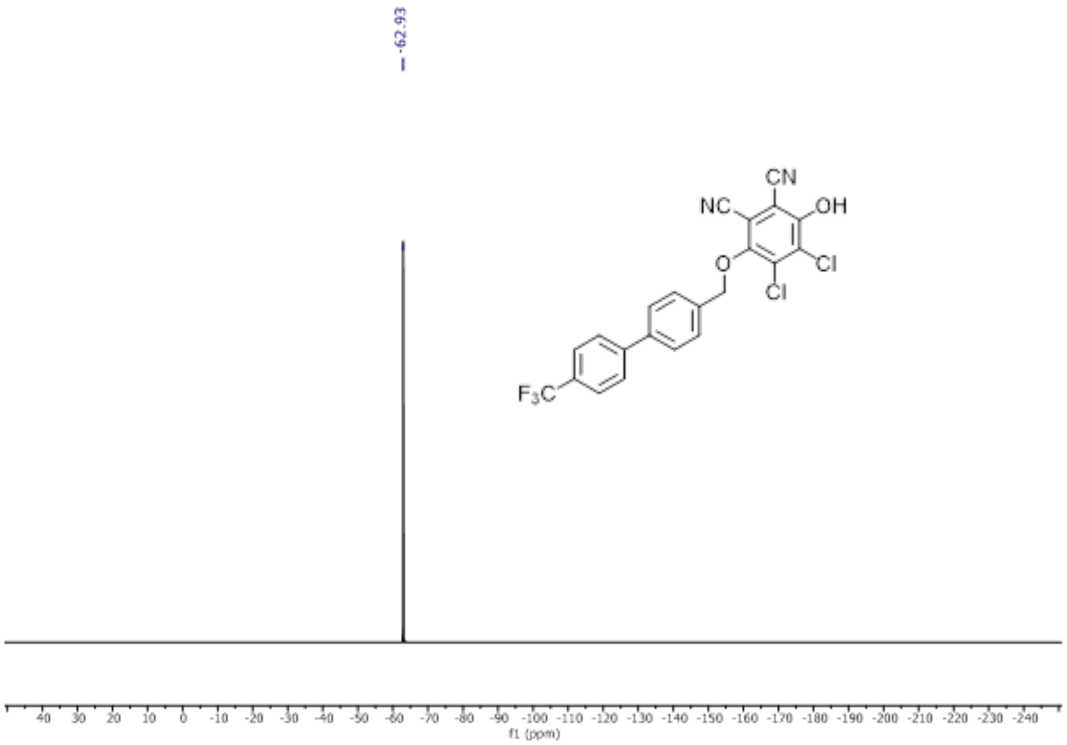

*4,5-Dichloro-3-((4'-cyano-[1,1'-biphenyl]-4-yl)methoxy)-6-hydroxyphthalonitrile (**4ac**)*

^{1}**H NMR** (600 MHz, DMSO-d_6)

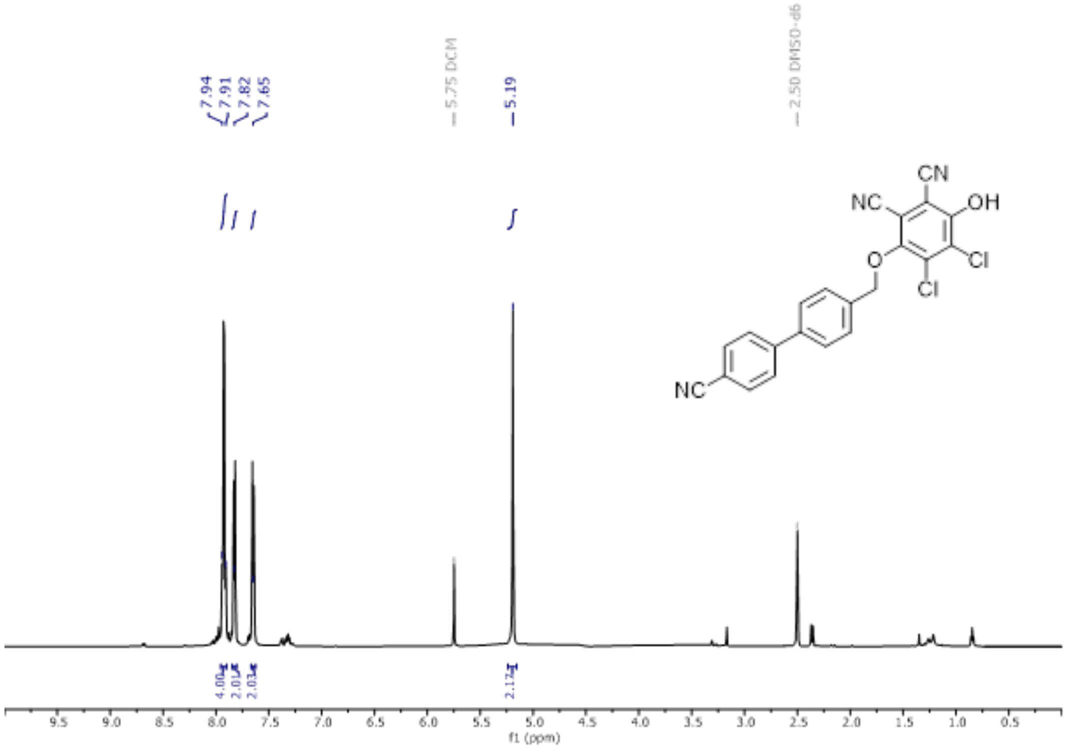

^{13}**C NMR** (151 MHz, DMSO-d_6)

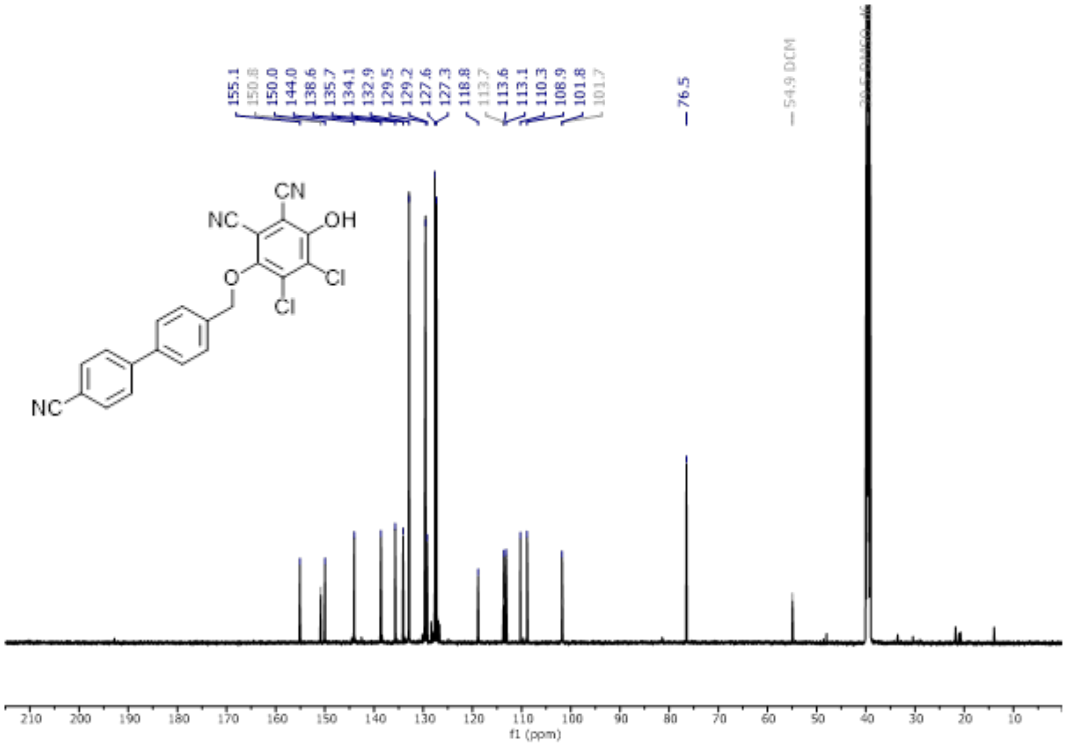

2-Chloro-2-(4-fluorophenyl)acetonitrile (**7s**)

^{1}H NMR (600 MHz, Acetone-d_6)

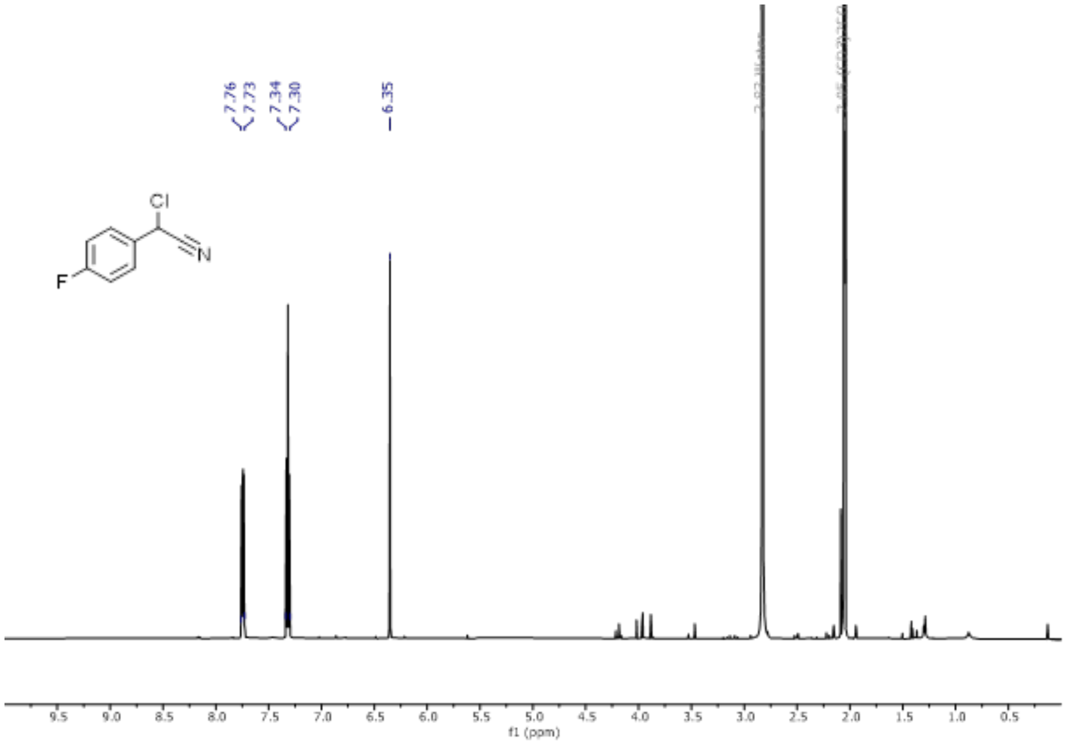

^{13}C NMR (151 MHz, Acetone-d_6)

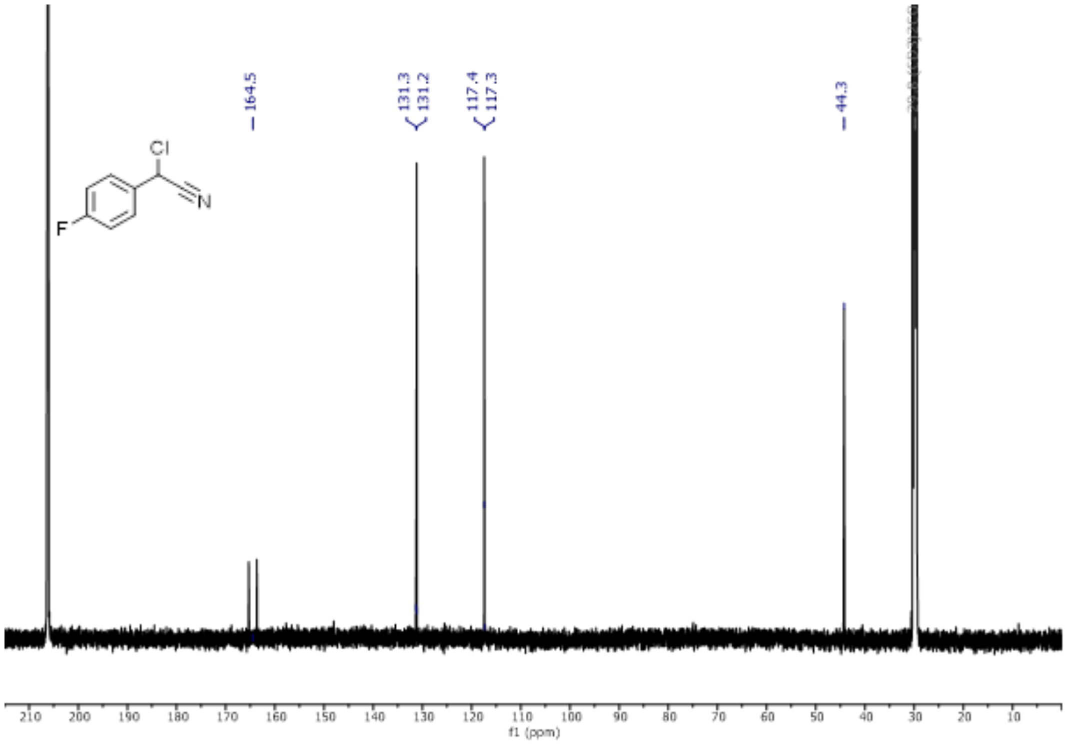

^{19}F NMR (471 MHz, Acetone-d_6)

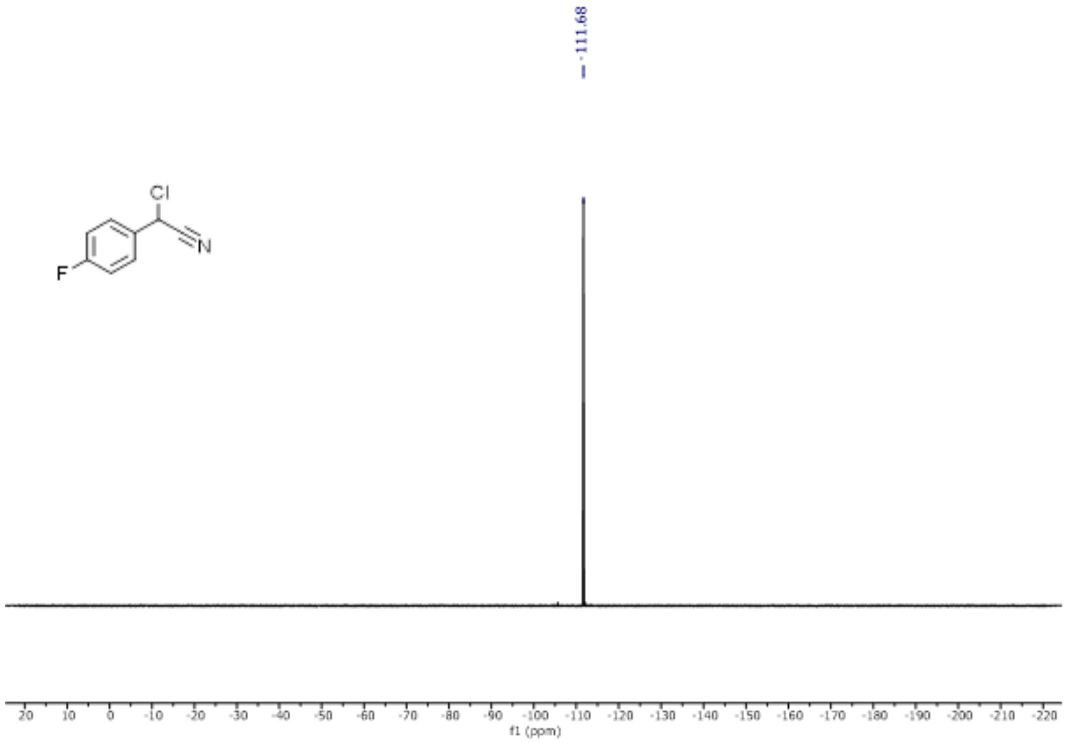

2-(1-Chloroethyl)anthracene-9,10-dione (**7f**)

^{1}H NMR (500 MHz, $CDCl_3$)

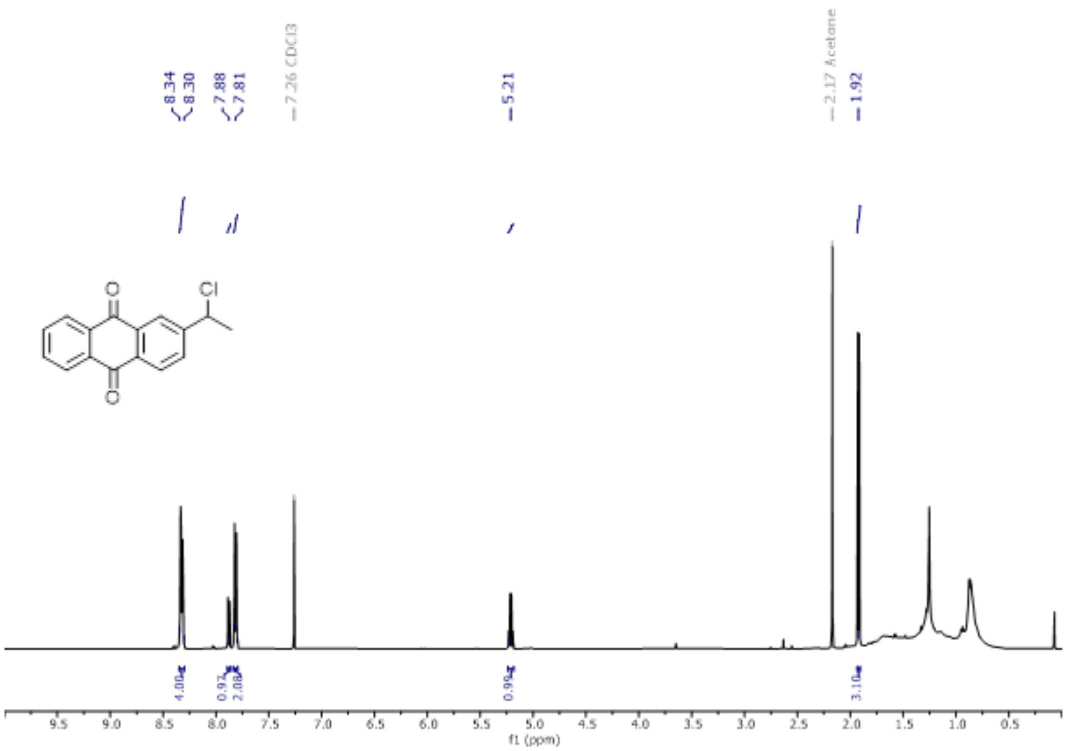

^{13}C NMR (126 MHz, $CDCl_3$)

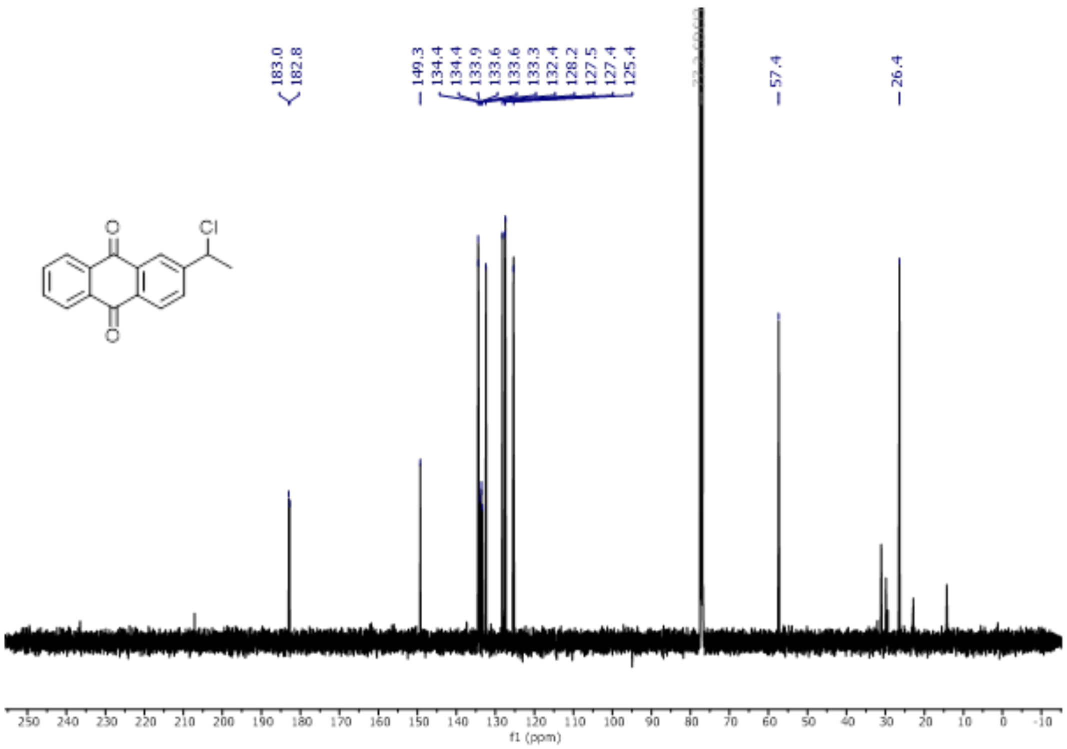

2-Bromo-2-(4-fluorophenyl)acetonitrile (**8s**)

^{1}H NMR (600 MHz, Acetone-d_6)

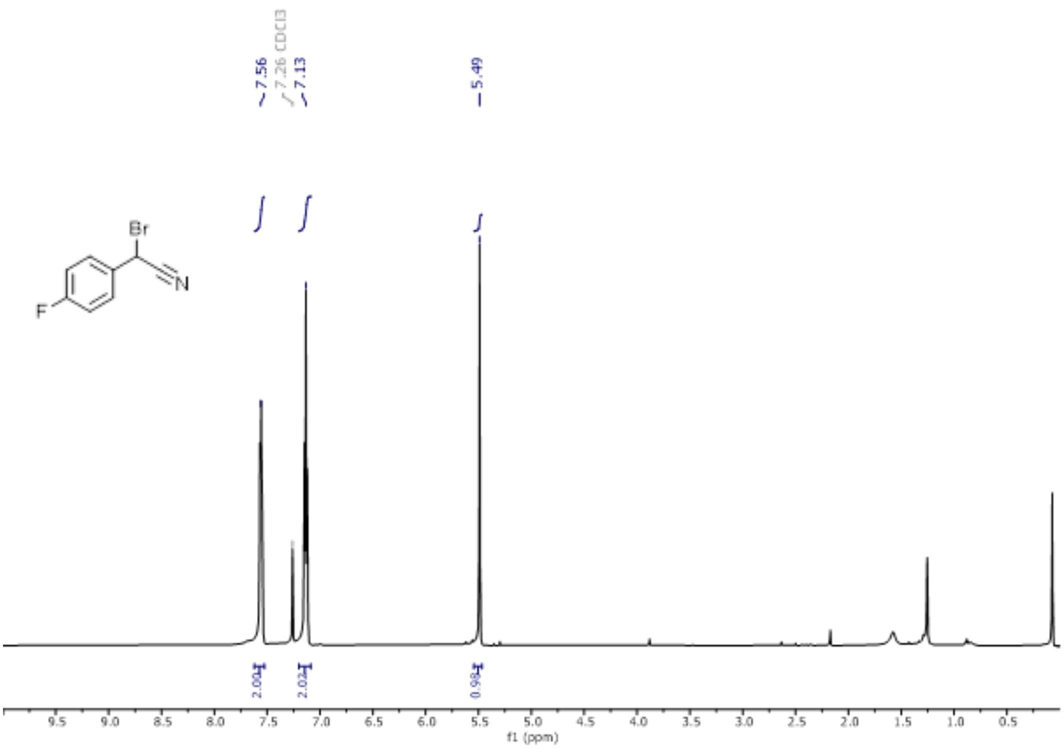

^{13}C NMR (151 MHz, Acetone-d_6)

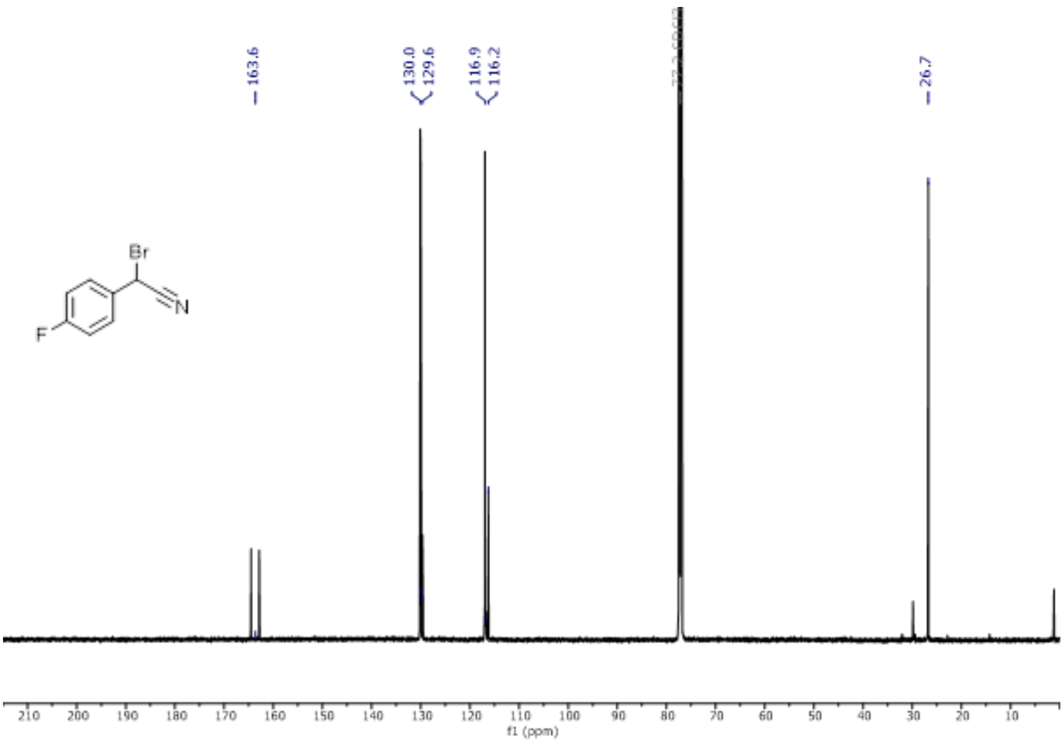

^{19}F NMR (471 MHz, Acetone-d_6)

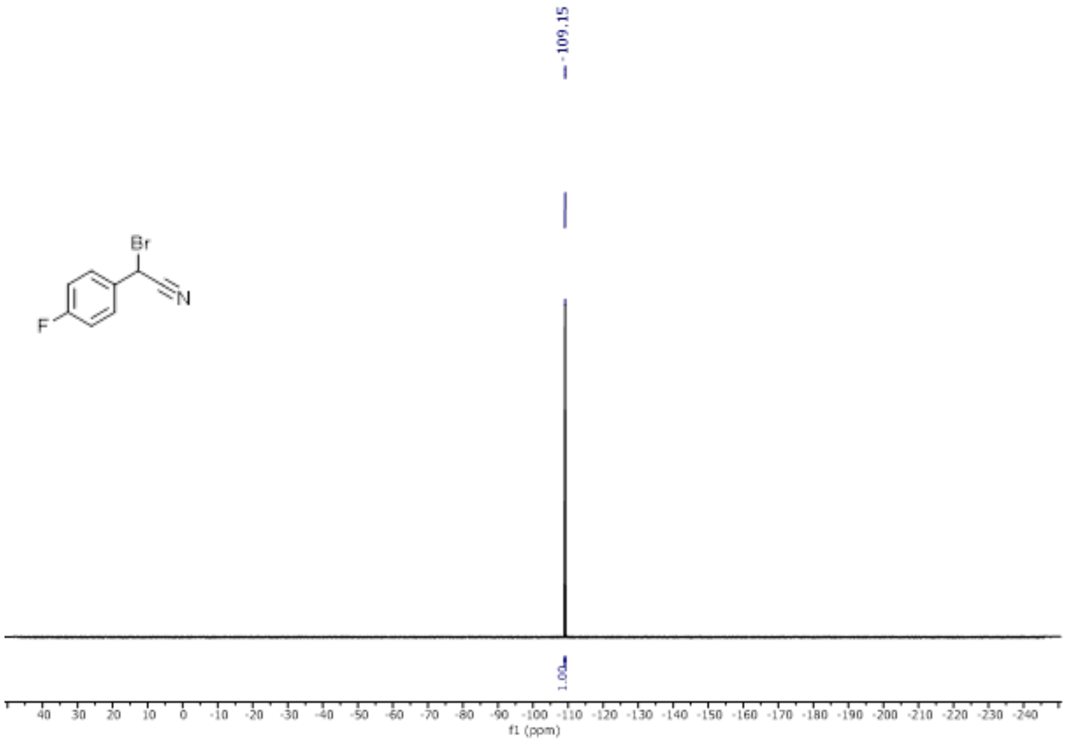

References

1 A. P. Atkins, A. C. Rowett, D. M. Heard, J. A. Tate and A. J. J. Lennox, *Org. Lett.*, 2022, **24**, 5105–5108.

2 M. R. Penny and S. T. Hilton, *J. Flow Chem.*, 2023, **13**, 435–442.

3 G. Zhang, Y. Wang, X. Wen, C. Ding and Y. Li, *Chem. Commun.*, 2012, **48**, 2979.

4 F. Song, S. H. Park, C. Wu and A. E. Strom, *J. Org. Chem.*, 2023, **88**, 3353–3358.

5 V. S. Batista, R. H. Crabtree, S. J. Konezny, O. R. Luca and J. M. Praetorius, *New J. Chem.*, 2012, **36**, 1141.

6 W. Choi, D. Harada, K. Oyaizu and H. Nishide, *J. Am. Chem. Soc.*, 2011, **133**, 19839–19843.

7 Q. Yu, D. Zhou, Y. Liu, X. Huang, C. Song, J. Ma and J. Li, *Org. Lett.*, 2023, **25**, 47–52.

8 H. H. San, S.-J. Wang, M. Jiang and X.-Y. Tang, *Org. Lett.*, 2018, **20**, 4672–4676.

9 M. Juhl, A. R. Petersen and J. Lee, *Chem. Eur. J.*, 2021, **27**, 228–232.

10 P. V. Ramachandran, A. A. Alawaed and H. J. Hamann, *Org. Lett.*, 2023, **25**, 4650–4655.

11 L. Li, M. Luo, S. Liu, J. Lv, J. Zeng, Q. Yan, J. Liao, H. Wang and S. Gu, *Eur. J. Org. Chem.*, 2025, **28**, e202401111.
